# Design and biological evaluation of substituted 5,7-dihydro-6*H*-indolo[2,3-*c*]quinolin-6-one as novel selective Haspin inhibitors

**DOI:** 10.1080/14756366.2022.2082419

**Published:** 2022-06-07

**Authors:** Sreenivas Avula, Xudan Peng, Xingfen Lang, Micky Tortorella, Béatrice Josselin, Stéphane Bach, Stephane Bourg, Pascal Bonnet, Frédéric Buron, Sandrine Ruchaud, Sylvain Routier, Cleopatra Neagoie

**Affiliations:** a Guangzhou Institute of Biomedicine and Health, Chinese Academy of Science, Guangzhou, China; b Sorbonne Université/CNRS UMR8227, Roscoff cedex, France; c Sorbonne Université/CNRS FR2424, Plateforme de criblage KISSf (Kinase Inhibitor Specialized Screening Facility), Roscoff cedex, France; d Institut de Chimie Organique et Analytique, Université d’Orléans, UMR CNRS 7311, Orleans, France; e Centre for Regenerative Medicine and Health, Hong Kong Institute of Science and Innovation, Chinese Academy of Science, Hong Kong SAR, China

**Keywords:** Indoloquinoline, Haspin kinase, docking, cell viability

## Abstract

A library of substituted indolo[2,3-*c*]quinolone-6-ones was developed as simplified Lamellarin isosters. Synthesis was achieved from indole after a four-step pathway sequence involving iodination, a Suzuki-Miyaura cross-coupling reaction, and a reduction/lactamization sequence. The inhibitory activity of the 22 novel derivatives was assessed on Haspin kinase. Two of them possessed an IC_50_ of 1 and 2 nM with selectivity towards a panel of 10 other kinases including the parent kinases DYRK1A and CLK1. The most selective compound exerted additionally a very interesting cell effect on the osteosarcoma U-2 OS cell line.

## Introduction

Marine products currently represent an underutilised source of leads for the pharmaceutical industry^1^. Besides their original and complex structures, they often offer new action modes and structural originality. Nevertheless, their low abundance and the presence of few analogues makes it difficult to obtain large libraries, perform full biological characterisation and achieve structure–activity relationship (SAR) exploration. Despite these difficulties, these products remain attractive due to their high valorisation potential, and their complex structures have prompted medicinal chemists to use disruptive strategies to intuitively isolate the pharmacophore elements that trigger biological activity.

**Figure 1.**
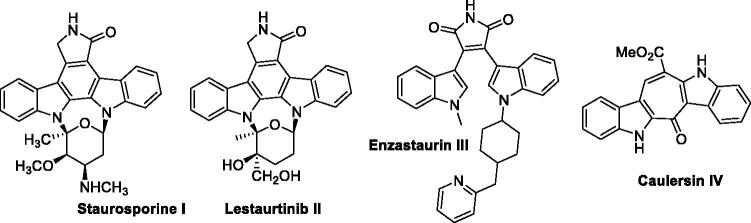
Structures of Staurosporine and its simplified derivatives, which have entered clinical trials as kinase inhibitors, and Caulersin.

For these reasons, some marine products and their synthetic analogues have emerged in drug discovery strategies and several of them have been reported for protein kinase inhibition^2^. Among them the most successful example is the polycyclic staurosporine **I**^3^^,^^4^. This lead compound has led from extraction, hemisynthesis and organic synthesis efforts to Lestaurtinib **II**^5^^,^^6^ and simplified Enzastaurin **III**^7^, two potent drugs targeting VEGF receptors and kinases, which have entered clinical trials against leukaemia and cancer (Figure 1). In this field, the chemical simplification of indolocarbazole scaffolds and caulersin **IV** have generated strong kinase inhibitors and cytotoxic agents^8–14^.

**Figure 2.**
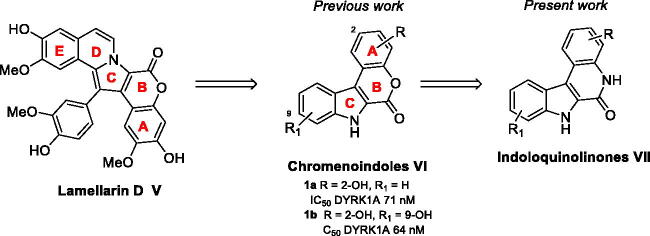
Lamellarin D, chromenoindoles, and envisioned indoloquinolinone chemical series.

Among these bis-indole series, lamellarins, a group of pyrrole alkaloids, have emerged (Figure 2)^15^^,^^16^. These compounds are a class of marine-derived natural products isolated from molluscs, ascidians and marine sponges. Nearly, 70 natural derivatives have been reported in this family which mainly contains a fused pentacyclic pyrroloisoquinoline lactone ring system^17^. Lamellarins have focussed the attention of medicinal chemists due to their diverse biological effects. Some have demonstrated cytotoxic activities and multidrug resistance (MDR) reversal in a number of cancer cell lines, as well as being confirmed inhibitors of topoisomerase I. Moreover, Lamellarins D (structure **V**, Figure 2), N and L have proved their ability to inhibit kinases such as GSK3β, DYRK1A and CDK5 in the nanomolar range^18^. Our first approach in this chemical series was based on the simplification of the synthetic model in order to discriminate the two activities, that is, diminish the topoisomerase I inhibition while retaining the kinase inhibition by fine-tuning the chemical structure.

This objective was reached by replacing the pyrrole moiety with an indole skeleton and designing new chromeno[3,4-*b*]indoles **VI** which revealed DYRK1A inhibition. In this structure, the rings of Lamellarin-D noted A, B and C were unchanged (Figure 2). Despite structural modulations, the kinase inhibition remained mainly in the sub-micromolar range except for derivatives **1a** and **1b,** which exhibited a high selective inhibition of DYRK1A but revealed instability in basic media due to the lactone. We therefore decided to develop a more robust indoloquinolinone^19–23^ series **VII** with the objective of creating a novel and druggable family of kinase inhibitors, as we envisioned that the presence of lactam combined with the NH of pyrrole would reinforce the hydrogen bond donor acceptor binding mode to the ATP active site.

Herein, we present access to the indolo[2,3-*c*]quinolone-6-one library and the evaluation of the family on a representative panel of kinases involved in CNS, inflammatory diseases and oncology. SAR are depicted and we demonstrate that in addition to retaining DYRK1A inhibition to a large extent, other molecules acting on CLK1 have also been designed. We additionally found that several compounds inhibit the Haspin kinase with an unprecedented selectivity. Molecular docking experiments were conducted to explain these results. Finally, screening on a cancer cell line was carried out and results showed that the compounds induced cellular effects and affect osteosarcoma cell lines in particular.

## Chemistry

**Scheme 1.**
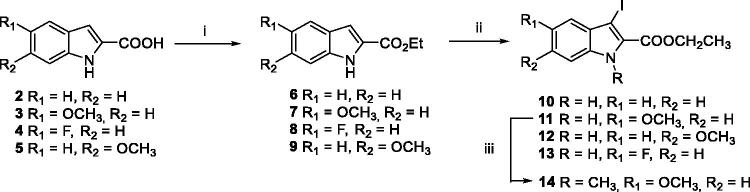
Reagents and conditions: (i) EtOH, conc. H_2_SO_4_ 10 mol%, reflux, 12 h, **6–9** quant.; (ii) I_2_ (1.5 equiv.), KOH (4.0 equiv.), DMF, r.t., 4 h; (iii) NaH (1.5 equiv.), CH_3_I (1.2 equiv.), DMF, 0–20 °C.

In order to introduce ring A on the indole, a Suzuki-Miyaura reaction appeared to be the most appropriate. The 3-iodoindole-2-carboxylic ethyl esters **10–13** were therefore prepared after 2 efficient steps. The first one consisted in the quantitative esterification of **2–4** using 10 mol% of sulphuric acid catalyst in ethanol, followed by iodination in presence of KOH to afford the attempted ethyl 3-iodo-1*H*-indolo-2-carboxylates^24^. Noteworthy, the *N*-methylation of indole **11** with a slight excess of iodomethane in presence of sodium hydride gave **14** in a near quantitative yield (Scheme 1).

**Scheme 2.**
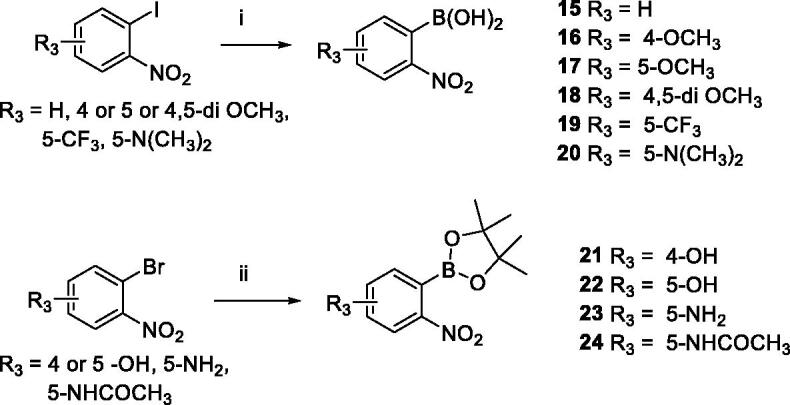
Reagents and conditions: (i) PhMgCl 2 M in THF (1.2 equiv.), B(OCH_3_)_3_ (1.2 equiv.), 30 min. at -78 °C, then aq. HCl 2 M at -10 °C, 10 min; (ii) PdCl_2_(dppf) (0.03 equiv.), KOAc (2.0 equiv.), Bis-(pinacolato)diboron (1.5 equiv.), 1,4-dioxane/H_2_O 10/1, 80 °C, 14 h.

Next, we focussed on preparing the second partner for the cross coupling reaction, that is, the unavailable boronylated nitrobenzene derivatives (Scheme 2). When the starting 2-halogeno nitrobenzenes do not contain any acidic proton, the use of Grignard reagent is recommended in the literature^25^. The reaction was first carried out with phenyl magnesium chloride (1.2 equiv.) in presence of methylorthoborate as electrophile whereas a final acidic hydrolysis led to the desired 2-nitroaryl boronic acids **15–20** in fair good yields. When 2-halogeno nitrobenzenes bear an acidic proton, the Miyaura borylation reaction can be used with conditions involving PdCl_2_(dppf) as catalyst and potassium acetate as a base in dioxane. This method furnished pinacol boronic esters **21**–**24** with modest yields after 14 h of reaction at 80 °C. Due to their sensitivity during the purification step, the boron derivatives were used in the cross coupling reaction as crude materials.

Assembly of the two building blocks was next performed using the Suzuki-Miyaura reaction in presence of PdCl_2_(dppf) and potassium carbonate as base in a refluxing mixture of water and dioxane. These conditions proved fully suitable to achieve all the envisioned cross couplings and the desired derivatives were isolated with yields ranging between 40 and 60% after 12 h of reaction (Table 1). Finally, we thought that the large library of final compounds **45**–**64** could be obtained by a “one pot” strategy (Table 2). To this end, the nitro derivatives **25–44** were also treated with iron powder in refluxing acetic acid and the *in situ* formed amine concomitantly formed the lactam ring by annelation with the nearby ethyl ester. An original library of substituted 5,7-dihydro-6*H*-indolo[2,3-*c*]quinolin-6-ones **45–65** was obtained with very high yields.

**Table 1.** Suzuki-Miyaura reaction using **10–14** and boronylated derivatives **15–24**. 
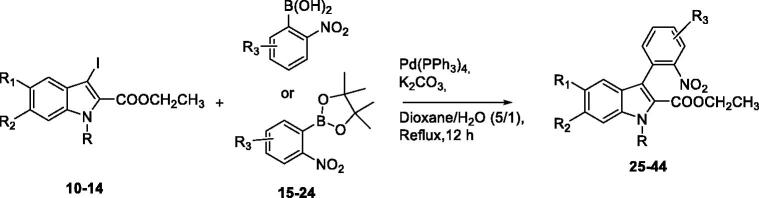

Entry	Starting materials	Product^a^(Formula/number)	Entry	Starting materials	Product^a^(Formula/number)
1	**10 **+** 15**	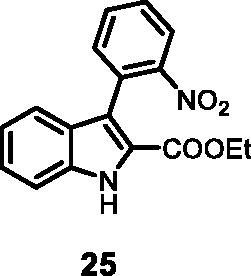	11	**11 **+** 22**	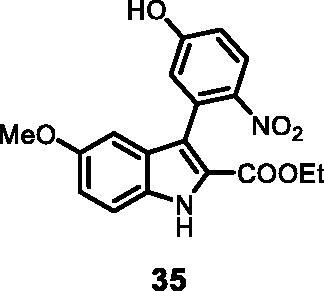
2	**10 **+** 17**	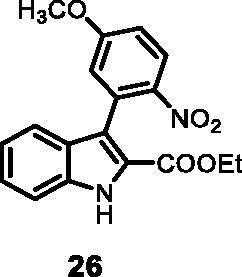	12	**11 **+** 23**	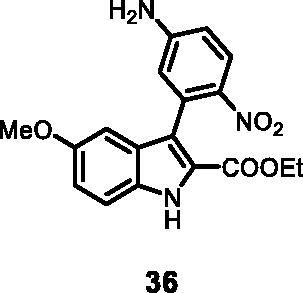
3	**10 **+** 21**	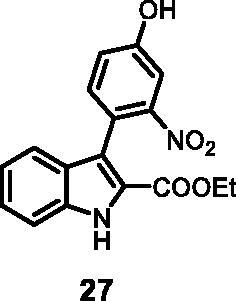	13	**11 **+** 24**	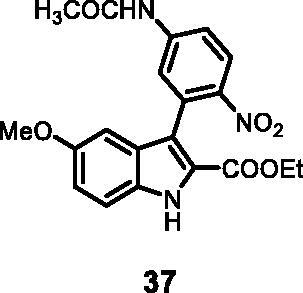
4	**10 **+** 22**	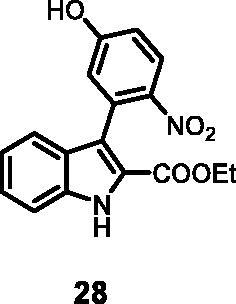	14	**12 **+** 15**	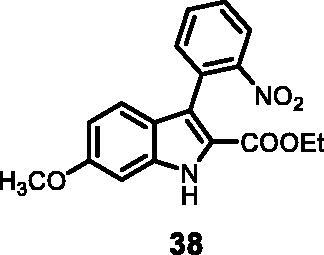
5	**11 **+** 15**	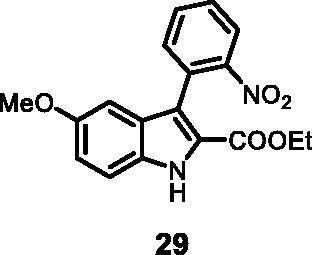	15	**12 **+** 17**	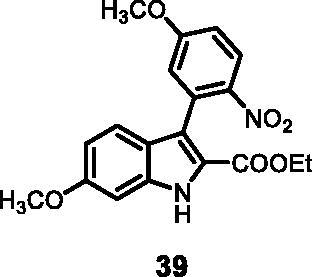
6	**11 **+** 16**	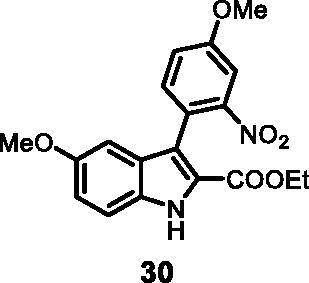	16	**13 **+** 17**	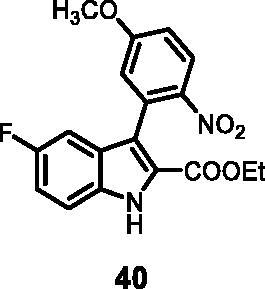
7	**11 **+** 17**	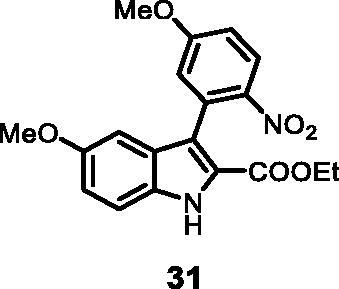	17	**13 **+** 22**	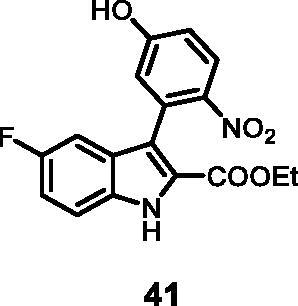
8	**11 **+** 18**	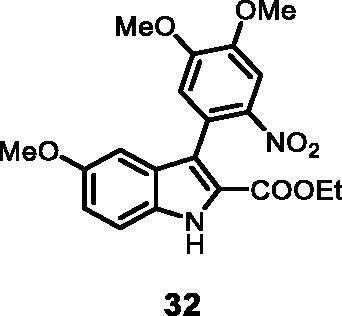	18	**14 **+** 22**	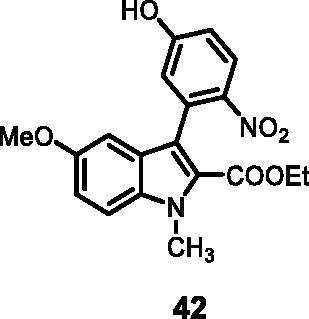
9	**11 **+** 19**	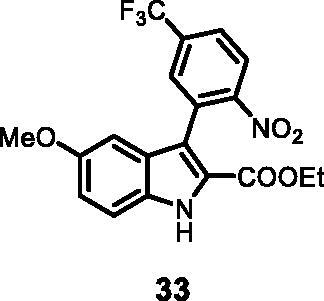	19	**14 **+** 19**	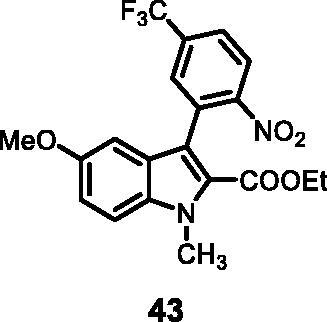
10	**11 **+** 21**	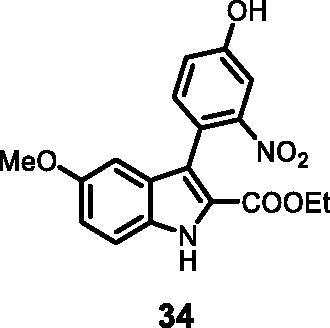	20	**14 **+** 20**	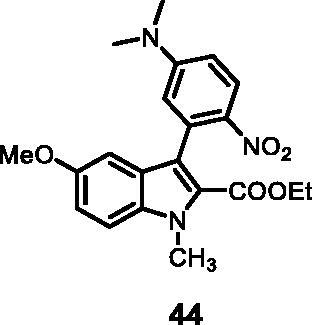

^a^Yields are indicated in the experimental section.

**Table 2.** “One pot” reduction of nitro group and lactam formation of derivatives **25–44** leading to final compounds **45–64**. 
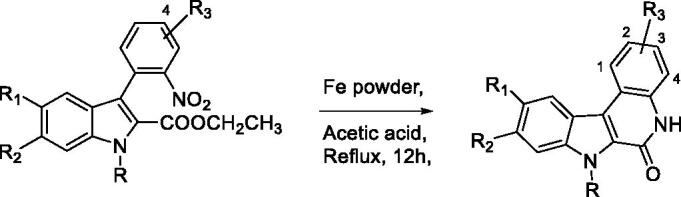

Entry	Starting materials	Product^a^(Formula/number)	Entry	Starting materials	Product^a^ (Formula/number)
1	**25**	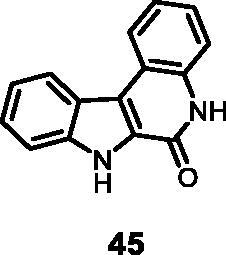	11	**35**	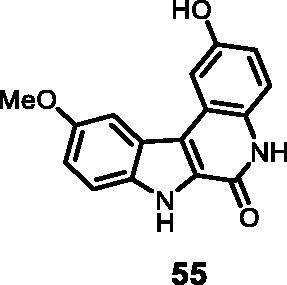
2	**26**	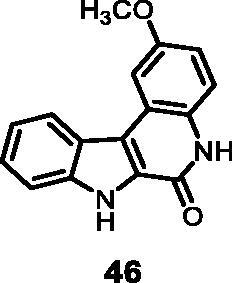	12	**36**	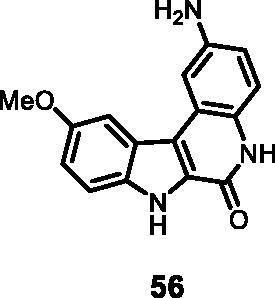
3	**27**	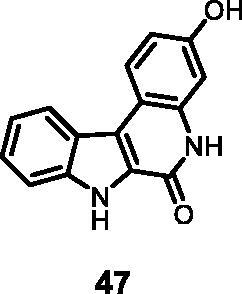	13	**37**	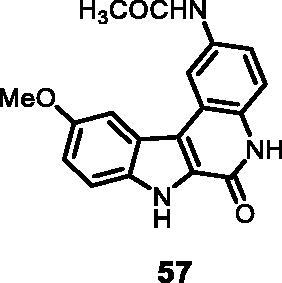
4	**28**	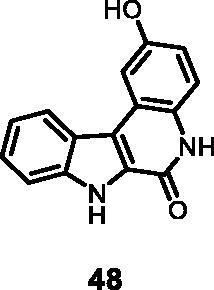	14	**38**	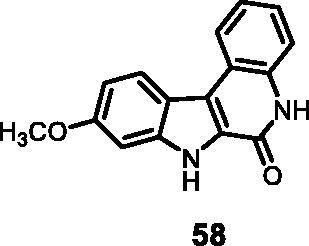
5	**29**	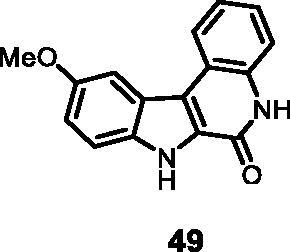	15	**39**	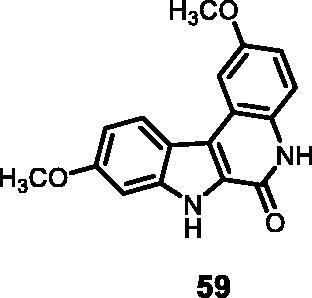
6	**30**	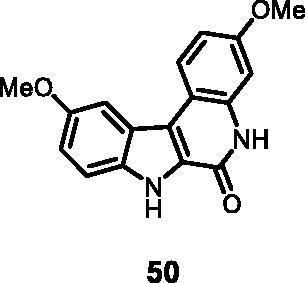	16	**40**	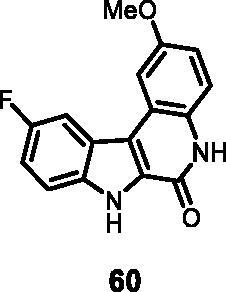
7	**31**	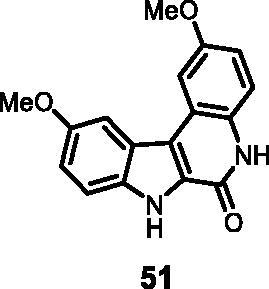	17	**41**	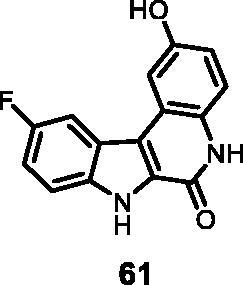
8	**32**	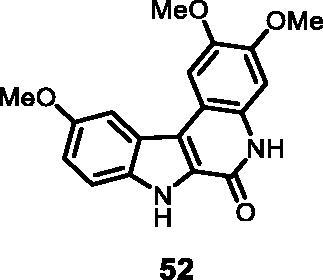	18	**42**	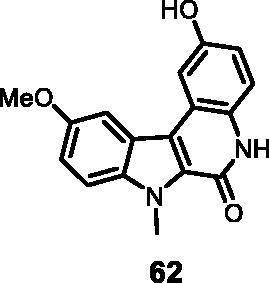
9	**33**	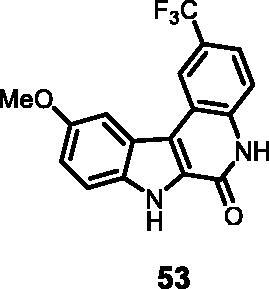	19	**43**	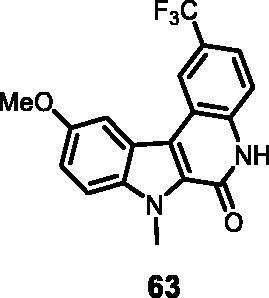
10	**34**	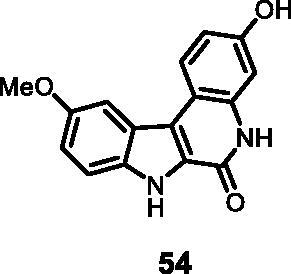	20	**44**	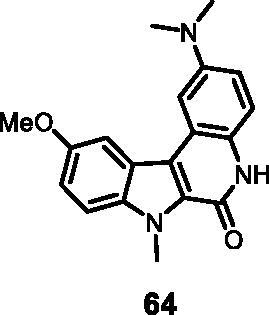

^a^Yields are indicated in the experimental section.

## Kinase assays, SAR

We previously showed that analogues of Lamellarin D, chromeno[3,4-*b*]indoles, had the ability to inhibit the DYRK1A kinase^15^ and it has been shown by different groups that DYRK inhibitors often cross-react with CLKs and with the mitotic kinase Haspin^26^^,^^27^. We therefore tested the inhibitory activity of the 22 synthesised derivatives on the *Hs*Haspin, *Mm*CLK1 and *Rn*DYRK1A recombinant kinases (Table 3).

**Table 3.** Residual activity on a representative panel of 8 protein kinases at 1 μM.

Entry	Compound	% Residual activity at 1 μM^a^							
		Haspin	CLK1	DYRK1A	CDK2	CDK5	CDK9	GSK3β	PIM1
1	**45**	44	67	61	100	100	63	100	86
2	**46**	**6**	**13**	**18**	80	78	62	86	32
3	**47**	28	22	33	61	59	20	47	37
4	**48**	**1**	**4**	**12**	84	87	49	100	63
5	**49**	**2**	**5**	**7**	73	77	40	39	41
6	**50**	**3**	**10**	**5**	77	81	51	44	19
7	**51**	**1**	**5**	**14**	62	70	33	29	36
8	**52**	**2**	**1**	**5**	58	63	37	22	29
9	**53**	33	11	36	76	56	56	63	31
10	**54**	**0**	**11**	**2**	35	59	18	48	9
11	**55**	**0**	**14**	**2**	38	53	17	20	22
12	**56**	**2**	**3**	**7**	72	79	37	39	65
13	**57**	**3**	**2**	**10**	100	100	72	58	37
14	**58**	59	49	55	62	55	55	51	48
15	**59**	**13**	**12**	**21**	74	51	41	41	34
18	**60**	**9**	**31**	**44**	97	100	100	100	100
19	**61**	**0**	**2**	**9**	57	91	41	71	38
20	**62**	**0**	**0**	**2**	31	22	6	31	75
21	**63**	51	19	29	67	68	45	44	42
22	**64**	39	11	19	100	100	59	81	82

^a^Residual kinase activity was determined at 1 μM concentration for each compound. The data mean (*n* = 2) expressed as percentage of maximum activity of the DMSO control.

The selectivity of each derivative was also determined on a representative kinase panel including *Hs*Cdk2/Cyc A, *Hs*Cdk5/p25, *Hs*Cdk9/Cyc E, *Hs*GSK3β and *Hs*PIM1. Interestingly, more than half of the analogues displayed very high activity towards Haspin with a percentage of residual activity at 1 μM close to zero (ranging from 6% for compound **46** to 0% for compounds **61**, **54**, **55,** and **62**). Apart from 5 compounds (**45**, **58**, **47**, **53, 63,** and **64)**, 17 novel indoloquinolinones showed an interesting selectivity towards Haspin, CLK1 and DYRK1A kinases with moderate activity on CDKs, GSK-3β and PIM1.

IC_50_ for Haspin, CLK1 and DYRK1A were next calculated for 14 compounds showing residual kinase activity ≤25% on Haspin kinase at a concentration of 1 μM (Table 4). It clearly appeared that the lactam moiety favoured DYRK1A inhibition. While the hydroxylated derivatives **1a** and **1b** displayed good activity on DYRK1A, they are nevertheless the only molecules in their family to present this action whereas the 14 lactams reported in this study exhibited an inhibition below 300 nM. It is possible that due to the electro-donating lactam nitrogen atom (vs. the O of the lactone), the electron density on the carbonyl group is sufficiently modified to strengthen a favourable hydrogen bond in the active site (see molecular modelling studies). Moreover, the fcompounds **48**, **49,** and **51** (entries 2, 3, 5) appear to be the best DYRK1A inhibitors of the series with more potent IC_50_ than the lactones **1a** and **1b** (entry 1).

**Table 4.** Measured IC_50_ values (nM) on Haspin, CLK1 and DYRK1A.

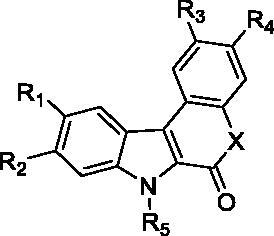						
Entry	Compound	Kinase inhibition (IC_50_)^a^ (IC_50_ in nM)			Selectivity index	
		Haspin	CLK1	DYRK1A	CLK1/Haspin	DYRK/Haspin
Ref	**1a**	ND	ND	74	ND	ND
Ref	**1b**	ND	ND	67	ND	ND
1	**46**	**7**	51	68	7	10
2	**48**	**6**	**17**	**19**	3	3
3	**49**	**1**	**13**	**15**	13	15
4	**50**	**7**	88	196	13	28
5	**51**	**4**	**10**	**41**	3	10
6	**52**	16	78	242	5	15
7	**54**	**4**	82	97	21	24
8	**55**	**2**	33	130	17	65
9	**56**	14	213	226	15	16
10	**57**	34	259	280	8	8
11	**59**	30	94	73	3	2
12	**60**	19	122	143	6	8
13	**61**	**5**	78	104	16	21
14	**62**	18	124	131	7	7

^a^IC_50_ values were determined on Haspin, CLK1 and DYRK1A when the residual kinase activity was ≤25% on Haspin at a compound concentration of 1 μM in Table 3. They were calculated from a dose-response curve for which each point was measured in duplicate, and reported in nM. Selectivity indexes (SI) were calculated as follows: IC_50_ DYRK1A or CLK1/IC_50_ Haspin.

**Table 5.** Effects on cell viability, EC_50_ on RPE1 and U-2 OS cell lines^a^.

Entry	Compound	RPE1 EC_50_ in µM	U-2 OS EC_50_ in µM
1	**46**	>25	>25
2	**48**	7.3	7
3	**49**	>25	>25
4	**50**	20.8	4.3
5	**51**	>25	12.6
6	**54**	>25	>25
7	**55**	10.7	3.4
8	**56**	>25	>25
9	**61**	19.1	8.7

^a^Cells were incubated with increasing doses of each compound (up to 50 µM) for 48 h. Cell viability was determined by MTS assay in triplicate and EC_50_ (µM) were calculated from the dose-response curves.

The concomitant action of DYRK1A inhibitors with CLK1 was confirmed since most of the Haspin inhibitors were also active on DYRK1A and CLK1. The mode of interaction of **49**, which is highly active on Haspin and exhibits strong activity on the other two kinases, was studied by molecular docking experiments (see Molecular docking studies section). Considering the two enzymes we can say that compounds **48** and **49** inhibit DYRK1A and CLK1 almost equally in the nanomolar range.

As regards to Haspin inhibition, the chemical series of type **VII** is of great interest. Eight compounds showed an IC_50_ below 10 nM. Compound **45** (Table 3, entry 1), without any substituent on the aromatic parts, presented no kinase activity. To maximise the inhibition, at least one methoxy OCH_3_ or hydroxy OH group is required in positions *C*-2 or *C*-3 (compounds **46, 48, 50, 51, 54, 55, 61**), the amino derivatives or amides being less effective (Table 4, entries 9 and 10).

Regarding the indole ring substituents, the absence of a substituent or the presence of an OCH_3_ or a fluorine in *C*-10 position did not significantly affect the Haspin inhibition (products **46, 51** and **61**, entries 1, 5, 13) when a functional group was also present on the *C*-2 or *C*-3 positions of the phenyl ring. Finally, the presence of ether in position *C*-9 (entry 11 vs. 5) or the methylation of indole (entry 14 vs. 5) made the compounds less effective.

The inhibitory activity of compound **49** on Haspin was about 12 times stronger than that of the other two enzymes. At 20 nM this molecule inhibited the 3 enzymes quantitatively. Compound **55** showed an even higher selectivity since the selectivity for CLK1 was identical, but against DYRK1A it rose to a factor of 65. At this stage, we can almost say that at a dose of 20 nM, this molecule shows a dual inhibition of Haspin and CLK1.

**Figure 3.**
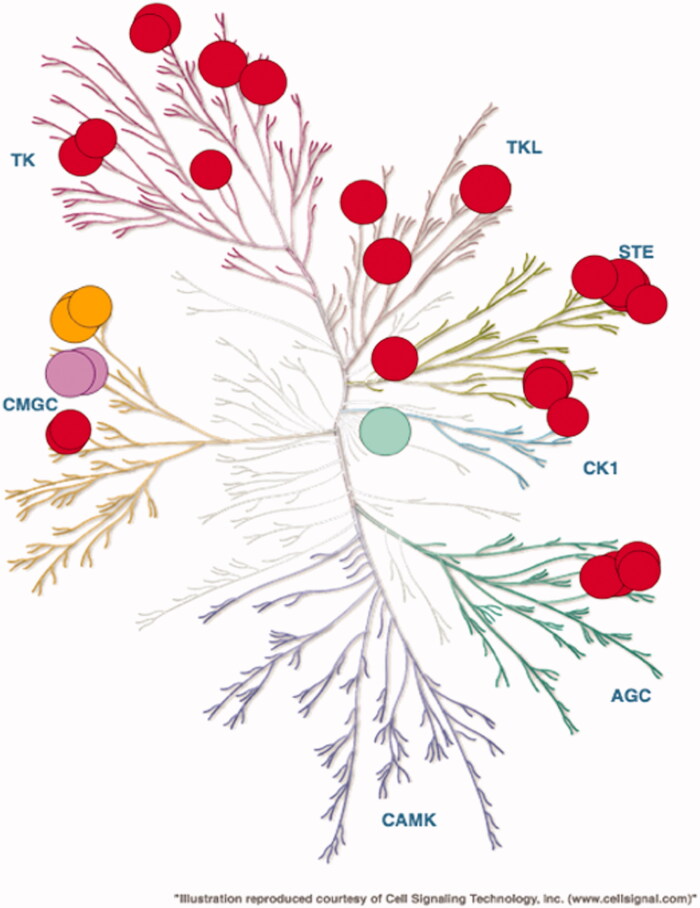
Selectivity profile of compound **55** evaluated on 486 kinases at 1 μM in duplicate (SelectScreen Whole Panel, Life Technologies) and represented on a dendrogram (courtesy of Cell Signalling Technology). Kinases whose activities are inhibited by 80% and above are shown as dots, Haspin is visualised as a green dot, CLKs as purple dots and DYRKs as orange dots.

We further evaluated the selectivity profile of compound **55** on a larger panel of kinases (SelectScreen Whole Panel, Life Technologies). The inhibition profile of **55,** evaluated at 1 μM, is depicted on a dendrogram on Figure 3 where kinases inhibited by a minimum of 80% are shown. A full list of the kinases tested is shown on Table S1 in supplementary information.

The inhibitory activities of compound **55** on Haspin, CLKs and DYRKs are well found in this new screening study. Product **55** is not specific but appears to be relatively selective since the compound **55** (AS-N14) presents a reasonable selectivity profile against a panel of 486 tested kinases since it inhibits 73 of the 486 kinases by >80%, and 159 of the 486 kinases by >50% at a dose of 1 µM.

### Molecular docking studies

**Figure 4.**
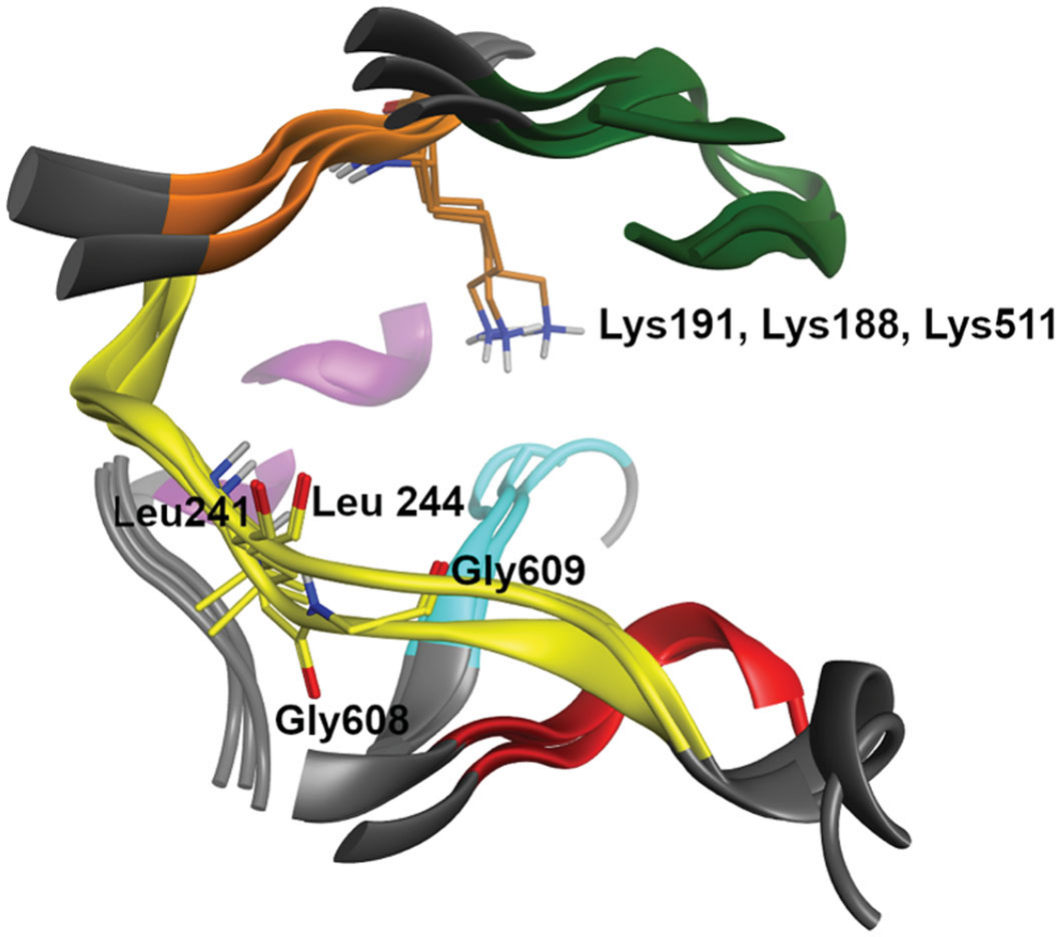
Superimposition of the ATP site of the three protein kinases, CLK1, DYRK1A and Haspin (residues and ribbons are coloured regarding their localisation in the kinase structure: hinge region (yellow), DFG motif (cyan), G loop (green), Catalytic K (orange), αC-helix (purple) and HRD region (red)), drawn with MOE software.^24^ Non-polar hydrogen atoms are hidden for clarity. Catalytic lysine and residues in the hinge region forming hydrogen bonds with the ligand are highlighted. CLK1: Leu244 and Lys191, DYRK1A: Leu241 and Lys188, and Haspin: Gly608, Gly609 and Lys 511.

Molecular docking studies were carried out using Glide^28–30^ from the Schrödinger Suite^31^, in order to compare putative binding modes and explore interactions within the active sites of CLK1, DYRK1A and Haspin kinases. Active sites of the three crystal structures were superimposed and are shown in Figure 4. The residues engaging hydrogen bond interactions with docked ligands are highlighted in stick form.

One of the most active compounds, **49** was docked in each active site of the three kinases. The docking poses exhibited the same putative binding mode, highlighting a hydrogen bond between the acceptor atom O of the lactam ring of **49** and the backbone of the hinge Leu244 (CLK1), Leu241 (DYRK1A) and Gly608 (Haspin). In addition, **49** formed another interaction with the hinge region of Haspin through a hydrogen bond between NH of the lactam ring and the backbone of Gly609 (Figure 4). In some docking poses, the molecule was flipped by 180° exposing the methoxy group of **49** towards the solvent area.

**Figure 5.**
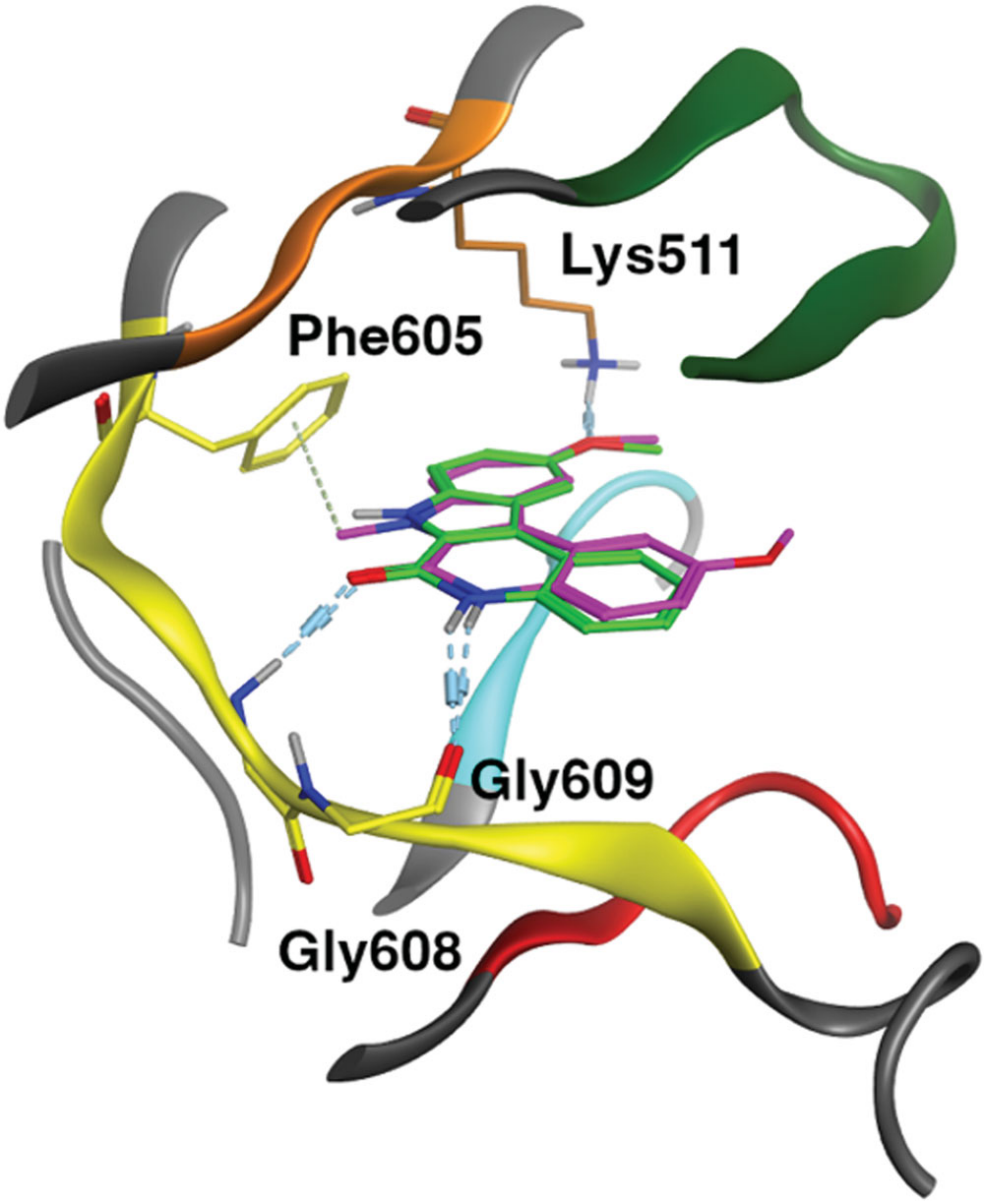
Binding mode representation of **49** (green) and **62** (purple) in ATP site of Haspin. Hydrogen bond interactions are represented in dashed lines.

Derivatives **51** and **62** were next docked in order to investigate the studied binding mode in greater depth since **62** has an *N*-methyl group on the pyrrole moiety. The best docking poses of the two compounds **51** and **62** were similar to **49** (Figure 5). No steric clash between the protein and the second methoxy group of the ligand was observed. Interestingly, **62** compared to **51** showed a weak H-pi interaction between the methyl group of the indole moiety and the gatekeeper, Phe605, of the kinase. The presence of the methyl group in **62** did not impact the binding mode of the compound, which explains the acceptable IC_50_ of **62.**

From the docking experiments, we predicted that the binding mode in each protein kinase, Haspin, CLK1 and DYRK1A, would be very similar. Nevertheless, as in most docking experiments, the docking score is not sufficient to predict the small variation in activity of the compounds and further intensive computational approaches such as free energy of binding (FEB) would be needed.

### Cell assays

**Figure 6.**
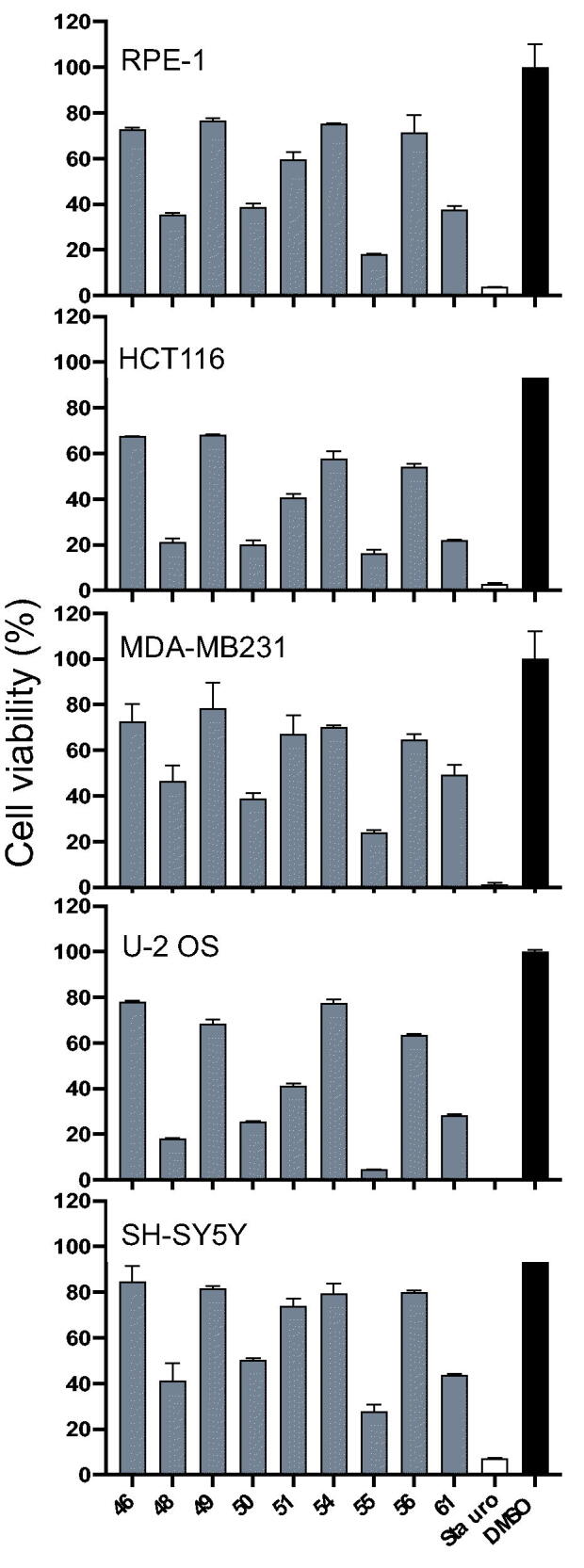
Effects of compounds on cell viability. Cell viability was assessed on the following human cell lines: U-2 OS (osteosarcoma), HCT116 (colorectal cancer), MDA-MB231 (breast cancer), SH-SY5Y (neuroblastoma) and RPE-1 (retinal fibroblast immortalised with hTERT). Cells were incubated with 25 μM of selected compounds or 10 μM of staurosporine or 0.1% of DMSO for 48 h. Cell viability was evaluated in triplicate via MST assay and results expressed as percentage of DMSO control (mean set at 100%). Results on graphs are mean ± SD.

We next analysed the effects of selected compounds (Haspin IC_50_ <15 nM) on the cell viability of several cell lines from osteosarcoma (U-2 OS), colorectal cancer (HCT116), breast cancer (MDA-MB231) and neuroblastoma (SH-SY5Y) as well as retinal fibroblast RPE-1 immortalised with hTERT (Figure 6). In a primary screen, all compounds were tested in triplicate at 25 μM and viability was expressed as percentage of a DMSO control. U-2 OS and HCT116 cell lines appeared to be the most sensitive to our compounds and were even slightly more affected than the non-cancerous RPE-1 cell line, whereas the SH-SY5Y and MDA-MB231 cell lines emerged as the most resistant ones. Compounds **46**, **49**, **51**, **54,** and **56** had little to no effect on all the tested cell lines. This can be explained by the low solubility of the compounds, or their low affinity for lipidic plasma membrane or a high metabolisation rate in aqueous solution/cellular environment. On the other hand, several compounds such as **48**, **61**, **55,** and **50** showed a reduction of equal or more than 75% of cell viability compared to the DMSO control.

Hence, dose-response experiments were carried out on both U-2 OS and RPE-1 cell lines and EC_50_ were calculated (Table 5). The results confirmed the lack of efficacy of compounds **46**, **49**, **54,** and **56** which showed EC_50_ >25 μM on both cell lines regardless of their activity on Haspin kinase (IC_50_ of 7, 1, 4 and 14 nM respectively). Derivative **51**, despite no effect observed on RPE-1 cells (EC_50_ >25 μM), showed a moderate activity on cell viability of the U-2 OS line with an EC_50_ of 12.6 μM. The compounds were generally more efficient at inhibiting the viability of U-2 OS cancerous cells than that of normal RPE-1. Amongst the selected compounds, **55** and **50** displayed the strongest effect on the viability of U-2 OS cells (EC_50_ of 3.4 and 4.3 μM, respectively). They were between 2 and 5 times more active on U-2 OS compared to RPE-1 cell viability.

Taken together, these results showed that some of our compounds such as **55** and **50** displayed interesting effects on cell viability of several cancerous cell lines.

**Figure 7.**
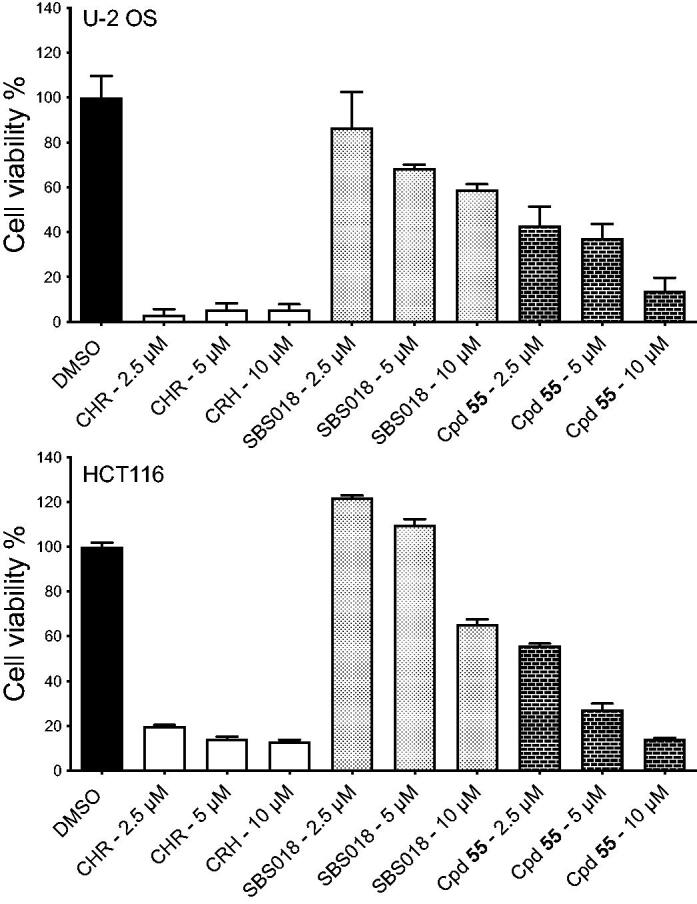
Effects of compounds on U-2 OS and HCT116 spheroid viability. Cell viability in spheroids from U-2 OS and HCT116 cells was measured after 7 days of treatment with DMSO (0.5%), CHR6494 (CHR), SBS018 or Cpd**55** at a single dose of 2.5, 5, and 10 µM, on day 0. Cell viability is expressed in percentage of the DMSO control. *n* = 3, results are mean ± s.e.m.

We further examined the effect of our most efficient compound (**55**) on cancerous cells growing in 3D spheroids. U-2 OS and HCT116 cells spheroids were prepared and treated with different concentrations of either compound **55**, CHR-6494 or SBS018 (compound **21** in ref Elie et al)^26^ or with 0.5% DMSO for 7 days, after which, spheroids viability was evaluated (Figure 7). We observed a marked dose-dependent effect of compound **55** on both U-2 OS and HCT116 spheroid cell viability after 7 days of treatment. This effect was milder than the one observed with CHR-6494 and stronger than the one induced by SBS018 at similar concentrations.

**Figure 8.**
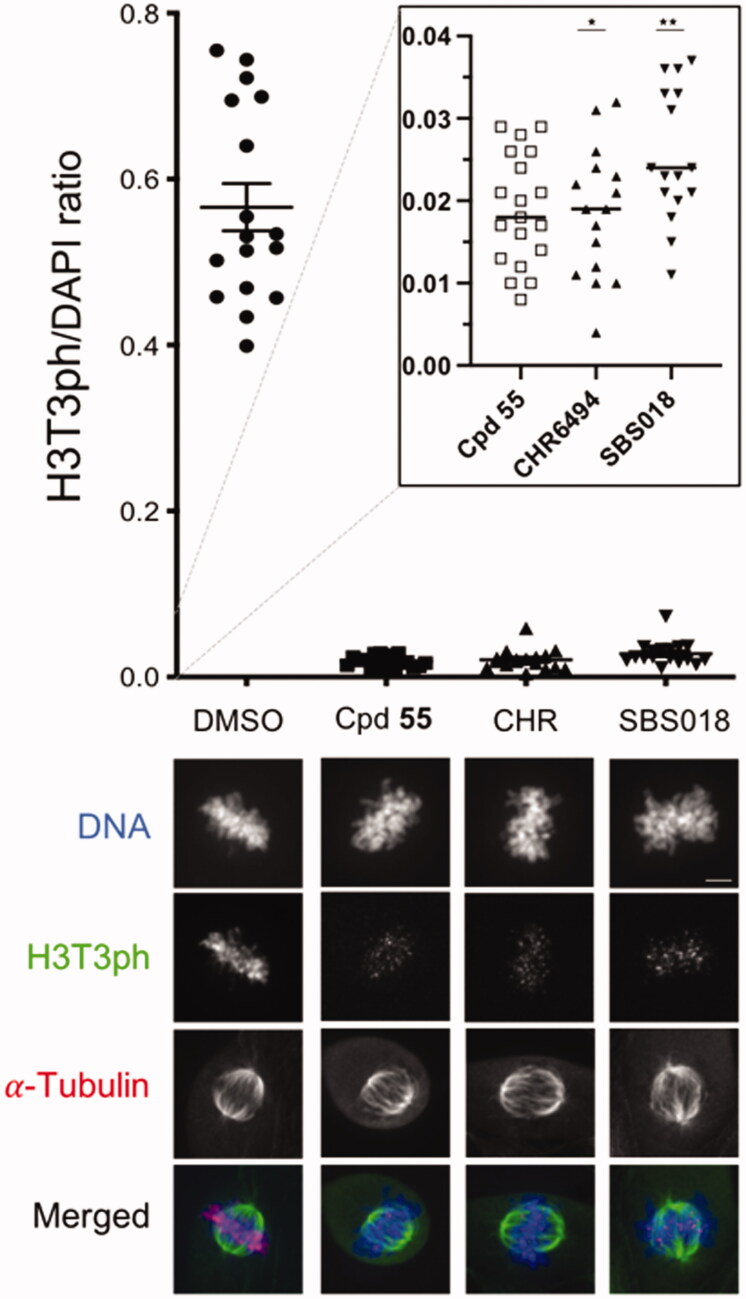
Cellular endogenous Haspin inhibition. U-2 OS cells were treated for 16 h with 0.5 µM of each compound or 0.1% of DMSO (CHR: CHR-6494). Haspin activity was monitored by immunofluorescence staining of phosphorylated Histone H3 on threonine 3 (H3T3ph, green); α-Tubulin was visualised in red and DNA was stained by DAPI (blue). Haspin activity was quantified in prometaphase/metaphase cells measuring the H3T3ph and DAPI signals and representing the H3T3ph/DAPI ratio on a scatter plot (upper panel). The inserted dot plot allows the comparison of the 3 tested compounds on a more precise scale; *n* ≥ 15, *p ≤ 0.05; **p ≤ 0.01 (two-tailed unpaired t-test). Representative images are presented on the lower panel, Bar 5 µm.

We further characterised the functional effects of our most efficient compound **55** on endogenous Haspin in U-2 OS cells by immunofluorescence, quantifying the Haspin specific H3T3ph signal in early mitotic cells. The H3T3ph signal was measured on cells treated with 0.5 µM of compound **55** or CHR-6494 or SBS018 or with 0.1% of DMSO for 16 h (Figure 8). Our results showed that compound **55** could inhibit intracellular Haspin with a very similar efficiency to that off CHR-6494 or SBS018. These results further validate the functionality of our compound in cells, on the endogenous Haspin kinase activity.

**Figure 9.**
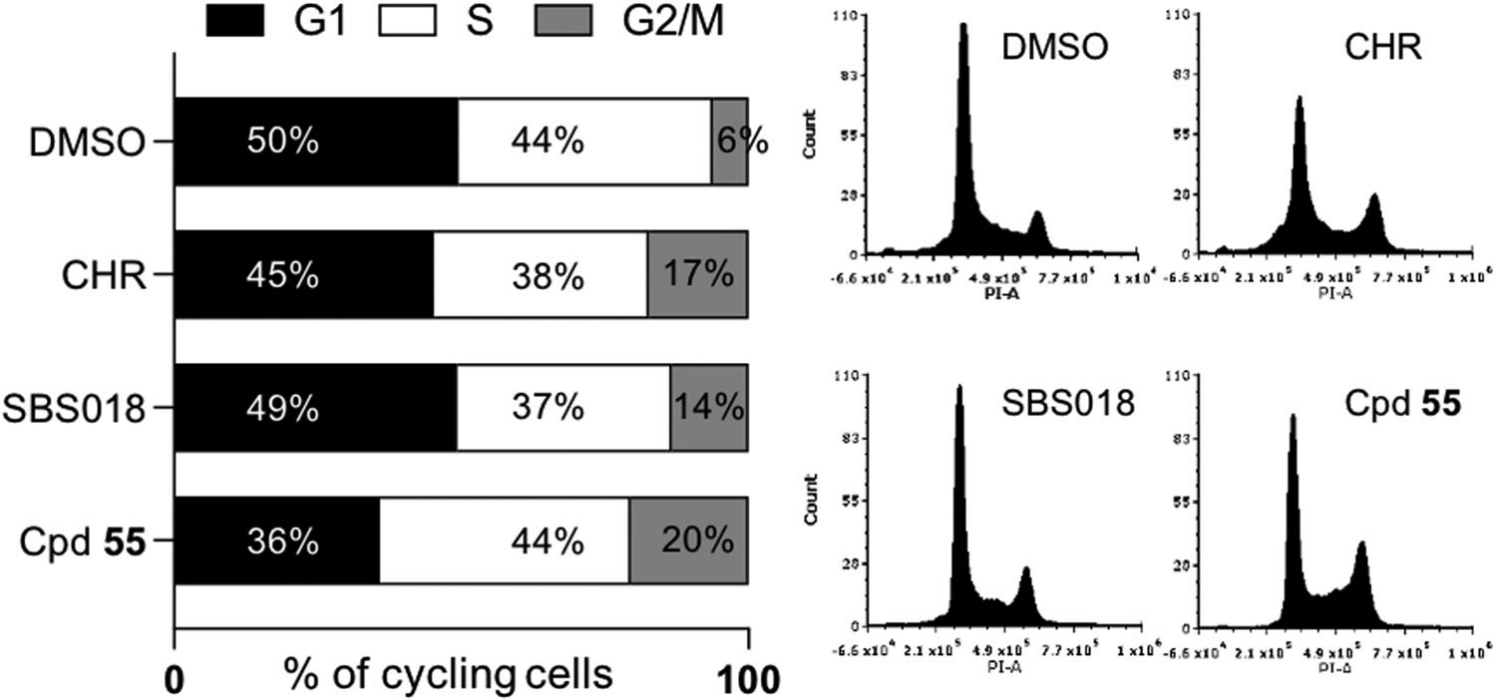
Effect of compounds on the cell cycle. U-2 OS cells were treated for 24 h with 1 µM of each compound or 0.2% DMSO (CHR: CHR-6494). DNA content was measured by flow cytometry and the percentages of cells in each phase of the cell cycle is represented on a proportional bar graph. Representative profiles for each treatment are shown on the right panels.

We then characterised the effect of compound **55** on the cell cycle on U-2 OS cells. Cells were treated for 24 h with 1 µM of compound 55, CHR-6494, SBS018 or DMSO at 0.2% and their cell cycle profile was analysed by flow cytometry (Figure 9). Analysis of flow cytometry profiles showed, as expected, a strong increase in the percentage of cells in G2/M phase of the cell cycle with compound **55** as well as with the two references CHR-6494 and SBS018 compared to the DMSO control (20, 17, and 14%, respectively vs. 6% for the DMSO control). Concomitantly, compound **55** further induced a reduction of cells in the G1 phase compared to the control (36 vs. 50%, respectively), an expected result of the strong observed G2/M delay. These results are consistent with an impaired Haspin function inducing prolonged mitoses as previously described (Huertas et al. 2012; Peiling Wang et al. 2021/PMID:34551143).

## Conclusion

We have synthesised a series of new Lamellarin analogues using the indolo[2,3-*c*]quinolone-6-one core. The analogues were obtained after a sequence involving (i) a palladium catalysed cross coupling reaction between 2-indolic esters and 2-nitrophenyl boronic acids as building blocks, and (ii) a cyclic lactam formation involving a reduction and an annelation. Twenty-two novel derivatives were synthesised and evaluated for their inhibitory activity on Haspin kinase and on a panel of 7 other protein kinases for selectivity assessment. Among this series, 8 compounds inhibited Haspin kinase with IC_50_ below 10 nM. Docking studies showed a double hydrogen bond between the lactam and the hinge region of the kinase. The most active compounds **49** and **55** possess IC_50_ of 1 and 2 nM respectively with selectivity towards the parent kinases DYRK1A and CLK1 between a 13 and 65-fold factor. Furthermore, the most selective compound **55** exerted an interesting cellular effect on the osteosarcoma U-2 OS cell line as well as on U-2 OS and colorectal carcinoma HTC116 spheroid viability. Additionally, we further validated the functionality of compound **55** on endogenous Haspin activity in cells. This interesting Haspin inhibitor will be used in further studies to develop efficient and selective Haspin inhibitors.

### Experimental section

#### Chemistry

All reagents and solvents were purchased from commercial sources and used without further purification.^1^H NMR and ^13 ^C NMR spectra were recorded on 400 MHz and/or 500 MHz Bruker FT-NMR spectrometers. All chemical shifts are given as δ values (ppm) with reference to tetramethylsilane (TMS = 0) as an internal standard. The peak patterns are indicated as follows: s, singlet; d, doublet; t, triplet; m, multiplet; q, quartette. The coupling constants, *J*, are reported in Hertz (Hz). UV detection at 210 nm. High resolution mass (MS) analysis was conducted using an LC/MSD TOF spectrometer system with electrospray ionisation (ESI). Reactions were monitored via thin-layer chromatography (TLC) carried out on commercial silica gel plates (GF254) under UV light. Column chromatography was performed on silica gel 60 (200–300 mesh).

#### General procedure A: preparation of 3-iodoindoles (11–13)

Indole-2-carboxylic acid derivatives (3.1 mmol) were dissolved in Ethanol (50 ml) and conc. sulphuric acid (5 ml) was added. The solution was refluxed for about 12 h (monitoring with TLC) after completion of the reaction. The solution was poured into cold water (150 ml), and the white solid precipitate formed was collected by filtration. The solid material was washed with water and dried to produce quantitatively derivatives **6–9**. Crude materials were next dissolved in a mixture containing crushed KOH (4.0 equiv.) pellets in DMF (15 ml) at room temperature. Next Iodine (1.5 equiv.) dissolved in DMF (3 ml) was added dropwise and the mixture stirred for 4 h at room temperature (monitored with TLC). After completion (TLC monitoring), the reaction mixture was poured onto a saturated aqueous solution of NaHSO_3_ (15 ml), NH_4_OH (30%, 2 ml) and water (15 ml). The solid was filtered, dried under reduced pressure, and used directly without further purification. All spectral data for **10**, **11**, **13** are in agreement with previous reports [20, 21]. Compounds **11–13** were engaged immediately in the next step.

#### Ethyl 3-iodo-6-methoxy-1*H-*indole-2-carboxylate (12)

Compound **12** was isolated as a white solid (81%) starting from **8** following the general procedure A. ^1^H NMR (CDCl_3_, 400 MHz) 1.23 (*t*, *J =* 7.2 Hz, 3H), 4.25 (*q*, *J =* 7.2 Hz, 2H), 3.99 (*s*, 3H), 7.05 (*s*, 1H), 7.20 (d, *J =* 7.4 Hz, 1H), 8.10 (d, *J* = 7.4 Hz, 1H), 11.55 (*s*, 1H). ^13 ^C NMR (CDCl_3,_ 125 MHz) 14.4, 55.6, 61.3, 66.4, 93.5, 113.2, 124.3, 124.5, 126.1, 137.1, 159.8, 161.0; ESI-MS *m*/*z* 368 [M + Na]^+^. IR (KBr) *ν* 3308, 2983, 1674,1600,1410, 754 cm^−1^.

#### Ethyl 3-iodo-5-methoxy-1-methyl-1*H-*indole-2-carboxylate (14)

Compound **11** (1.0 g, 2.9 mmol) was added to a stirred suspension of oil-free sodium hydride NaH (0.1 g, 4.36 mmol) in DMF (10 ml) at 0 °C and the mixture was stirred for 10 min at this temperature. Then methyl iodide (0.49 g, 3.48 mmol) was added at 0 °C and the whole mixture was stirred at room temperature (20 °C) for 1 h. The solution was poured into ice cold water (50 ml) and extracted with CH_2_Cl_2_ (2 × 25 ml). The organic layers were dried with sodium sulphate and concentrated under reduced pressure to give **14** in a quantitative yield (1.0 g). ^1^H NMR (CDCl_3_, 400 MHz) 1.24 (*t*, *J =* 7.2 Hz, 3H), 3.95 (*s*, 3H), 3.81 (*s*, 3H), 4.28 (*q*, *J =* 7.2 Hz, 2H), 7.23 (d, *J =* 7.4 Hz, 1H), 7.26 (d, *J* = 7.4 Hz, 1H), 7.64 (s, 1H). ^13 ^C NMR (CDCl_3,_ 125 MHz) 14.6, 33.6, 55.9, 61.5, 66.5, 103.2, 113.2, 117.9, 129.2, 130.4, 134.5, 155.6, 161.1; ESI-MS *m*/*z* 360 [M + H]^+^; IR (KBr) *ν* 2995, 2826, 1701, 1573–1454, 767 cm^−1^.

#### General Procedure B: synthesis of 2-nitrophenyl boronic acids (15–20)

A dry nitrogen-flushed 25-ml round-bottomed flask equipped with a magnetic stirrer and a septum was loaded with aryl iodide (4.0 mmol, 1.0 equiv.). Dry THF (6 ml) was added, and the resulting solution was cooled to −78 °C. To the resulting cooled mixture was added dropwise 2.2 ml of a 2 M solution of PhMgCl (4.8 mmol, 1.2 equiv.) in THF. After 5 min, 0.536 ml of trimethyl borate (4.8 mmol, 1.2 equiv.) was added dropwise to the reaction solution. The reaction mixture was stirred for 30 min at −78 °C, warmed to −10 °C and quenched with 4 ml of a 2 M aqueous solution of HCl. The resulting mixture was extracted with Et_2_O (20 ml) and washed with H_2_O (10 ml) and brine (10 ml). The resulting organic layer was dried over Na_2_SO_4_, filtered, and concentrated under reduced pressure. The crude boronic acids were used in subsequent transformations without additional purification.

#### General Procedure C: synthesis of 2-nitrophenyl boronic esters (21–24)

In a 250-ml round-bottomed flask purged and maintained with an inert atmosphere of nitrogen was placed a solution of substituted-1-bromo-2-nitrobenzene (1.0 equiv.) in 1,4-dioxane (150 ml). Bis(pinacolato)diboron (1.5 equiv.), Pd(dppf)Cl_2_ (0.03 equiv.), and potassium acetate (2.0 equiv.) were then added under vigorous stirring and placed in a heated bath at 80 °C. The resulting solution was stirred overnight, cooled to room temperature and concentrated under reduced pressure. The residue was used for further steps without purification.

#### General procedure D: synthesis of 3-arylated indoles (25–44)

To a mixture of ethyl 3-iodo-5- or 6-substituted-1*H*-Indole-2-carboxylate **10–14** (1.0 equiv.), crude substituted-2-nitroarylboronic acid **15–20** or 4,4,5,5-tetramethyl-2-(substited-2-nitrophenyl)-1,3,2-dioxaborolane **21–24** (2.0 equiv.) and finally sodium bicarbonate (3.0 equiv.) was added a 10:1 v/v mixture of 1,4-dioxane and water. The reaction mixture was degassed with argon for about 30 min and Pd(PPh_3_)_4_ (10.0 mol %) was added in one portion. The resulting mixture was heated to 100 °C. After 12 h, the mixture was cooled to room temperature and diluted with cold water. The mixture was extracted with EtOAc. The combined organic layers were washed with brine, dried over Na_2_SO_4_, filtered and concentrated *in vacuo*. The crude product was finally purified by silica gel flash chromatography to afford the desired product **25–44**.

#### Ethyl 3–(2-nitrophenyl)-1*H-*indole-2-carboxylate (25)

The reaction was carried out as described in general procedure D using **4** (0.100 g, 0.39 mmol, 1.0 equiv.), 1-naphthaleneboronic acid (0.083 g, 0.47 mmol, 1.2 equiv.). Purification by silica gel flash chromatography (EtOAc) afforded **25** (0.083 g, 70%) as a white solid. R*_f_* (EtOAc): 0.17. m.p. 144–146 °C. ^1^H NMR (CDCl_3_, 400 MHz) 1.13 (*t*, *J =* 7.2 Hz, 3H), 4.19 (*q*, *J =* 7.2 Hz, 2H), 7.16 (*t*, *J =* 7.2 Hz, 7.6 Hz, 1H), 7.37 (*t*, *J =* 8.0 Hz, 7.2 Hz, 1H), 7.42–7.48 (*m*, 2H), 7.51–7.56 (*m*, 2H), 7.67 (*t*, *J =* 7.2 Hz, 7.6 Hz, 1H), 8.10 (d, *J =* 8.0 Hz, 1H), 9.14 (*s*, 1H). ^13 ^C NMR (DMSO-d_6,_ 125 MHz) 13.7, 61.2, 112.0, 118.7, 120.8, 121.3, 123.1, 124.4, 126.0, 127.5, 128.3, 129.2, 132.2, 133.2, 135.7, 150.0, 161.3. ESI-MS *m*/*z* 311 [M + H]^+^. IR (KBr) *ν* 3320, 3001, 2983, 2938, 1693, 1623, 1525, 1433, 1291, 1205, 1117, 784 cm^−1^.

#### Ethyl 3–(5-methoxy-2-nitrophenyl)-1*H-*indole-2-carboxylate (26)

The reaction was carried out as described in general procedure D using **10** (0.100 g, 0.317 mmol, 1.0 equiv.) and (5-methoxy-2-nitrophenyl)boronic acid **17** (0.124 g, 0.634 mmol, 2.0 equiv.). Purification by flash chromatography on silica gel (eluent 9/1 PE/EtOAc) afforded **26** (0.072 g, 67%) as a yellow solid. R_f_ = 0.4 (PE/EtOAc 8/2). ^1^H NMR (CDCl_3_, 400 MHz) 1.18 (*t*, *J =* 7.2 Hz, 3H), 3.92 (*s*, 3H), 4.19–4.24 (*m*, *J =* 7.2 Hz, 2H), 6.98 (d, *J =* 2.4 Hz, 1H), 7.02 (dd, *J =* 2.4 Hz, 8.8 Hz, 1H), 7.19 (*t*, *J =* 7.6 Hz, 1H), 7.40 (*t*, *J =* 7.6 Hz, 1H), 7.50 (*m*, 2H), 8.20 (d, *J =* 8.8 Hz, 1H), 9.11 (*s*, 1H); ^13 ^C NMR (DMSO-d_6,_ 125 MHz) 14.4, 55.8, 60.5, 111.9, 112.9, 113.4, 116.9, 117.9, 118.4, 120.6, 121.3, 124.6, 125.1, 127.6, 136.5, 151.6; 153.0, 159.8, 162.0. ESI-MS *m*/*z* 341 [M + H]^+^. IR (KBr) *ν* 3260, 3050, 2958, 1710, 1612, 1507, 1463,1291,1167, 1048, 984, 748 cm^−1^.

#### Ethyl 3–(4-hydroxy-2-nitrophenyl)-1*H-*indole-2-carboxylate (27)

The reaction was carried out as described in general procedure using **10** (0.100 g, 0.317 mmol, 1.0 equiv.) and 3-nitro-4–(4,4,5,5-tetramethyl-1,3,2-dioxaborolan-2-yl)phenol **21** (0.168 g, 0.634 mmol, 2.0 equiv.). Purification by flash chromatography on silica gel (eluent 8.5/1.5 PE/EtOAc) afforded **27** (0.052 g, 51%) as a yellow solid. R_f_ = 0.3 (PE/EtOAc 8/2). ^1^H NMR (CDCl_3_, 400 MHz) 1.10 (*t*, *J =* 6.8 Hz, 3H), 4.14 (*q*, *J =* 6.8 Hz, 2H), 7.08 (*t*, *J =* 7.6 Hz, 1H), 7.18 (d, *J =* 7.6 Hz, 1H), 7.30–7.37 (*m*, 3H), 7.46 (*s*, 1H), 7.51 (d, *J =* 10.5 Hz, 1H), 10.47 (*s*, 1H), 11.98 (*s*, 1H). ^13 ^C NMR (DMSO-d_6,_ 125 MHz) 14.2, 60.9, 111.1, 113.2, 118.3, 119.4, 120.4, 120.6, 121.0, 123.4, 125.6, 127.4, 134.6, 136.8, 150.5, 157.7, 161.5. ESI-MS *m*/*z* 327 [M + H]^+^. IR (KBr) *ν* 3425, 3214, 3049, 2932, 1692, 1620, 1512, 1117, 1026, 984 cm^−1^.

#### Ethyl 3–(5-hydroxy-2-nitrophenyl)-1*H-*indole-2-carboxylate (28)

The reaction was carried out as described in general procedure C using **10** (0.100 g, 0.317 mmol, 1.0 equiv.) and 4-nitro-3–(4,4,5,5-tetramethyl-1,3,2-dioxaborolan-2-yl)phenol **22** (0.168 g, 0.634 mmol, 2.0 equiv.). Purification by flash chromatography on silica gel (eluent 8.5/1.5 PE/EtOAc) afforded **28** (0.058 g, 55%) as a yellow solid. R_f_ = 0.3 (PE/EtOAc 8/2). ^1^H NMR (CDCl_3_, 400 MHz) 1.01 (*t*, *J =* 7.2 Hz, 3H), 4.11 (*q*, *J =* 7.2 Hz, 2H), 6.81 (*s*, 1H), 6.95–6.99 (*m*, 1H), 7.08–7.11 (*m*, 1H), 7.29–7.34 (*m*, 2H), 7.51 (d, *J =* 8.4, 1H), 8.07 (d, *J =* 8.8, 1H), 10.89 (*s*, 1H, br), 12.04 (*s*, 1H). ^13 ^C NMR (DMSO-d_6,_ 125 MHz) 14.1, 60.9, 111.8, 113.2, 115.2, 118.7, 119.7, 121.2, 122.8, 123.2, 123.7, 125.6, 127.8, 132.3, 136.8, 141.7 161.9. ESI-MS *m*/*z* 327 [M + H]^+^. IR (KBr) *ν* 3359, 3245, 3055, 2986, 2939, 1689, 1622, 1518, 1248,1110,1060, 975,734 cm^−1^.

#### Ethyl 5-methoxy-3–(2-nitrophenyl)-1*H-*indole-2-carboxylate (29)

The reaction was carried out as described in general procedure C using **11** (0.100 g, 0.290 mmol, 1.0 equiv.) and (2-nitrophenyl)boronic acid **15** (0.115 g, 0.579 mmol, 2.0 equiv.) and NaHCO_3_ (0.073 g, 0.870 mmol, 3.0 equiv.). Purification by flash chromatography on silica gel (eluent 9/1 PE/EtOAc) afforded **29** (0.055 g, 56%) as a yellow solid. R_f_ = 0.4 (PE/EtOAc 8/2). ^1^H NMR (CDCl_3_, 400 MHz) 1.12 (*t*, *J =* 7.2 Hz, 3H), 3.76 (*s*, 3H), 4.17 (*q*, *J =* 7.2 Hz, 2H), 6.76 (d, *J =* 2.4 Hz, 1H), 7.04 (dd, *J =* 2.4 Hz, 8.8 Hz, 1H), 7.35 (d, *J =* 9.2 Hz, 1H), 7.52–7.56 (*m*, 2H), 7.67 (*t*, *J =* 7.2 Hz, 1H), 8.08 (d, *J =* 8.0 Hz, 1H), 9.01 (*s*, 1H). ^13 ^C NMR (DMSO-d_6,_ 125 MHz) 14.1, 55.7, 60.9, 100.1, 114.4, 117.4, 117.5, 123.7, 124.7, 127.3, 129.0, 129.4, 132.1, 133.3, 133.7, 150.2, 155.0, 161.3. ESI-MS *m*/*z* 341 [M + H]^+^. IR (KBr) *ν* 3245, 3060, 2974, 2918, 1698, 1608, 1520, 1404, 1117, 1023, 984, 735 cm^−1^.

#### Ethyl 5-methoxy-3–(4-methoxy-2-nitrophenyl)-1*H-*indole-2-carboxylate (30)

The reaction was carried out as described in general procedure C using **11** (0.100 g, 0.290 mmol, 1.0 equiv.) and (4-methoxy-2-nitrophenyl)boronic acid **16** (0.113 g, 0.579 mmol, 2.0 equiv.). Purification by flash chromatography on silica gel (eluent 8/2 PE/EtOAc) afforded **30** (0.043 g, 40%) as a yellow solid. R_f_ = 0.35 (PE/EtOAc 8/2). ^1^H NMR (CDCl_3_, 400 MHz) 1.17 (*t*, *J =* 7.2 Hz, 3H), 3.76 (*s*, 3H), 3.94 (*s*, 3H), 4–19 (*q*, *J =* 7.2 Hz, 2H), 6.77 (d, *J =* 2.4 Hz, 1H), 7.03 (dd, *J =* 2.4 Hz, 8.8 Hz, 1H), 7.21 (dd, *J =* 2.8 Hz, 8.4 Hz, 1H), 7.34 (d, *J =* 8.8 Hz, 1H), 7.42 (d, *J =* 8.4 Hz, 1H), 7.63 (d, *J =* 2.8 Hz, 1H), 9.01 (*s*, 1H). ^13 ^C NMR (DMSO-d_6_, 125 MHz) 14.1, 55.7, 56.5, 60.9, 100.1, 109.8, 114.4, 117.3, 119.3, 121.2, 123.7, 123.8, 127.6, 132.1, 134.6, 150.8, 155.0, 159.2, 161.4. ESI-MS *m*/*z* 371 [M + H]^+^. IR (KBr) *ν* 3254, 3030, 2934, 1703, 1613, 1556, 1403, 1209, 1017, 980, 756 cm^−1^.

#### Ethyl 5-methoxy-3–(5-methoxy-2-nitrophenyl)-1*H-*indole-2-carboxylate (31)

The reaction was carried out as described in general procedure C using **11** (0.100 g, 0.290 mmol, 1.0 equiv.) and (5-methoxy-2-nitrophenyl)boronic acid **17** (0.113 g, 0.579 mmol, 2.0 equiv.). Purification by flash chromatography on silica gel (eluent 8/2 PE/EtOAc) afforded **31** (0.053 g, 49%) as a yellow solid. R_f_ = 0.35 (PE/EtOAc 8/2). ^1^H NMR (CDCl_3_, 400 MHz) 1.19 (*t*, *J =* 7.2 Hz, 3H), 3.79 (*s*, 3H), 3.97 (*s*, 3H), 4.22 (*q*, *J =* 7.2 Hz, 2H), 6.80 (d, *J =* 2.4 Hz, 1H), 7.05 (dd, *J =* 2.4 Hz, 8.4 Hz, 1H), 7.24 (dd, *J =* 2.4 Hz, 8.8 Hz, 1H), 7.36 (d, *J =* 8.8 Hz, 1H), 7.45 (d, *J =* 8.4 Hz, 1H), 7.65 (d, *J =* 2.4 Hz, 1H), 9.12 (*s*, 1H). ^13 ^C NMR (DMSO-d_6_,125 MHz) 14.1, 55.7, 56.8, 60.9, 100.2, 114.0, 114.5, 116.7, 117.3, 118.3, 123.6, 127.2, 127.8, 132.3, 137.5, 140.1, 155.0, 161.3, 163.8. ESI-MS *m*/*z* 371 [M + H]^+^. IR (KBr) *ν* 3320, 3001, 2983, 2938, 1673, 1623, 1525, 1433, 1291, 1117, 984 cm^−1^.

#### Ethyl 3–(4,5-dimethoxy-2-nitrophenyl)-5-methoxy-1*H-*indole-2-carboxylate (32)

The reaction was carried out as described in general procedure C using **11** (0.100 g, 0.290 mmol, 1.0 equiv.) and (4,5-dimethoxy-2-nitrophenyl)boronic acid **18** (0.131 g, 0.579 mmol, 2.0 equiv.). Purification by flash chromatography on silica gel (eluent 8/2 PE/EtOAc) afforded **32** (0.064 g, 55%) as a yellow solid, which was used in the next step. ^1^H NMR (CDCl_3_, 400 MHz) 1.17 (*t*, *J =* 7.2 Hz, 3H), 3.77 (*s*, 3H), 3.93 (*s*, 3H), 4.03 (*s*, 3H), 4.20 (*q*, *J =* 7.2 Hz, 2H), 6.77 (d, *J =* 2.0 Hz, 1H), 6.90 (*s*, 1H), 7.05 (dd, *J =* 2.0 Hz, 8.8 Hz, 1H), 7.36 (d, *J =* 8.8 Hz, 1H), 7.75 (*s*, 1H), 8.95 (*s*, 1H). ESI-MS *m*/*z* 401 [M + H]^+^. IR (KBr) *ν* 307 0, 2980,2930, 1705, 1633, 1525, 1433,1291,1132, 1045, 984 cm^−1^.

#### Ethyl 5-methoxy-3–(2-nitro-5-(trifluoromethyl)phenyl)-1*H-*indole-2-carboxylate (33)

The reaction was carried out as described in general procedure C using **11** (0.100 g, 0.290 mmol, 1.0 equiv.) and (2-nitro-5-(trifluoromethyl)phenyl)boronic acid **19** (0.136 g, 0.579 mmol, 2.0 equiv.). Purification by flash chromatography on silica gel (eluent 8/2 PE/EtOAc) afforded **33** (0.055 g, 47%) as a yellow solid. ^1^H NMR (CDCl_3_, 400 MHz) 1.17 (*t*, *J =* 7.2 Hz, 3H), 3.80 (*s*, 3H), 4.21–4.26 (*m*, *J =* 7.2 Hz, 2H), 6.76 (d, *J =* 2.0 Hz, 1H), 7.09 (dd, *J =* 2.0 Hz, 8.8 Hz, 1H), 7.39 (d, *J =* 8.8 Hz, 1H), 7.84 (d, *J =* 8.4 Hz, 1H), 7.87 (*s*, 1H), 8.19 (d, *J =* 8.4 Hz, 1H), 9.17 (*s*, 1H). ^13 ^C NMR (DMSO-d_6,_ 125 MHz) 14.0, 55.7, 61.1, 99.9, 114.5, 115.4, 117.5, 122.3, 124.3, 125.8, 126.3, 127.1, 130.4, 131.0, 132.0, 132.8, 152.4, 155.2, 161.1. ESI-MS *m*/*z* 409 [M + H]^+^. IR (KBr) *ν* 3290, 3071, 2929, 1685, 1622, 1200, 1098, 978, 756 cm^−1^.

#### Ethyl 3–(4-hydroxy-2-nitrophenyl)-5-methoxy-1*H-*indole-2-carboxylate (34)

The reaction was carried out as described in general procedure C using **11** (0.100 g, 0.290 mmol, 1.0 equiv.) and 3-nitro-4–(4,4,5,5-tetramethyl-1,3,2-dioxaborolan-2-yl)phenol **21** (0.153 g, 0.579 mmol, 2.0 equiv.) and NaHCO_3_ (0.073 g, 0.870 mmol, 3.0 equiv.). Purification by flash chromatography on silica gel (eluent 8/2 PE/EtOAc) afforded **34** (0.046 g, 45%) as a yellow solid, which was used in the next step. R_f_ = 0.3 (PE/EtOAc 8/2). ^1^H NMR (CDCl_3_, 400 MHz) 1.10 (*t*, *J =* 6.8 Hz, 3H), 3.68 (s, 3H), 4.10 (*q*, *J =* 6.8 Hz, 2H), 6.68 (d, *J =* 2.4 Hz, 1H), 6.98 (dd, *J =* 2.4 Hz, 8.8 Hz, 1H), 7.18 (dd, *J =* 2.4 Hz, 8.4 Hz, 1H), 7.36–7.42 (*m*, 2H), 7.44 (d, *J =* 2.4 Hz, 1H), 10.45 (*s*, 1H), 11.86 (*s*, 1H). ESI-MS *m*/*z* 357 [M + H]^+^; IR (KBr**)** *ν* 3405, 3344, 3074, 2966, 2918, 1690, 1623, 1 505, 1433, 1210, 1117, 1054, 764 cm^−1^.

#### Ethyl 3–(5-hydroxy-2-nitrophenyl)-5-methoxy-1*H-*indole-2-carboxylate (35)

The reaction was carried out as described in general procedure C using **11** (0.100 g, 0.290 mmol, 1.0 equiv.) and 4-nitro-3–(4,4,5,5-tetramethyl-1,3,2-dioxaborolan-2-yl)phenol **22** (0.153 g, 0.579 mmol, 2.0 equiv.). Purification by flash chromatography on silica gel (eluent 8/2 PE/EtOAc) afforded **35** (0.044 g, 43%) as a yellow solid. R_f_ = 0.3 (PE/EtOAc 8/2). ^1^H NMR (CDCl_3_, 400 MHz) 1.09 (*t*, *J =* 7.2 Hz, 3H), 3.69 (*s*, 3H), 4.10 (*q*, *J =* 7.2 Hz, 2H), 6.70 (d, *J =* 2.0 Hz, 1H), 6.81 (d, *J =* 2.8 Hz, 1H), 6.94–7.00 (*m*, 2H), 7.42 (d, *J =* 8.8 Hz, 1H), 8.07 (d, *J =* 8.8 Hz, 1H), 10.83 (*s*, br, 1H), 11.93 (*s*, 1H). ^13 ^C NMR (DMSO-d_6,_ 125 MHz) 14.1, 55.7, 60.8, 100.2, 114.3, 115.1, 117.2, 118.2, 119.6, 123.5, 127.1, 127.8, 132.1, 132.5, 141.8, 154.9, 161.3, 161.9. ESI-MS *m*/*z* 357 [M + H]^+^. IR (KBr) *ν* 3438, 3247, 3060, 2970, 2920, 1694, 1620, 1535, 1420, 1249, 1145, 1057, 984,734 cm^−1^.

#### Ethyl 3–(5-amino-2-nitrophenyl)-5-methoxy-1*H-*indole-2-carboxylate (36)

The reaction was carried out as described in general procedure C using **11** (0.100 g, 0.290 mmol, 1.0 equiv.) and 4-nitro-3–(4,4,5,5-tetramethyl-1,3,2-dioxaborolan-2-yl)aniline **23** (0.153 g, 0.579 mmol, 2.0 equiv.). Purification by flash chromatography on silica gel (eluent 8/2 PE/EtOAc) afforded **36** (0.041 g, 40%) as a yellow solid, which was used in the next step. R_f_ = 0.3 (PE/EtOAc 8/2). ^1^H NMR (CDCl_3_, 400 MHz) 1.14 (*t*, *J =* 7.2 Hz, 3H), 3.76 (*s*, 3H), 4.14 (*q*, *J =* 7.2 Hz, 2H), 5.38 (*s*, 2H), 6.62 (d, *J =* 2.4 Hz, 1H), 6.68 (dd, *J =* 2.4 Hz, 8.8 Hz, 1H), 6.78 (d, *J =* 2.4 Hz, 1H), 6.95 (dd, *J =* 2.4 Hz, 8.8 Hz, 1H), 7.41 (d, *J =* 8.8 Hz, 1H), 8.03 (d, *J =* 8.8 Hz, 1H), 11.05 (*s*, 1H ESI-MS *m*/*z* 356 [M + H]^+^. IR (KBr) *ν* 3400–3200 (br.), 3014, 2912, 1682, 1618, 1514, 1402, 1102, 1009, 980, 745 cm^−1^.

#### Ethyl 3–(5-acetamido-2-nitrophenyl)-5-methoxy-1*H-*indole-2-carboxylate (37)

The reaction was carried out as described in general procedure C using **11** (0.100 g, 0.290 mmol, 1.0 equiv.) and *N*-(4-nitro-3–(4,4,5,5-tetramethyl-1,3,2-dioxaborolan-2-yl)phenyl) acetamide **24** (0.177 g, 0.579 mmol, 2.0 equiv.). Purification by flash chromatography on silica gel (eluent 9/1 PE/EtOAc) afforded **37** (0.063 g, 55%) as a yellow solid. R_f_ = 0.4 (PE/EtOAc 8/2). ^1^H NMR (CDCl_3_, 400 MHz) 1.06 (t, *J =* 6.8 Hz, 3H), 2.09 (s, 3H), 3.68 (s, 3H), 4.05–4.11 (*m*, *J =* 6.8, 2H), 6.74 (d, *J =* 2.4 Hz, 1H), 6.99 (dd, *J =* 2.4 Hz, 9.2 Hz, 1H), 7.42 (d, *J =* 9.2 Hz, 1H), 7.74 (d, *J =* 2.0 Hz, 1H), 7.79 (dd, *J =* 2.0 Hz, 9.2 Hz, 1H), 8.12 (d, *J =* 9.2 Hz, 1H), 10.45 (*s*, 1H), 11.95 (*s*, 1H). ^13 ^C NMR (DMSO-d_6_, 125 MHz) δ 14.2, 24.8, 55.8, 60.9, 100.3, 114.4, 117.3, 117.8, 118.0, 122.7, 123.6, 126.6, 127.1, 131.0, 132.1, 143.7, 144.2, 155.0, 161.3, 169.8. ESI-MS *m*/*z* 398 [M + H]^+^. IR (KBr) *ν* 3352, 3301, 3117, 3057, 2986, 2938, 1710, 1698, 1621, 1405, 1264, 1118, 1034, 973, 763 cm^−1^.

#### Ethyl 6-methoxy-3–(2-nitrophenyl)-1*H-*indole-2-carboxylate (38)

The reaction was carried out as described in general procedure C using **12** (0.100 g, 0.290 mmol, 1.0 equiv.) and (2-nitrophenyl)boronic acid **15** (0.115 g, 0.579 mmol, 2.0 equiv). Purification by flash chromatography on silica gel (eluent 9/1 PE/EtOAc) afforded **38** (0.052 g, 53%) as a yellow solid. R_f_ = 0.4 (PE/EtOAc 8/2). ^1^H NMR (CDCl_3_, 400 MHz) 1.10 (*t*, *J =* 7.2 Hz, 3H), 3.85 (*s*, 3H), 4.15(*q*, *J =* 7.2 Hz, 2H), 6.81 (dd, *J =* 2.4, 8.8 Hz, 1H), 6.85 (d, *J =* 1.6 Hz, 1H), 7.28 (d, *J =* 8.8 Hz, 1H), 7.48–7.54(*m*, 2H), 7.62–7.66 (*m*, 1H), 8.06 (dd, *J =* 0.8, 8 Hz, 1H), 9.07 (*s*, 1H). ^13 ^C NMR (DMSO-d_6_,125 MHz) 14.1, 55.7, 60.7, 94.5, 113.0, 118.4, 121.4, 121.6, 122.2, 124.6, 129.1, 133.2, 133.6, 137.9, 150.2, 158.8, 161.3. ESI-MS *m*/*z* 341 [M + H]^+^. IR (KBr) *ν* 3350, 3050, 2918, 1706, 1620, 1555, 1450, 1279, 1133, 1043, 984, 732 cm^−1^.

#### Ethyl 6-methoxy-3–(5-methoxy-2-nitrophenyl)-1*H-*indole-2-carboxylate (39)

The reaction was carried out as described in general procedure C using **12** (0.100 g, 0.290 mmol, 1.0 equiv.) and (5-methoxy-2-nitrophenyl)boronic acid **17** (0.113 g, 0.579 mmol, 2.0 equiv. Purification by flash chromatography on silica gel (eluent 8/2 PE/EtOAc) afforded **39** (0.062 g, 58%) as a yellow solid. R_f_ = 0.35 (PE/EtOAc 8/2). ^1^H NMR (CDCl_3_, 400 MHz) 1.13 (*t*, *J =* 7,2 Hz, 3H), 3.86 (*s*, 3H), 3.88 (*s*, 3H), 4.15 (*q*, *J =* 7.2 Hz, 2H), 6.82 (d, *J =* 8.4 Hz, 1H), 6.86 (*s*, 1H), 6.93 (*s*, 1H), 6.98 (d, *J =* 9.2 Hz, 1H), 7.32 (d, *J =* 8.4 Hz, 1H), 8.15 (d, *J =* 9.2 Hz, 1H), 7.32 (d, *J =* 8.4 Hz, 1H), 8.15 (d, *J =* 9.2 Hz, 1H), 9.0 (*s*, 1H). ^13 ^C NMR (DMSO-d_6,_ 125 MHz) 14.2, 55.8, 56.6, 60.8, 94.6, 113.0, 113.9, 116.7, 118.5, 121.5, 122.1, 127.4, 132.2, 137.9, 140.0, 143., 158.9, 161.4, 162.7. ESI-MS *m*/*z* 371 [M + H]^+^. IR (KBr) *ν* 3040, 2948, 1710, 1615, 1534, 1450, 1273, 1047, 990, 734 cm^−1^.

#### Ethyl 5-fluoro-3–(5-methoxy-2-nitrophenyl)-1*H-*indole-2-carboxylate (40)

The reaction was carried out as described in general procedure C using **13** (0.100 g, 0.300 mmol, 1.0 equiv.) and (5-methoxy-2-nitrophenyl)boronic acid **17** (0.118 g, 0.600 mmol, 2.0 equiv.). Purification by flash chromatography on silica gel (eluent 9/1 PE/EtOAc) afforded **40** (0.066 g, 61%) as a yellow solid, which was used in the next step. R_f_ = 0.4 (PE/EtOAc 8/2). ^1^H NMR (CDCl_3_, 400 MHz) 1.17 (*t*, *J =* 7.2 Hz, 3H), 3.92 (s, 3H), 4.21 (*q*, *J =* 7.2 Hz, 2H), 6.94 (d, *J =* 2.4 Hz, 1H), 8.20 (d, *J =* 9.2 Hz, 1H), 7.44 (dd, *J =* 2.4 Hz, 9.2 Hz, 1H), 7.10–7.19 (*m*, 3H). ESI-MS *m*/*z* 359 [M + H]^+^. IR (KBr) *ν* 3205, 3092, 2952, 2914, 1698, 1623, 1525, 1405, 1273, 1176, 1047, 936, 724 cm^−1^.

#### Ethyl 5-fluoro-3–(5-hydroxy-2-nitrophenyl)-1*H-*indole-2-carboxylate (41)

The reaction was carried out as described in general procedure C using **13** (0.100 g, 0.300 mmol, 1.0 equiv.) and 4-nitro-3–(4,4,5,5-tetramethyl-1,3,2-dioxaborolan-2-yl)phenol **22** (0.159 g, 0.600 mmol, 2.0 equiv.). Purification by flash chromatography on silica gel (eluent 8/2 PE/EtOAc) afforded **41** (0.053 g, 52%) as a yellow solid. R_f_ = 0.3 (PE/EtOAc 8/2). ^1^H NMR (CDCl_3_, 400 MHz) 1.17 (*t*, *J =* 6.8 Hz, 3H), 4.20 (*q*, *J =* 6.8 Hz, 2H), 6.81 (*s*, 1H), 7.00 (d, *J =* 6.8 Hz, 1H), 7.06 (d, *J =* 8.0 Hz, 1H), 7.21 (*t*, *J =* 6.8 Hz, 1H), 7.53 (*s*, 1H), 8.09 (d, *J =* 7.6 Hz, 1H), 10.88 (*s*, 1H), 12.19 (*s*, br, 1H). ^13 ^C NMR (DMSO-d_6,_ 125 MHz) 14.1, 61.1, 104.8, 114.8, 115.3, 119.6, 124.9, 126.7, 127.01, 127.9, 131.8, 133.5, 141.5, 156.9, 159.3, 161.1, 162.0. ESI-MS *m*/*z* 345 [M + H]^+^. IR (KBr) *ν* 3412, 3150, 3052, 2908, 1704, 1623, 1565, 1453, 1271, 1137, 1043, 984, 734 cm^−1^.

#### Ethyl 3–(5-hydroxy-2-nitrophenyl)-5-methoxy-1-methyl-1*H-*indole-2-carboxylate (42)

The reaction was carried out as described in general procedure C using **14** (0.100 g, 0.278 mmol, 1.0 equiv.) and 4-nitro-3–(4,4,5,5-tetramethyl-1,3,2-dioxaborolan-2-yl)phenol **22** (0.148 g, 0.557 mmol, 2.0 equiv.) and NaHCO_3_ (0.070 g, 0.834 mmol, 3.0 equiv.). Purification by flash chromatography on silica gel (eluent 9/1 PE/EtOAc) afforded **42** (0.065 g, 63%) as a yellow solid. R_f_ = 0.4 (PE/EtOAc 9/1). ^1^H NMR (CDCl_3_, 400 MHz) 1.04 (*t*, *J =* 7.2 Hz, 3H), 3.79(*s*, 3H), 4.06 (*s*, 3H), 4.12 (*q*, *J =* 7.2 Hz, 2H), 6.31 (*s*, 1H, br), 6.72 (dd, *J =* 8.2 Hz, 1.4 Hz, 1H), 6.77 (dd, *J =* 7.6 Hz, 2.1 Hz, 1H) 6.92 (d, *J =* 7.6 Hz, 1H), 6.95 (d, *J =* 8.2 Hz, 1H), 7.14 (d, *J =* 1.4 Hz, 1H), 7.75 (d, *J =* 2.1 Hz, 1H). ^13 ^C NMR (DMSO-d_6_, 125 MHz) 13.8, 32.7, 55.8, 60.8, 100.5, 112.9, 115.2, 117.3, 119.5, 124.6, 125.9, 126.0, 127.8, 133.1, 134.2, 141.7, 155.2, 161.5, 162.0. ESI-MS *m*/*z* 371 [M + H]^+^. IR (KBr) *ν* 3040, 2948, 1710, 1615, 1534, 1450, 1273, 1047, 990, 734 cm^−1^.

#### Ethyl 5-methoxy-1-methyl-3–(2-nitro-5-(trifluoromethyl)phenyl)-1*H-*indole-2-carboxylate (43)

The reaction was carried out as described in general procedure C using **14** (0.100 g, 0.278 mmol, 1.0 equiv.) and (2-nitro-5-(trifluoromethyl)phenyl)boronic acid **19** (0.131 g, 0.557 mmol, 2.0 equiv.). Purification by flash chromatography on silica gel (eluent 9/1 PE/EtOAc) afforded **43** (0.063 g, 54%) as a yellow solid. R_f_ = 0.4 (PE/EtOAc 8/2). ^1^H NMR (CDCl_3_, 400 MHz) 0.99 (*t*, *J =* 6.8 Hz, 3H), 3.78 (*s*, 3H), 4.09–4.16 (*m*, 5H, N-CH_3_ and CH_2_), 6.68 (dd, *J =* 2.4 Hz, 10.8 Hz, 1H), 7.11 (dd, *J =* 2.4 Hz, 8.8 Hz, 1H), 7.38 (d, *J =* 8.8 Hz, 1H), 7.79 (*s*, 1H), 7.82 (d, *J =* 8.4 Hz, 1H), 8.15 (d, *J =* 8.4 Hz, 1H). ^13 ^C NMR (DMSO-d_6_, 125 MHz) 13.6, 32.9, 55.8, 61.0, 100.2, 113.1, 116.5, 117.6, 125.2, 125.6, 125.9, 126.1, 126.4, 130.8, 130.9, 134.1, 152.5, 155.4, 155.5, 161.1. ESI-MS *m*/*z* 423 [M + H]^+^. IR (KBr) *ν* 3051, 2959, 2912, 1689, 1616, 1409, 1104, 1012, 979, 746 cm^−1^.

#### Ethyl 3–(5-(dimethylamino)-2-nitrophenyl)-5-methoxy-1-methyl-1*H-*indole-2-carboxylate (44)

The reaction was carried out as described in general procedure C using **14** (0.100 g, 0.278 mmol, 1.0 equiv.) and (2-(dimethylamino)-5-nitrophenyl)boronic acid **20** (0.117 g, 0.557 mmol, 2.0 equiv.). Purification by flash chromatography on silica gel (eluent 9/1 PE/EtOAc) afforded **44** (0.072 g, 65%) as a yellow solid, which was used in the next step. R_f_ = 0.5 (PE/EtOAc 8/2). ^1^H NMR (CDCl_3_, 400 MHz) 1.00 (*t*, *J =* 7.2 Hz, 3H), 3.07 (*s*, 6H), 3.75 (*s*, 3H), 4.05–4.11 (*m*, 5H), 6.55 (d, *J =* 2.8 Hz, 1H), 6.66 (dd, *J =* 2.8 Hz, 9.2 Hz, 1H), 6.76 (d, *J =* 2.4 Hz, 1H), 7.05 (dd, *J =* 2.4 Hz, 8.8 Hz, 1H), 7.33 (d, *J =* 8.8 Hz, 1H), 8.19 (d, *J =* 9.2 Hz, 1H). ESI-MS *m*/*z* 398 [M + H]^+^. IR (KBr) *ν* 3045, 2980, 2928, 1701, 1612, 1515, 1403,1241,1107,1032, 975,729 cm^−1^.

#### General procedure D: synthesis of the indolo[2,3-*c*]quinolin-6-one library 45–64

Ethyl 3-(substituted-2-nitrophenyl)-1*H*-substituted-indole-2-carboxylate **25–44 (**1.0 equiv.) was dissolved in acetic acid and Iron (Fe) powder (5.0 equiv.) was added. The reaction mixture was heated to 110 °C for about 12 h. After completion of the reaction, the acetic acid was removed under reduced pressure and the residue was diluted with EtOAc. After filtration and evaporation under reduced pressure, the crude material was purified by silica gel flash chromatography (CH_3_OH/CH_2_Cl_2_).

#### 5,7-Dihydro-6*H*-indolo[2,3-*c*]quinolin-6-one (45)

The reaction was carried out as described in general procedure D using **25** (0.090 g, 0.29 mmol, 1.0 equiv.). Purification by flash chromatography on silica gel (eluent 9/1 CH_2_Cl_2_/MeOH) afforded **45** (0.054 g, 80%) as a white solid. R_f_ = 0.5 (5% CH_3_OH in CH_2_Cl_2_). mp 233–237 °C. ^1^H NMR (DMSO-d_6,_ 400 MHz) 7.31–7.36 (*m*, 2H), 7.42(d, *J =* 7.6 Hz, 1H) 7.46–7.53(*m*, 2H), 7.65(d, *J =* 8 Hz, 1H), 8.45 (d, *J =* 7.6 Hz, 1H), 8.48 (d, *J =* 8.0 Hz, 1H), 11.87 (*s*, 1H, Imid-NH), 12.37 (*s*, 1H, amide-NH).^13^C NMR (DMSO-d_6,_ 125 MHz) 113.5, 116.5, 118.5, 118.7, 121.1, 122.7, 122.8, 123.4, 126.1, 126.3, 128.1, 135.4, 139.3, 156.2. ESI-MS *m*/*z* 235 [M + H]^+^. HRMS (TOF-MS): *m*/*z* calcd for C_15_H_11_N_2_O [M + H]^+^: 235.0871, found: 235.0861; IR(KBr) *ν* 3319, 3157, 3004, 1655, 1621, 1594, 1254, 731 cm^−1^.

#### 2-Methoxy-5,7-dihydro-6*H*-indolo[2,3-*c*]quinolin-6-one (46)

The reaction was carried out as described in general procedure D using **26** (0.070 g, 0.205 mmol, 1.0 equiv). Purification by flash chromatography on silica gel (eluent 9/1 CH_2_Cl_2_/MeOH) afforded **46** (0.038 g, 70%) as a white solid. R_f_ = 0.4 (5% CH_3_OH in CH_2_Cl_2_). mp >250 °C. ^1^H NMR (DMSO-d_6_, 400 MHz) 3.94 (*s*, 3H), 7.07 (dd, *J =* 2.4 Hz, 8.8 Hz, 1H), 7.33 (*t*, *J =* 7.2 Hz, 7.6 Hz, 1H), 7.45 (d, *J =* 8.8 Hz, 1H), 7.49 (d, *J =* 7.6 Hz, 1H), 7.65 (*t*, *J =* 7.2 Hz, 8.4 Hz, 1H), 7.80 (d, *J =* 2.4 Hz, 1H), 8.45 (d, *J =* 8.4 Hz, 1H), 11.73 (*s*, 1H), 12.32 (*s*, 1H). ^13 ^C NMR (DMSO-d_6_, 125 MHz) 56.0, 106.1, 113.5, 114.5, 117.7, 118.3, 119.2, 121.1, 122.7, 126.0, 128.5, 129.6, 139.2, 155.3, 155.6. ESI-MS *m*/*z* 265 [M + H]^+^. HRMS (TOF-MS): *m*/*z* calcd for C_16_H_13_N_2_O_2_ [M + H]^+^: 265.0977, found: 265.0965. *m*/*z* calcd for C_15_H_10_N_2_O_2_Na [M + Na]^+^: 287.1768, found: 287.1752. IR (KBr) *ν* 3150, 3041, 2928, 1659, 1601, 1574, 1202, 1137, 1020, 764 cm^−1^.

#### 3-Hydroxy-5,7-dihydro-6*H*-indolo[2,3-*c*]quinolin-6-one (47)

The reaction was carried out as described in general procedure D using **27** (0.050 g, 0.153 mmol, 1.0 equiv). The crude mixture was purified by flash chromatography on silica gel (eluent 9/1 CH_2_Cl_2_/MeOH) and afforded **47** (0.030 g, 71%) as a white solid. R_f_ = 0.4 (5% CH_3_OH in CH_2_Cl_2_). mp 238–240 °C (decomp.). ^1^H NMR (DMSO-d_6_, 400 MHz) 6.81 (dd, *J =* 2.0 Hz, 8.4 Hz, 1H), 6.92 (d, *J =* 2.0 Hz, 1H), 7.26 (*t*, *J =* 7.6 Hz, 8.0 Hz, 1H), 7.43 (*t*, *J =* 7.6 Hz, 8.0 Hz, 1H), 7.59 (d, *J =* 8.0 Hz, 1H), 8.24 (d, *J =* 8.4 Hz, 1H), 8.39 (d, *J =* 8.0 Hz, 1H), 9.73 (*s*, br, 1H), 11.65 (*s*, 1H), 12.09 (*s*, 1H). ^13 ^C NMR (DMSO-d_6_, 125 MHz) 101.8, 102.1, 111.1, 119.5, 120.6, 120.7, 122.4, 122.7, 124.5, 125.9, 126.0, 126.2, 137.0, 139.3, 156.4; ESI-MS *m*/*z* 251 [M + H]^+^. HRMS (TOF-MS): *m*/*z* calcd for C_15_H_11_N_2_O_2_ [M + H]^+^: 251.0820, found: 251.0810. IR (KBr) *ν* 3425, 3234, 3135, 3021, 1665, 1610, 1587, 1130, 754 cm^−1^.

#### 2-Hydroxy-5,7-dihydro-6*H*-indolo[2,3-*c*]quinolin-6-one (48)

The reaction was carried out as described in general procedure D using **28** (0.055 g, 0.169 mmol, 1.0 equiv.). The crude mixture was purified by flash chromatography on silica gel (eluent 9/1 CH_2_Cl_2_/MeOH) to afford **48** (0.032 g, 75%) as a white solid. R_f_ = 0.4 (5% CH_3_OH in CH_2_Cl_2_). mp 235–238 °C. ^1^H NMR (DMSO-d_6_, 400 MHz) 6.90 (d, *J =* 8.0 Hz, 1H), 7.28–7.36 (*m*, *J =* 8.0 Hz, 2H), 7.46 (*t*, *J =* 7.2 Hz, 6.8 Hz, 1H), 7.64 (d, *J =* 8.0 Hz, 1H), 7.79 (*s*, 1H), 8.31 (d, *J =* 7.2 Hz, 1H), 10.94 (*s*, br, 1H), 11.67 (*s*, 1H), 12.28 (*s*, 1H). ^13 ^C NMR (DMSO-d_6_, 125 MHz) 108.0, 113.5, 115.2, 117.5, 118.3, 119.3, 121.0, 122.2, 122.8, 122.8, 128.4, 128.5, 139.2, 153.4, 155.5. ESI-MS *m*/*z* 251 [M + H]^+^. HRMS (TOF-MS): *m*/*z* calcd for C_15_H_11_N_2_O_2_ [M + H]^+^: 251.0820, found: 251.0815. IR (KBr) *ν* 3410, 3213, 3150, 3056, 1675, 1611, 1554, 1210,1130, 784 cm^−1^.

#### 10-Methoxy-5,7-dihydro-6*H*-indolo[2,3-*c*]quinolin-6-one (49)

The reaction was carried out as described in general procedure D using **29** (0.050 g, 0.147 mmol, 1.0 equiv.). The crude mixture was purified by flash chromatography on silica gel (eluent 9/1 CH_2_Cl_2_/MeOH) to afford **49** (0.032 g, 83%) as a white solid. R_f_ = 0.5 (5% CH_3_OH in CH_2_Cl_2_); mp 232–235 °C; ^1^H NMR (DMSO-d_6,_ 400 MHz) 3.94 (3H,s, -OCH_3_), 7.14 (dd, *J =* 2.0, 6.8 Hz, 1H), 7.36 (*t*, *J* = 6.8 Hz, 1H), 7.38 (*t*, J = 7.2 Hz, 1H), 7.50 (d, *J* = 7.6 Hz, 1H), 7.54 (d, *J* = 7.6 Hz, 1H), 7.83 (d, *J =* 2.0 Hz, 1H), 8.44 (dd, *J =* 7.2 Hz, 1H); ^13 ^C NMR (DMSO-d_6,_ 125 MHz) 56.2, 103.8, 114.3, 116.4, 116.9, 118.2, 118.6, 122.7, 122.9, 123.4, 126.1, 128.4, 134.4, 135.2, 154.8, 156.2; ESI-MS *m*/*z* 265 [M + H]^+^; HRMS (TOF-MS): *m*/*z* calcd for C_16_H_13_N_2_O_2_ [M + H]^+^: 265.0977, found: 265.0972; IR (KBr) *ν* 3310, 3135, 3044,2948, 1660, 1611, 1583, 1204, 1021, 740 cm^−1^.

#### 3,10-Dimethoxy-5,7-dihydro-6*H*-indolo[2,3-*c*]quinolin-6-one (50)

The reaction was carried out as described in general procedure D using **30** (0.040 g, 0.108 mmol, 1.0 equiv.). The crude mixture was purified by flash chromatography on silica gel (eluent 9/1 CH_2_Cl_2_/MeOH) to afford **50** (0.024 g, 75%) as a white solid. R_f_ = 0.5 (5% CH_3_OH in CH_2_Cl_2_); mp 192–195 °C; ^1^H NMR (DMSO-d_6,_ 400 MHz) 3.83 (*s*, 3H), 3.92(*s*, 3H), 6.95 (d, *J =* 8.8 Hz, 1H), 7.05 (*s*, 1H), 7.11 (d, *J =* 8.8 Hz, 1H), 7.51 (d, *J =* 8.8 Hz, 1H), 7.78 (*s*, 1H), 8.34 (d, *J =* 8.8 Hz, 1H), 11.67 (*s*, 1H), 12.06 (*s*, 1H); ^13 ^C NMR (DMSO-d_6,_ 125 MHz) 55.6, 56.1, 100.3, 103.6, 110.7, 112.4, 114.3, 117.0, 118.8, 122.5, 124.6, 126.9, 134.5, 136.6, 154.6, 156.4, 158.0; ESI-MS *m*/*z* 295 [M + H]^+^; HRMS (TOF-MS): *m*/*z* calcd for C_17_H_15_N_2_O_3_ [M + H]^+^: 295.1082, found: 295.1070; IR(KBr) *ν* 3265, 3105, 3060, 2981, 1668, 1612, 1564, 1210, 1150, 1026, 755 cm^−1^_._

#### 2,10-Dimethoxy-5,7-dihydro-6*H*-indolo[2,3-*c*]quinolin-6-one (51)

The reaction was carried out as described in general procedure D using **31** (0.050 g, 0.135 mmol, 1.0 equiv.). The crude mixture was purified by flash chromatography on silica gel (eluent 9/1 CH_2_Cl_2_/MeOH) to afford **51** (0.027 g, 68%) as a white solid. R_f_ = 0.5 (5% CH_3_OH in CH_2_Cl_2_). mp 190–192 °C. ^1^H NMR (DMSO-d_6,_ 400 MHz) 3.93 (*s*, 6H), 7.06 (dd, *J* = 2.0 Hz, 8.8 Hz, 1H), 7.14 (dd, *J* = 2.0 Hz, 7.2 Hz, 1H), 7.42 (d, *J =* 8.8 Hz, 1H), 7.65 (d, *J =* 8.8 Hz, 1H), 7.75 (*s*, 2H), 11.70 (*s*, 1H, NH), 12.23 (*s*, 1H, NH). ^13 ^C NMR (DMSO-d_6_, 125 MHz) 55.7, 56.0, 95.2, 105.8, 112.1, 114.7, 116.8, 117.7, 118.9, 119.0, 123.7, 127.7, 129.8, 140.7, 155.2, 155.4, 158.8. ESI-MS *m*/*z* 295 [M + H]^+^. HRMS (TOF-MS): *m*/*z* calcd for C_17_H_15_N_2_O_3_ [M + H]^+^: 295.1082, found: 295.1079; IR (KBr) *ν* 3290, 3107, 3051,2963, 1683, 1631, 1551, 1204, 737 cm^−1^.

#### 2,3,10-Trimethoxy-5,7-dihydro-6*H*-indolo[2,3-*c*]quinolin-6-one (52)

The reaction was carried out as described in general procedure D using **32** (0.060 g, 0.15 mmol, 1.0 equiv). The crude mixture was purified by flash chromatography on silica gel (eluent 9/1 CH_2_Cl_2_/MeOH) to afford **52** (0.031 g, 65%) as a white solid. This compound was not soluble enough to provide a correct ^13 ^C NMR spectrum. R_f_ = 0.4 (5% CH_3_OH in CH_2_Cl_2_). mp 205–208 °C. ^1^H NMR (DMSO-d_6,_ 400 MHz) 3.82 (*s*, 3H), 3.91 (*s*, 3H), 3.97 (*s*, 3H), 7.10 (*s*, 1H), 7.13 (*s*, 1H), 7.51 (*s*, 1H), 7.72 (d, *J =* 12.4 Hz, 2H), 11.57 (*s*, 1H), 12.01 (*s*, 1H); ESI-MS *m*/*z* 325 [M + H]^+^. HRMS (TOF-MS): *m*/*z* calcd for C_18_H_16_N_2_O_4_ [M + H]^+^: 325.1188, found: 325.1182. IR (KBr) *ν* 3259, 3161, 3024,2982, 2920, 1667, 1621, 1575, 1234,1205, 1102, 747 cm^−1^.

#### 10-Methoxy-2-(trifluoromethyl)-5,7-dihydro-6*H*-indolo[2,3-*c*]quinolin-6-one (53)

The reaction was carried out as described in general procedure D using **33** (0.050 g, 0.122 mmol, 1.0 equiv.). The crude mixture was purified by flash chromatography on silica gel (eluent 9/1 CH_2_Cl_2_/MeOH) to afford **53** (0.031 g, 75%) as a white solid. R_f_ = 0.4 (5% CH_3_OH in CH_2_Cl_2_). mp 241–244 °C. ^1^H NMR (DMSO-d_6_, 400 MHz) 3.93 (*s*, 3H), 7.20 (dd, *J =* 2.0 Hz, 8.8 Hz, 1H), 7.59 (d, *J =* 8.8 Hz, 1H), 7.66 (d, *J =* 8.8 Hz, 1H), 7.75 (d, *J =* 2.0 Hz, 1H), 8.52 (*s*, 1H), 12.15 (*s*, 1H), 12.44 (*s*, 1H). ^13 ^C NMR (DMSO-d_6_, 125 MHz) 56.1, 103.8, 114.6, 117.0, 117.2, 117.3, 118.5, 119.8, 119.8, 122.6, 122.9, 123.2, 128.9, 134.5, 137.7, 155.1, 156.2. ESI-MS *m*/*z* 333 [M + H]^+^. HRMS (TOF-MS): *m*/*z* calcd for C_17_H_12_N_2_O_2_F_3_ [M + H]^+^: 333.0851, found: 333.0841; IR (KBr) *ν* 3235, 3153, 3060, 2951, 1658, 1610, 1574, 1210,1030, 744 cm^−1^.

#### 3-Hydroxy-10-methoxy-5,7-dihydro-6*H*-indolo[2,3-*c*]quinolin-6-one (54)

The reaction was carried out as described in general procedure D using **34** (0.040 g, 0.112 mmol, 1.0 equiv.). The crude mixture was purified by flash chromatography on silica gel (eluent 9/1 CH_2_Cl_2_/MeOH) to afford **54** (0.023 g, 73%) as a white solid. R_f_ = 0.4 (5% CH_3_OH in CH_2_Cl_2_). mp 227–230 °C. ^1^H NMR (DMSO-d_6,_ 400 MHz) 3.92 (*s*, 3H), 6.82 (d, *J =* 8.4 Hz, 1H), 6.91(d, *J =* 1.2 Hz, 1H), 7.10 (d, *J =* 8.8 Hz, 1H),7.49 (d, *J =* 8.8 Hz, 1H), 7.76 (*s*, 1H), 8.24 (d, *J =* 8.4 Hz, 1H), 9.67 (*s*, br, 1H), 11.59 (*s*, 1H), 11.95 (*s*, 1H). ^13 ^C NMR (DMSO-d_6,_ 125 MHz) 56.1, 102.0, 103.7, 111.3, 111.9, 114.2, 116.9, 119.1, 122.4, 124.5, 126.6, 134.5, 136.8, 154.5, 156.2, 156.5; ESI-MS *m*/*z* 281 [M + H]^+^. HRMS (TOF-MS): *m*/*z* calcd for C_16_H_13_N_2_O_3_ [M + H]^+^: 281.0926, found: 281.0920. IR (KBr) *ν* 3450, 3250, 3141, 3020, 2983, 1643, 1620, 1594, 1223,1150, 724 cm^−1^.

#### 2-Hydroxy-10-methoxy-5,7-dihydro-6*H*-indolo[2,3-*c*]quinolin-6-one (55)

The reaction was carried out as described in general procedure D using **35** (0.040 g, 0.112 mmol, 1.0 equiv.). The crude mixture was purified by flash chromatography on silica gel (eluent 9/1 CH_2_Cl_2_/MeOH) to afford **55** (0.025 g, 78%) as a white solid. R_f_ = 0.4 (5% CH_3_OH in CH_2_Cl_2_). mp 202–205 °C. ^1^H NMR (DMSO-d_6,_ 400 MHz) 3.93 (*s*, 3H), 6.87 (*s*, 1H), 7.14 (*s*, 1H), 7.32 (*s*, 1H), 7.53 (*s*, 1H), 7.68–7.74 (*m*, 2H), 9.38 (*s*, br 1H), 11.59 (*s*, 1H), 12.16 (*s*, 1H). ^13 ^C NMR (DMSO-d_6,_ 125 MHz) 56.0, 103.4, 107.8, 114.4, 114.8, 116.5, 117.5, 117.9, 119.4, 122.9, 128.3, 128.7, 134.4, 153.1, 154.7, 155.6. ESI-MS *m*/*z* 281 [M + H]^+^; HRMS (TOF-MS): *m*/*z* calcd for C_16_H_13_N_2_O_3_ [M + H]^+^: 281.0926, found: 281.0918; IR (KBr) *ν* 3435, 3245, 3150, 3010, 2951, 1653, 1610, 1574, 1220,1130,1006, 734 cm^−1^.

#### 2-Amino-10-methoxy-5,7-dihydro-6*H*-indolo[2,3-*c*]quinolin-6-one (56)

The reaction was carried out as described in general procedure D using **36** (0.040 g, 0.112 mmol, 1.0 equiv.). The crude mixture was purified by flash chromatography on silica gel (eluent 9/1 CH_2_Cl_2_/MeOH) to afford **56** (0.022 g, 70%) as a white solid. R_f_ = 0.3 (5% CH_3_OH in CH_2_Cl_2_). mp >250 °C. ^1^H NMR (DMSO-d_6_, 400 MHz) 2.12 (*s*, 2H), 3.92 (*s*, 3H), 7.16 (dd, *J =* 1.6 Hz, 8.8 Hz, 1H), 7.40 (d, *J =* 8.8 Hz, 1H), 7.56–7.59 (*m*, 2H), 7.75 (d, *J =* 1.6 Hz, 1H), 8.77 (d, *J =* 1.6 Hz, 1H), 10.10 (*s*, 1H), 11.73 (*s*, 1H), 12.19 (*s*, 1H). ^13 ^C NMR (DMSO-d_6_, 125 MHz) 56.4, 103.9, 112.4, 114.3, 116.2, 116.4, 116.5, 118.0, 118.5, 122.8, 128.7, 130.9, 134.5, 134.8, 154.8, 155.9. ESI-MS *m*/*z* 280 [M + H]^+^. HRMS (TOF-MS): *m*/*z* calcd for C_16_H_13_N_3_O_2_ [M]^+^: 279.1008, found: 279.0929. IR (KBr) *ν* 3285, 3157, 2993, 2957, 2930, 1675, 1628, 1410, 1147, 746 cm^−1^

#### *N*-(10-Methoxy-6-oxo-6,7-dihydro-5*H*-indolo[2,3-*c*]quinolin-2-yl)acetamide (57)

The reaction was carried out as described in general procedure D using **37** (0.060 g, 0.151 mmol, 1.0 equiv.). The crude mixture was purified by flash chromatography on silica gel (eluent 9/1 CH_2_Cl_2_/MeOH) to afford **57** (0.033 g, 69%) as a white solid. R_f_ = 0.4 (5% CH_3_OH in CH_2_Cl_2_). mp 242–245 °C. ^1^H NMR (DMSO-*d*_6_, 400 MHz) 2.11 (*s*, 3H), 3.91 (*s*, 3H), 7.16 (d, *J =* 7.6 Hz, 1H), 7.38 (d, *J =* 8.8 Hz, 1H), 7.53–7.57 (*t*, *J =* 8.8 Hz, 2H), 7.75 (*s*, 1H), 8.76 (*s*, 1H), 10.05 (*s*, 1H), 11.69 (*s*, 1H), 12.17 (*s*, 1H). ^13 ^C NMR (DMSO-d_6_, 125 MHz) 24.6, 56.1,103.9, 112.4, 114.4, 116.3, 116.5, 117.7, 118.0, 118.5, 122.8, 128.7, 131.0, 134.5, 134.8, 154.8, 155.8; ESI-MS *m*/*z* 322 [M + H]^+^. HRMS (TOF-MS): *m*/*z* calcd for C_18_H_16_N_3_O_3_ [M + H]^+^: 322.1192, found: 322.1183. IR (KBr) *ν* 3284, 3157, 3088, 2992, 2929,1674, 1628, 1574, 1254,1110, 754 cm^−1^_._

#### 9-Methoxy-5,7-dihydro-6*H*-indolo[2,3-*c*]quinolin-6-one (58)

The reaction was carried out as described in general procedure D using **38** (0.050 g, 0.147 mmol, 1.0 equiv.). The crude mixture was purified by flash chromatography on silica gel (eluent 9/1 CH_2_Cl_2_/MeOH) to afford **58** (0.028 g, 72%) as a white solid. R_f_ = 0.5 (5% CH_3_OH in CH_2_Cl_2_). mp 235–237 °C. ^1^H NMR (DMSO-d_6_, 400 MHz) 3.85 (*s*, 3H), 6.93 (dd, *J =* 1.6 Hz, 8.8 Hz, 1H), 7.05 (d, *J =* 1.6 Hz, 1H), 7.30 (*t*, *J =* 7.2 Hz, 8.0 Hz, 1H), 7.39 (*t*, *J =* 7.2 Hz, 8.4 Hz, 1H), 7.48 (d, *J =* 8.4 Hz, 1H), 8.32 (d, *J =* 8.8 Hz, 1H), 8.37 (d, *J =* 8.0 Hz, 1H), 11.75 (*s*, 1H), 12.17 (*s*, 1H). ^13 ^C NMR (DMSO-d_6_, 125 MHz) 55.4, 95.2, 95.4, 111.8, 112.1, 116.8, 118.2, 119.2, 122.5, 123.6, 126.5, 127.3, 135.5, 140.7, 155.9, 158.9. ESI-MS *m*/*z* 265 [M + H]^+^. HRMS (TOF-MS): *m*/*z* calcd for C_16_H_13_N_2_O_2_ [M + H]^+^: 265.0977, found: 265.0967. IR (KBr) *ν* 3235, 3155, 3027, 2938, 1655, 1611, 1574, 1107, 1027, 734 cm^−1^.

#### 2,9-Dimethoxy-5,7-dihydro-6*H*-indolo[2,3-*c*]quinolin-6-one (59)

The reaction was carried out as described in general procedure D using **39** (0.055 g, 0.148 mmol, 1.0 equiv.). The crude mixture was purified by flash chromatography on silica gel (eluent 9/1 CH_2_Cl_2_/MeOH) to afford **59** (0.029 g, 67%) as a white solid. R_f_ = 0.4 (5% CH_3_OH in CH_2_Cl_2_). mp 235–237 °C. ^1^H NMR (DMSO-d_6,_ 400 MHz) 3.86 (*s*, 3H), 3.92 (*s*, 3H), 6.95 (d, *J =* 8.4 Hz, 1H), 7.05 (d, *J =* 7.6 Hz, 2H), 7.42 (d, *J =* 8.8 Hz, 1H), 7.74 (*s*, 1H), 8.31 (d, *J =* 8.8 Hz, 1H), 11.66 (*s*, 1H, NH), 12.18 (*s*, 1H, NH). ^13 ^C NMR (DMSO-d_6_, 125 MHz) 55.7, 56.0, 95.2, 105.8, 112.1, 114.7, 116.8, 117.7, 118.9, 119.0, 123.7, 127.7, 129.8, 140.7, 155.2, 155.4,158.8. ESI-MS *m*/*z* 295 [M + H]^+^. HRMS (TOF-MS): *m*/*z* calcd for C_17_H_15_N_2_O_3_ [M + H]^+^: 295.1082, found: 295.1075. IR (KBr) *ν* 3304, 3125, 3050,2941,1645,1634, 1582, 1204, 741 cm^−1^.

#### 10-Fluoro-2-methoxy-5,7-dihydro-6*H*-indolo[2,3-*c*]quinolin-6-one (60)

The reaction was carried out as described in general procedure D using **40** (0.060 g, 0.167 mmol, 1.0 equiv.). The crude mixture was purified by flash chromatography on silica gel (eluent 9/1 CH_2_Cl_2_/MeOH) to afford **60** (0.038 g, 80%) as a white solid. R_f_ = 0.5 (5% CH_3_OH in CH_2_Cl_2_). mp 135–136 °C. ^1^H NMR (DMSO-d_6,_ 400 MHz) 3.95 (*s*, 3H), 7.07 (*s*, 1H), 7.36–7.45 (*m*, 2H), 7.64 (*s*, 1H), 7.73 (*s*, 1H), 8.25 (d, *J =* 7.2, 1H), 11.79 (*s*, 1H), 12.46 (*s*, 1H). ^13 ^C NMR (DMSO-d_6,_ 125 MHz) 56.1, 106.0, 107.6, 114.6, 114.7, 117.8, 118.9, 129.4, 129.9, 135.9, 155.4, 155.5. ESI-MS *m*/*z* 283 [M + H]^+^. HRMS (TOF-MS): *m*/*z* calcd for C_16_H_12_N_2_O_2_F [M + H]^+^: 283.0882, found: 283.0873. IR (KBr) *ν* 3303, 3126, 3034, 2953, 2890, 1663, 1621, 1564, 1254,1154, 1006, 745 cm^−1^.

#### 10-Fluoro-2-Hydroxy-5,7-dihydro-6*H*-indolo [2,3-*c*]quinolin-6-one (61)

The reaction was carried out as described in general procedure D using **41** (0.050 g, 0.145 mmol, 1.0 equiv.). The crude mixture was purified by flash chromatography on silica gel (eluent 9/1 CH_2_Cl_2_/MeOH) to afford **61** (0.031 g, 79%) as a white solid. R_f_ = 0.4 (5% CH_3_OH in CH_2_Cl_2_). mp 240–243 °C. ^1^H NMR (DMSO-d_6,_ 400 MHz) 6.90 (d, *J =* 8.4 Hz, 1H), 7.34–7.38 (*m*, 2H), 7.63–7.67 (*m*, 1H), 7.71 (*s*, 1H), 8.0 (d, *J =* 9.2, 1H), 9.35 (*s*, br, 1H), 11.7 (*s*, 1H), 12.42 (*s*, 1H). ^13 ^C NMR (DMSO-d_6,_ 125 MHz) 106.8, 107.0, 107.8, 114.5, 114.7, 114.8, 114.9, 115.3,117.7, 118.9, 128.4, 129.8, 135.9, 153.3, 155.5. ESI-MS *m*/*z* 269 [M + H]^+^. HRMS (TOF-MS): *m*/*z* calcd for C_15_H_10_N_2_O_2_F [M + H]^+^: 269.0726, found: 269.0721. IR (KBr) *ν* 3410, 3247, 3150, 3014, 2997, 2920, 1645, 1607, 1514, 1254, 1104, 732 cm^−1^_._

#### 2-Hydroxy-10-methoxy-7-methyl-5,7-dihydro-6*H*-indolo[2,3-*c*]quinolin-6-one (62)

The reaction was carried out as described in general procedure D using **42** (0.060 g, 0.162 mmol, 1.0 equiv.). The crude mixture was purified by flash chromatography on silica gel (eluent 9/1 CH_2_Cl_2_/MeOH) to afford **62** (0.037 g, 77%) as a white solid. R_f_ = 0.4 (5% CH_3_OH in CH_2_Cl_2_). mp >250 °C. ^1^H NMR (DMSO-d_6_, 400 MHz) 3.92 (*s*, 3H), 4.27 (*s*, 3H), 6.84 (*s*, 1H), 7.27 (d, *J =* 7.6 Hz, 1H), 7.65–7.73 (*m*, *J =* 7.6 Hz, 3H), 9.35 (*s*, br, 1H), 11.57 (*s*, 1H). ^13 ^C NMR (DMSO-d_6_, 125 MHz) 31.7, 56.1, 103.6, 107.7, 112.5, 115.0, 116.6, 117.3, 118.1, 119.2, 121.8, 127.1, 128.3, 135.8, 153.1, 155.0, 156.3. ESI-MS *m*/*z* 295 [M + H]^+^. HRMS (TOF-MS): *m*/*z* calcd for C_17_H_15_N_2_O_3_ [M + H]^+^: 295.1082 found: 295.1072. IR (KBr) *ν* 3450, 3180, 3070, 2954, 1661 1651, 1517, 1210,1105,1070, 739 cm^−1^.

#### 10-Methoxy-7-methyl-2-(trifluoromethyl)-5,7-dihydro-6*H*-Indolo[2,3-*c*]quinolin-6-one (63)

The reaction was carried out as described in general procedure D using **43** (0.055 g, 0.130 mmol, 1.0 equiv.) The crude mixture was purified by flash chromatography on silica gel (eluent 9/1 CH_2_Cl_2_/MeOH) to afford **63** (0.038 g, 84%) as a white solid. R_f_ = 0.7 (5% CH_3_OH in DCM). mp >250 ^0 ^C. ^1^H NMR (DMSO-d_6_, 400 MHz) 3.94 (*s*, 3H), 4.30 (*s*, 3H), 7.27 (d, *J* = 8.8 Hz, 1H), 7.61 (d, *J* = 8.4 Hz, 1H), 7.69–7.73 (*m*, *J* = 7.6 Hz, 8.4 Hz, 3H), 8.47 (*s*, 1H), 12.12 (*s*, 1H); ^13 ^C NMR: (DMSO-d_6,_ 125 MHz) 31.8, 56.1,103.7, 112.8, 116.9, 117.1, 117.4, 118.2, 119.6, 121.5, 122.6, 125.1, 127.2, 135.9, 137.6, 155.4, 156.8. ESI-MS: *m*/*z* 347 [M + H]^+^. HRMS (TOF-MS): *m*/*z* calcd for C_18_H_14_N_2_O_2_F_3_ [M + H]^+^: 347.1007, found: 347.1001. IR (KBr) *ν* 3110, 3010, 2951, 2910, 1657, 1594, 1200,1130,1010, 744 cm^−1^

#### 2-(Dimethylamino)-10-methoxy-7-methyl-5,7-dihydro-6*H*-indolo[2,3-*c*]quinolin-6-one (64)

The reaction was carried out as described in general procedure D using **44** (0.065 g, 0.163 mmol, 1.0 equiv.). The crude mixture was purified by flash chromatography on silica gel (eluent 9/1 CH_2_Cl_2_/MeOH) to afford **64** (0.039 g, 75%) as a white solid. R_f_ = 0.7 (5% CH_3_OH in DCM). mp 231–234 ^0 ^C. ^1^H NMR (DMSO-d_6_, 400 MHz) 3.00 (*s*, 6H), 3.91 (*s*, 3H), 4.28 (*s*, 3H), 6.94 (dd, *J* = 2.4 Hz, 8.8 Hz, 1H), 7.21 (dd, *J* = 9.2 Hz, 2.4 Hz, 1H), 7.31 (d, *J* = 9.2 Hz, 1H), 7.44 (d, *J* = 2.4 Hz, 1H), 7.65 (d, *J* = 8.8 Hz, 1H), 7.16 (d, *J* = 2.4 Hz, 1H), 11.51 (*s*, 1H). ^13 ^C NMR (DMSO-d_6_, 125 MHz) 31.7, 41.2, 55.9, 103.9, 104.9, 112.4, 113.6, 116.2, 116.9, 118.3, 119.2, 121.9, 126.9, 127.3, 135.9, 146.9, 154.8, 156.1; ESI-MS: *m*/*z* 322 [M + H]^+^. HRMS (TOF-MS). *m*/*z* calcd for C_19_H_20_N_3_O_2_ [M + H]^+^: 322.1556, found: 322.1555. IR (KBr) *ν* 3145, 3020, 2950, 2910, 1675, 1600, 1564, 1210, 754 cm^−1^.

### Cell culture

HCT116 cells were cultured in McCoy’s medium. SH-SY5Y, MDA-MB231 and U-2 OS cells were cultured in Dulbecco's modified Eagle's medium (DMEM) and hTERT RPE-1 cells in DMEM:F12 medium. All media were supplemented with 10% foetal calf serum and cells were cultured at 37 °C in a 5% CO_2_ humidified atmosphere.

### Cell viability

Cells were grown in 96-well plates in the presence of a fixed concentration of 25 μM of each compound (for cell viability primary assessment) or increasing concentrations of each compound (from 50 to 0.05 µM) for 48 h (for EC_50_ determination). Cell viability was then assessed using the CellTiter96 AQueous cell proliferation assay from Promega according to the manufacturer’s instructions. Each experiment was done in triplicate and EC_50_ were determined from the dose-response curves according to the signal given by the control (0.1% DMSO) set at 100% viability using Prism GraphPad software.

### 3D Spheroid viability

U-2 OS cells were seeded at 5000 cells per well and HCT116 at 1500 cells per well, in 96-well black ULA plates (Ultra Low Adherence, Corning). After centrifugation at 200 *g* for 10 min, spheroids were incubated at 37 °C for 3 days in order to reach 400 µm in diameter. Compounds were then added at a single dose (2.5, 5, or 10 μM) and cell viability was measured after 7 days using the CellTiter-Glo® 3D Cell Viability Assay (Promega) following the manufacturer's protocol. Luminescence was measured using an EnVision® plate reader (Perkin Elmer).

### Endogenous haspin activity measurement

U-2 OS cells were grown on glass coverslips, treated for 16 h than fixed with 4% paraformaldehyde in PBS, permeabilized by 0.15% Triton-X100 for 2 min, blocked for 15 min in 4% BSA in PBS and processed using standard immunofluorescence protocols. Primary antibodies included anti-phospho-Thr3 Histone H3 (1/1000 dilution, Millipore) and anti-α-tubulin (1/5000 dilution, clone B512, Sigma). Images were acquired with a Coolsnap HQ_2_ CCD camera (Photometrics) on a Zeiss Axio microscope (Carl Zeiss) using a 63x NA 1.40 objective. Image acquisition and processing were performed using Metamorph (Molecular Device). Quantification of signal intensity was performed using ImageJ software (NIH).

### Cell cycle analysis

After treatment with the compounds, cells were trypsinized and washed once in PBS. Cells were fixed for 1 h in ice-cold 70% ethanol, then washed once in PBS, centrifuged at 200 g and resuspended in a PBS buffer containing 100 µg/ml RNase A (Thermo Scientific) and 40 µg/ml propidium iodide (Life Technologies). DNA content was determined using a flow cytometer Attune^TM^ NxT (Thermofisher) and ten thousand events were collected in each run. The data were analysed using FCS Express 7 Pro software (De Novo).

### Kinase assays

Kinase activities were determined using the ADP-Glo methodology (ADP-Glo Kinase Assay; Promega) according to the assay described by Nguyen et al.^32^ except for MmCLK1. The later was assayed in the following buffer: 10 mM MgCl_2_, 1 mM EGTA, 1 mM DTT, 25 mM Tris-HCl pH 7.5, 50 µg/ml heparin, 0.15 mg/ml BSA, with 0.027 µg/µl of the following peptide: GRSRSRSRSRSR as substrate.

## Molecular modelling

### Structure preparation

Marvin was used for drawing chemical structures^33^. Structures were prepared with VSPrep, a workflow dedicated to the preparation of ligands for virtual screening^34^, and finally given as input to Glide, the docking software from the Schrödinger Molecular Modelling Suite 2019–01^31^. Structural data of CKL1 kinase complexed with a methyl 9-anilinothiazolo[5,4-f] quinazoline-2-carbimidate (EHT1610) compound were retrieved from the protein data bank^35^, PDB entry 6YTI (unpublished data). DYRK1A in complex with a pyrido[2,3-*d*]pyrimidine inhibitor, the *N*-(5-([(1*R*)-3-amino-1–(3-chlorophenyl)propyl]carbamoyl)-2-chlorophenyl)-2-methoxy-7-oxo-7,8-dihydropyrido[2,3-*d*]pyrimidine-6-carboxamide, was retrieved from the protein data bank^35^, PDB entry 4MQ1^36^. Structural data of Haspin kinase complexed with an imidazo[1,2-b]pyridazine derivative compound were retrieved from the protein data bank^35^, PDB entry 3F2N (unpublished data). The three high-resolution crystal structures of CLK1, DYRK1A and Haspin were superimposed before carrying out molecular docking experiments. All the receptors were prepared using the Protein Preparation Wizard workflow from the Schrödinger Molecular Modelling Suite 2019–01. Hydrogen atoms were added, water molecules were removed, the hydrogen network automatically was optimised and finally proteins were minimised (OLPS2005 force field) with a convergence criterion of RMSD on heavy atoms of 0.3 Å (other parameters were fixed to their default values).

### Docking parameters

Docking grids were centred and sized on crystalised ligands. Docking calculations were performed with extra precision. Ligand flexibility was considered and the option of sampling of ring conformation was activated. A maximum of 100 poses were generated and a post-docking minimisation was performed.

## Conclusion

We have synthesised a series of new Lamellarin analogues using the indolo[2,3-*c*]quinolone-6-one core. The analogues were obtained after a sequence involving (i) a palladium catalysed cross coupling reaction between 2-indolic esters and 2-nitrophenyl boronic acids as building blocks, and (ii) a cyclic lactam formation involving a reduction and an annelation. Twenty-two novel derivatives were synthesised and evaluated for their inhibitory activity on Haspin kinase and on a panel of 7 other protein kinases for selectivity assessment. Among this series, 8 compounds inhibited Haspin kinase with IC_50_ below 10 nM. Docking studies showed a double hydrogen bond between the lactam and the hinge region of the kinase. The most active compounds **49** and **55** possess IC_50_ of 1 and 2 nM respectively with selectivity towards the parent kinases DYRK1A and CLK1 between a 13 and 65-fold factor. Furthermore, the most selective compound **55** exerted an interesting cellular effect on the osteosarcoma U-2 OS cell line as well as on U-2 OS and colorectal carcinoma HTC116 spheroid viability. Additionally, we further validated the functionality of compound **55** on endogenous Haspin activity in cells. This interesting Haspin inhibitor will be used in further studies to develop efficient and selective Haspin inhibitors.

## Supplementary Material

Supplemental Material
