# Supplementary material for: Design and biological evaluation of substituted 5,7-dihydro-6H-indolo[2,3-c]quinolin-6-one as novel selective Haspin inhibitors
Source: J Enzyme Inhib Med Chem. 2022 Jun 7;37(1):1632–50. doi: 10.1080/14756366.2022.2082419 (PMC9186362; doi:10.1080/14756366.2022.2082419)
Supplement: Supplemental Material [file IENZ_A_2082419_SM0502.pdf]

Design and biological evaluation of substituted 5,7-dihydro-6*H*-indolo[2,3-*c*]quinolin-6-one as novel selective Haspin inhibitor

Sreenivas Avula[a], Peng Xudang[a], Lang Xingfen[a], Micky Tortorella[a], Béatrice Josselin[c,d], Stéphane Bach[c,d], Stephane Bourg[b], Pascal Bonnet[b], Frédéric Buron[b], Sandrine Ruchaud[c], Sylvain Routier[b] and Cleopatra Neagoie[a,*]

[a]*Guangzhou Institute of Biomedicine and Health, Chinese Academy of Science, 190 Kai Yuan Avenue, Science Park, Guanghzou, 510530, China*

[b]*Institut de Chimie Organique et Analytique, Université d'Orléans, UMR CNRS 7311, rue de Chartres, BP 6759, 45067 Orléans Cedex 2, France*

[c]*Sorbonne Université / CNRS UMR8227, Station Biologique, Place Georges Teissier, CS90074, 29688 Roscoff cedex, France*

[d]*Sorbonne Université / CNRS FR2424, Plateforme de criblage KISSf (Kinase Inhibitor Specialized Screening facility) Station Biologique, Place Georges Teissier, CS90074, 29688 Roscoff cedex, France*

[*] Corresponding author: cleopatra.neagoie@gmail.com

The supporting information contains the the [1H] NMR and [13C] NMR copy spectra.

^{1H} NMR OF COMPOUND 45

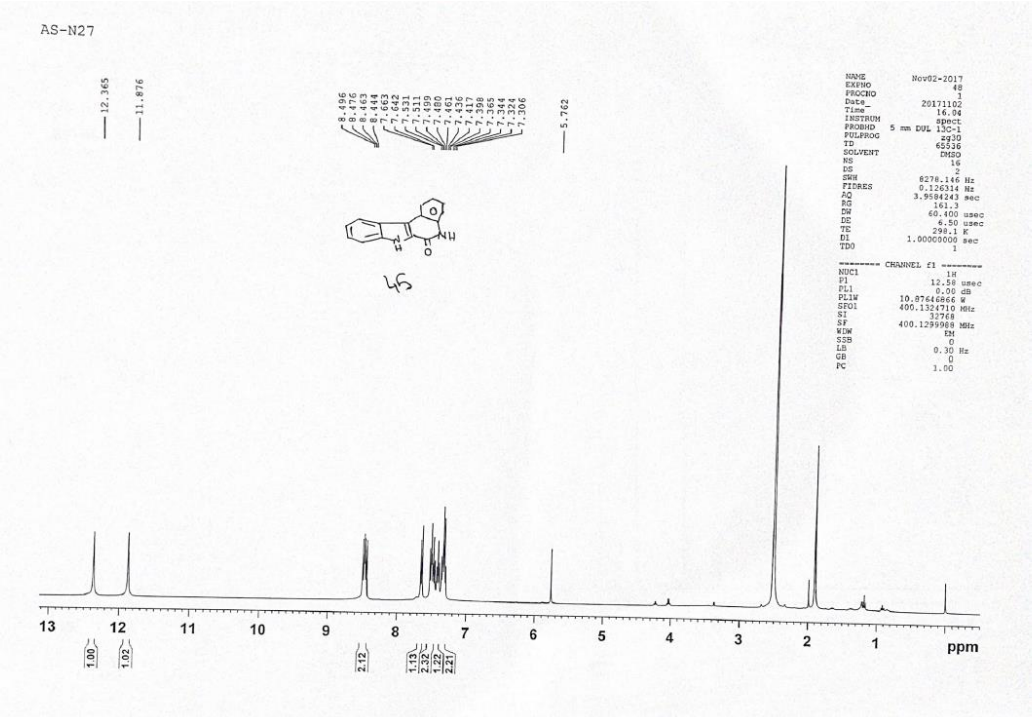

^{13}C NMR OF COMPOUND 45

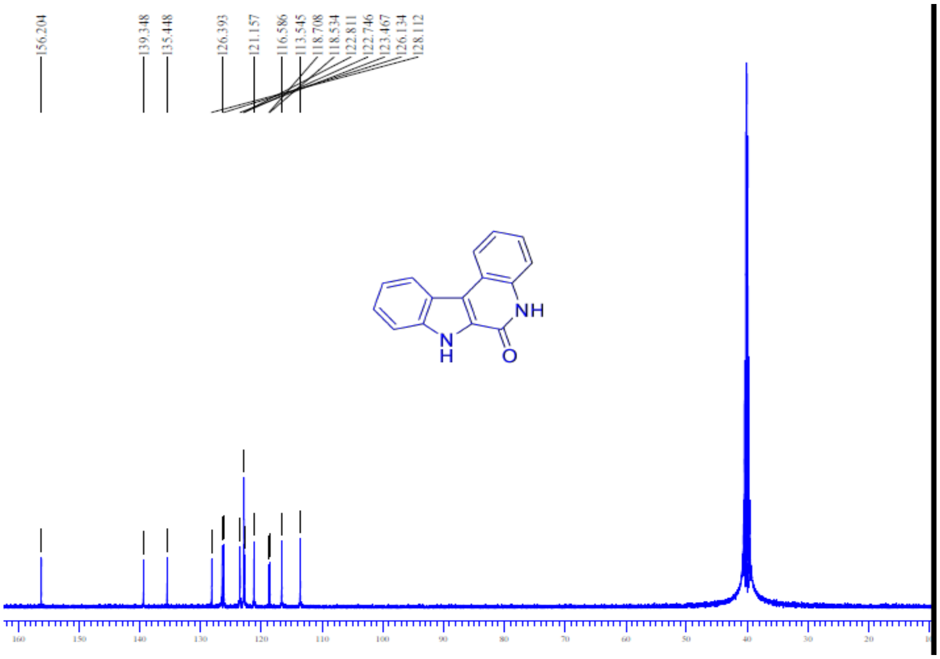

1H NMR OF COMPOUND 46

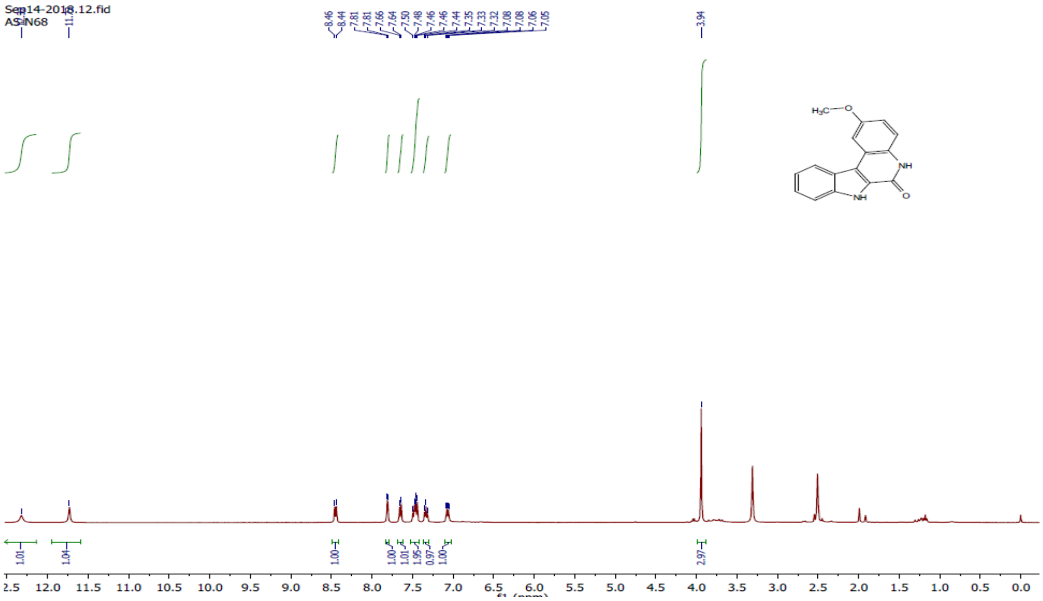

^{13}C NMR OF COMPOUND 46

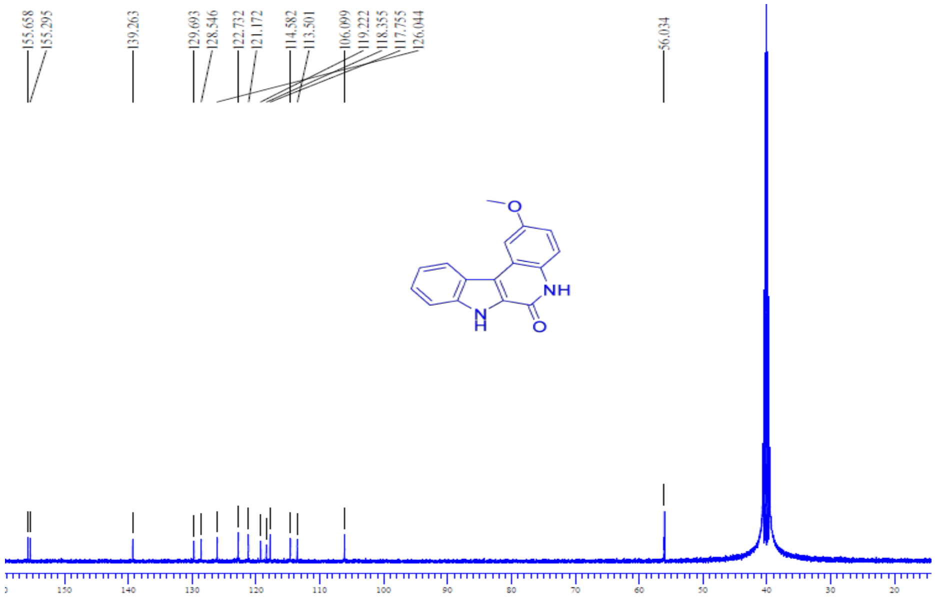

^{1}H NMR OF COMPOUND 47

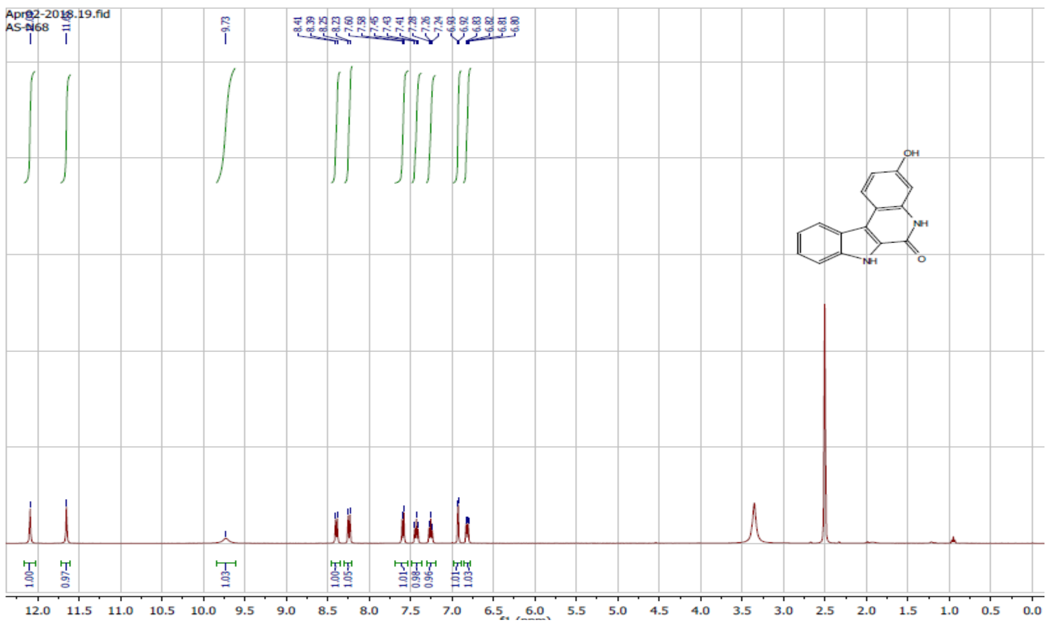

^{13}C NMR OF COMPOUND 47

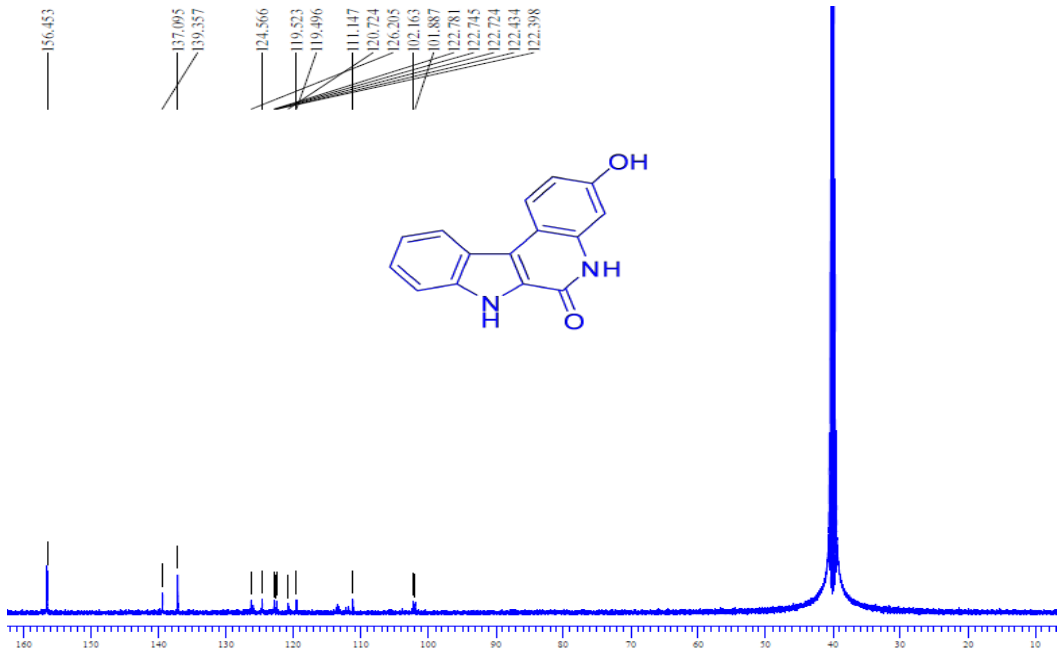

^{1}H NMR OF COMPOUND 48

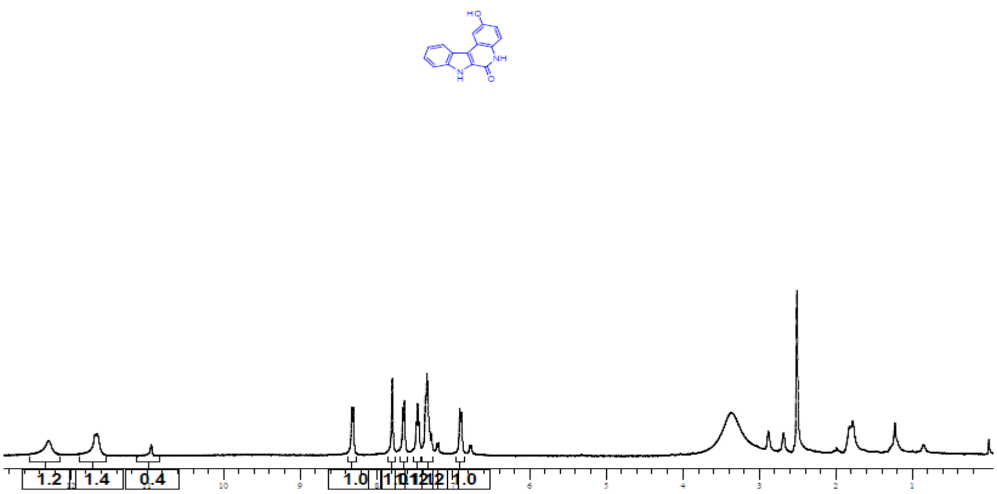

^{13}C NMR OF COMPOUND 48

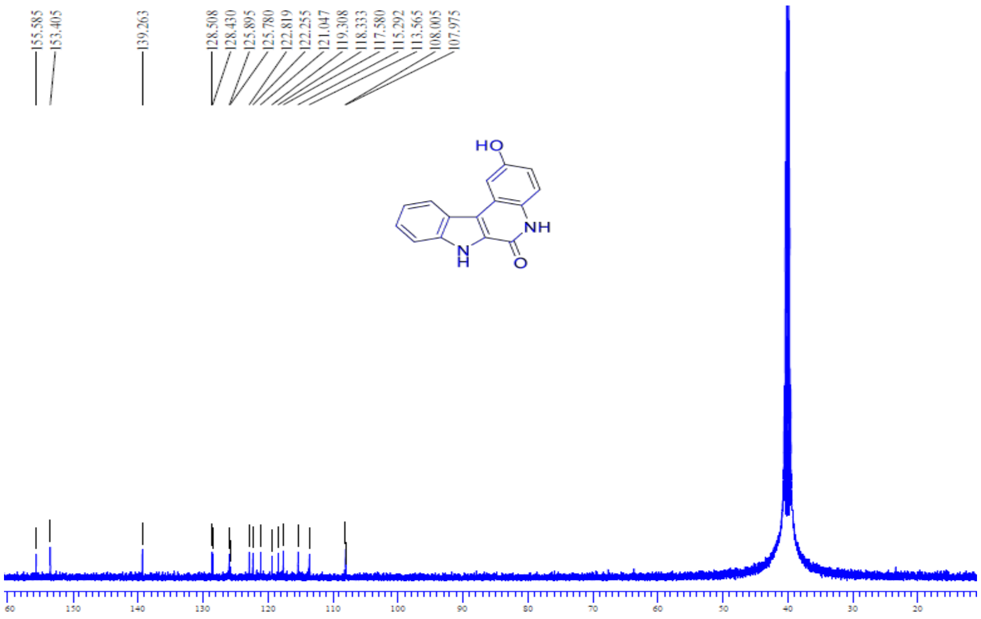

^{1}H NMR OF COMPOUND 49

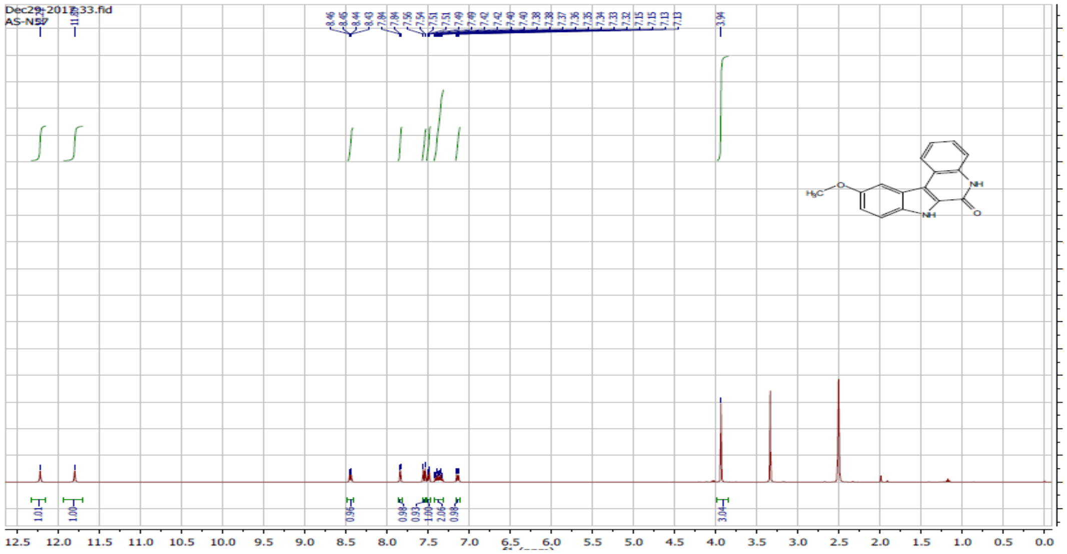

^{13}C NMR OF COMPOUND 49

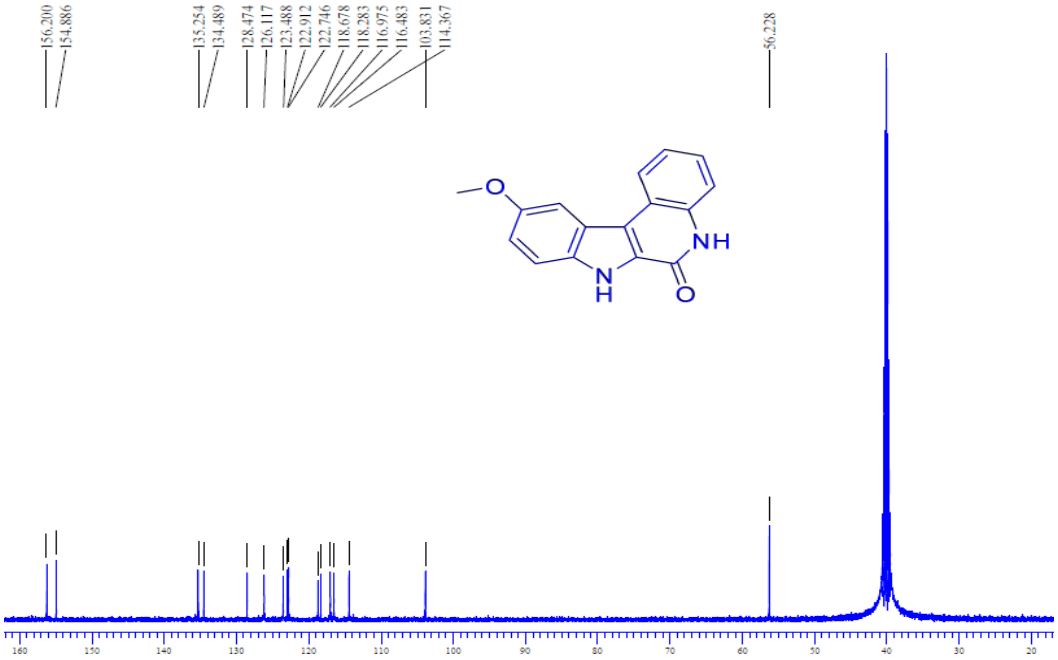

^{1}H NMR OF COMPOUND 50

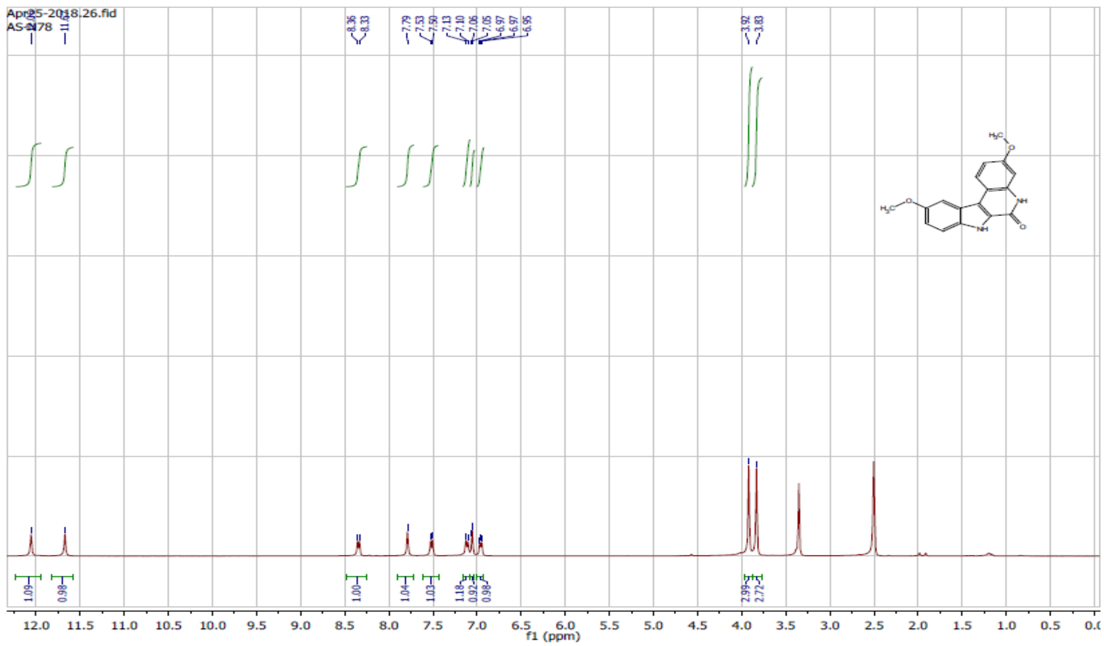

^{13}C NMR OF COMPOUND 50

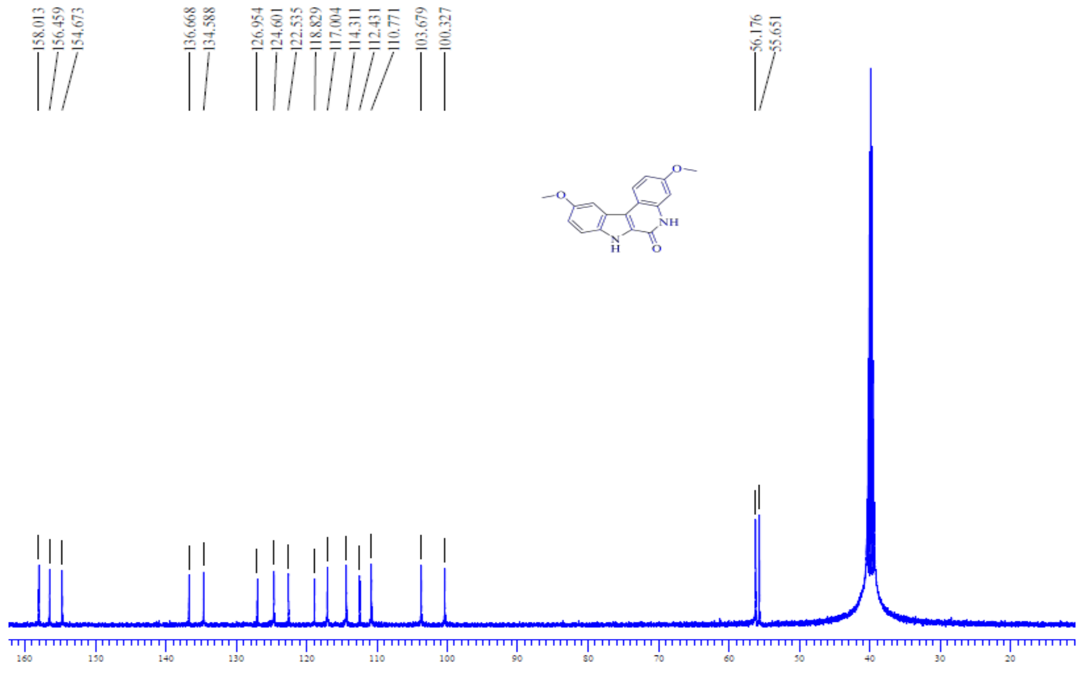

^{1}H NMR OF COMPOUND 51

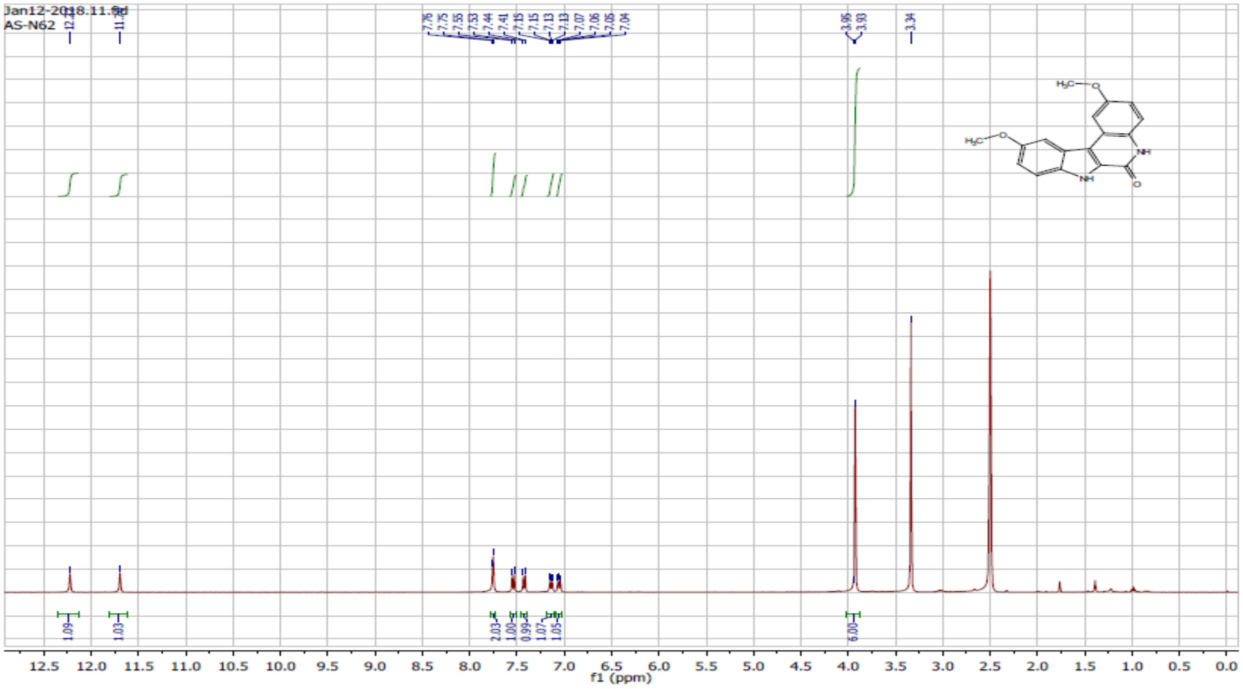

^{13}C NMR OF COMPOUND 51

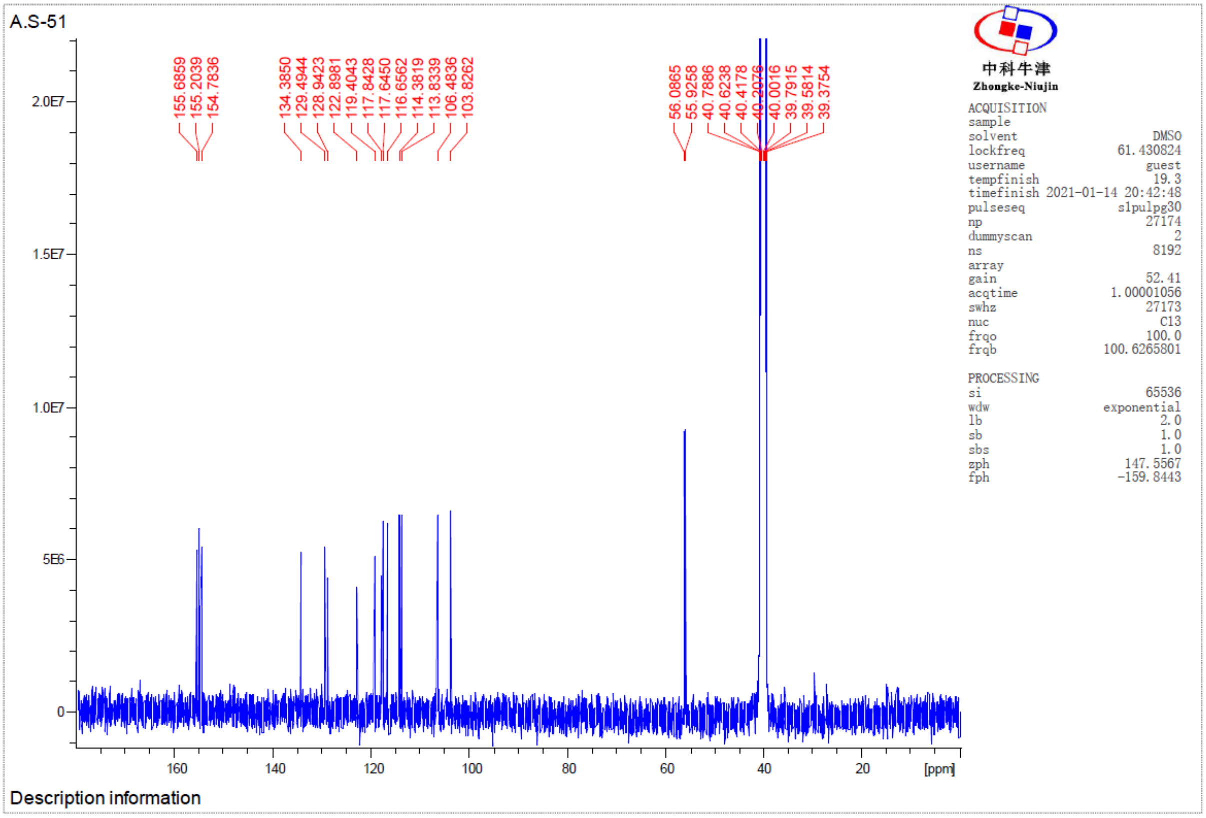

^{1}H NMR OF COMPOUND 52

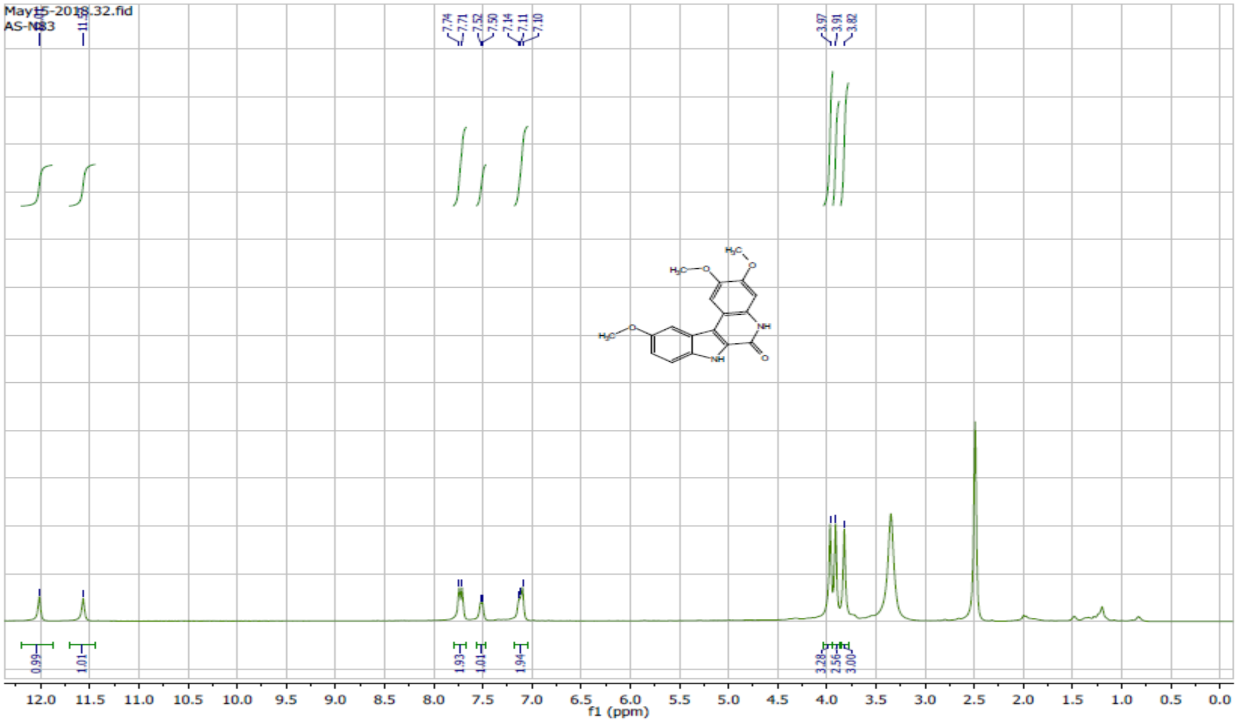

^{1}H NMR OF COMPOUND 53

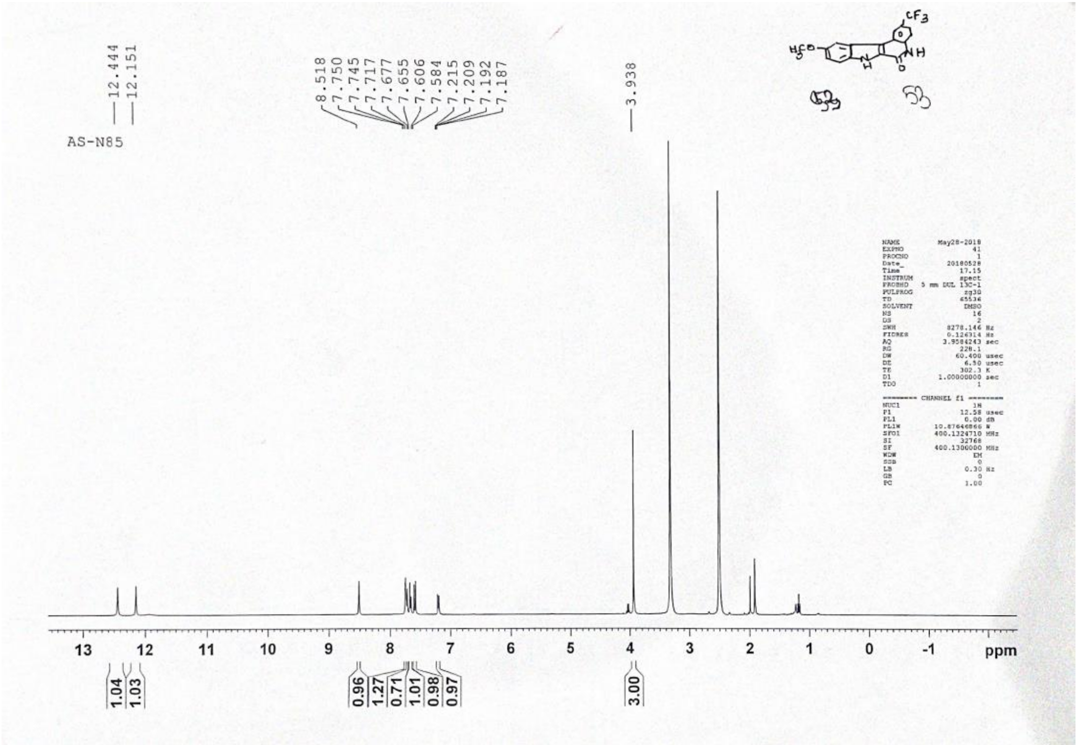

^{13}C NMR OF COMPOUND 53

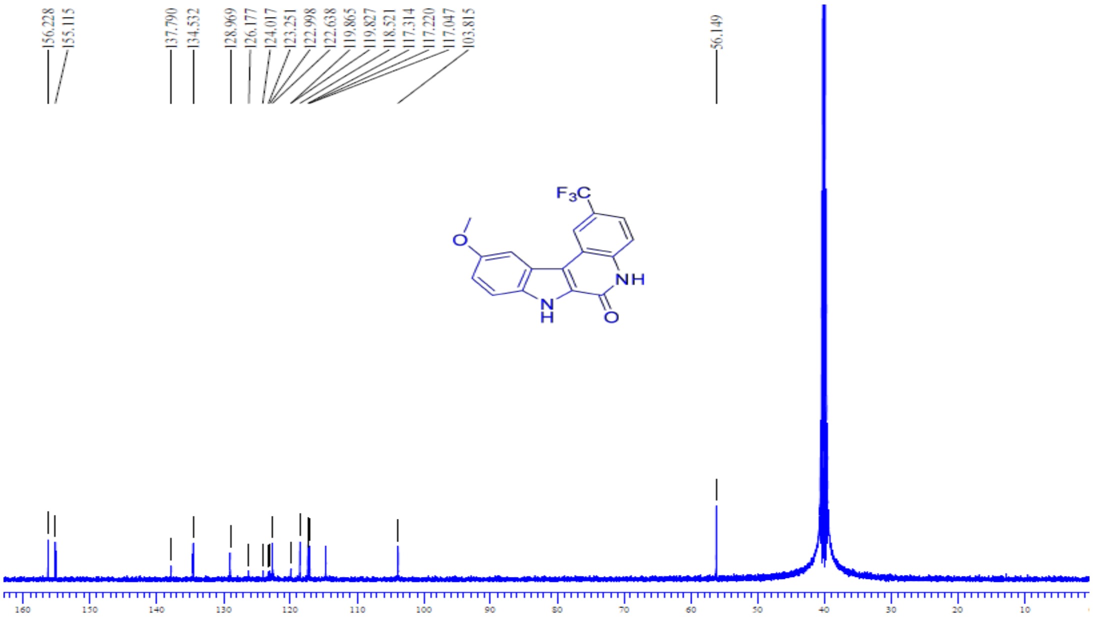

^{1}H NMR OF COMPOUND 54

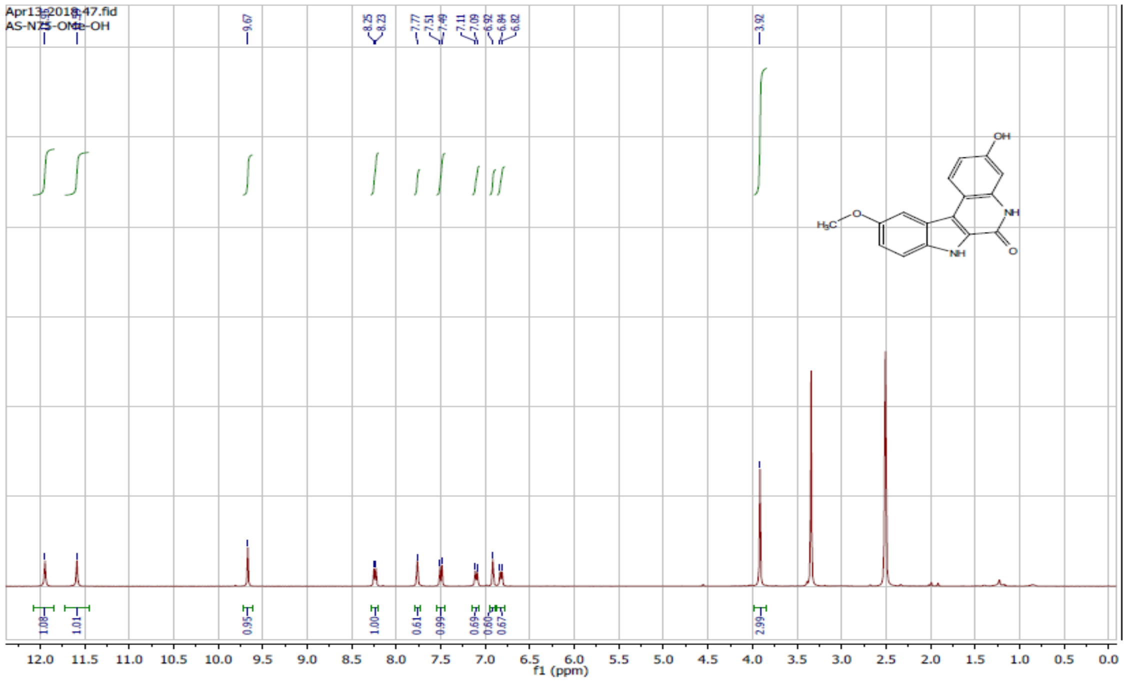

^{13}C NMR OF COMPOUND 54

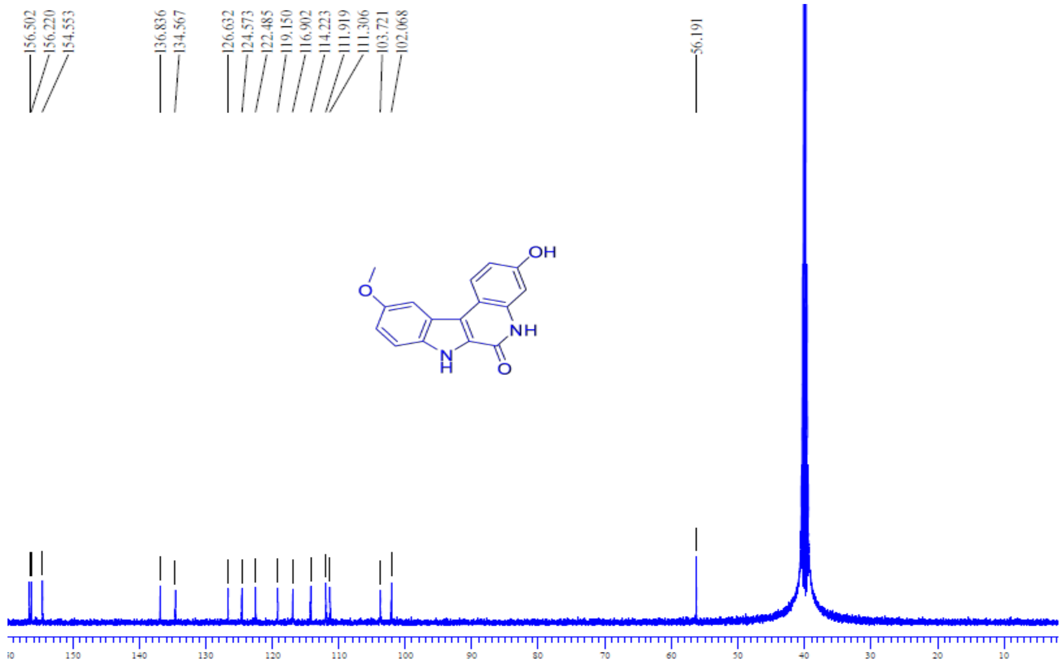

^{1}H NMR OF COMPOUND 55

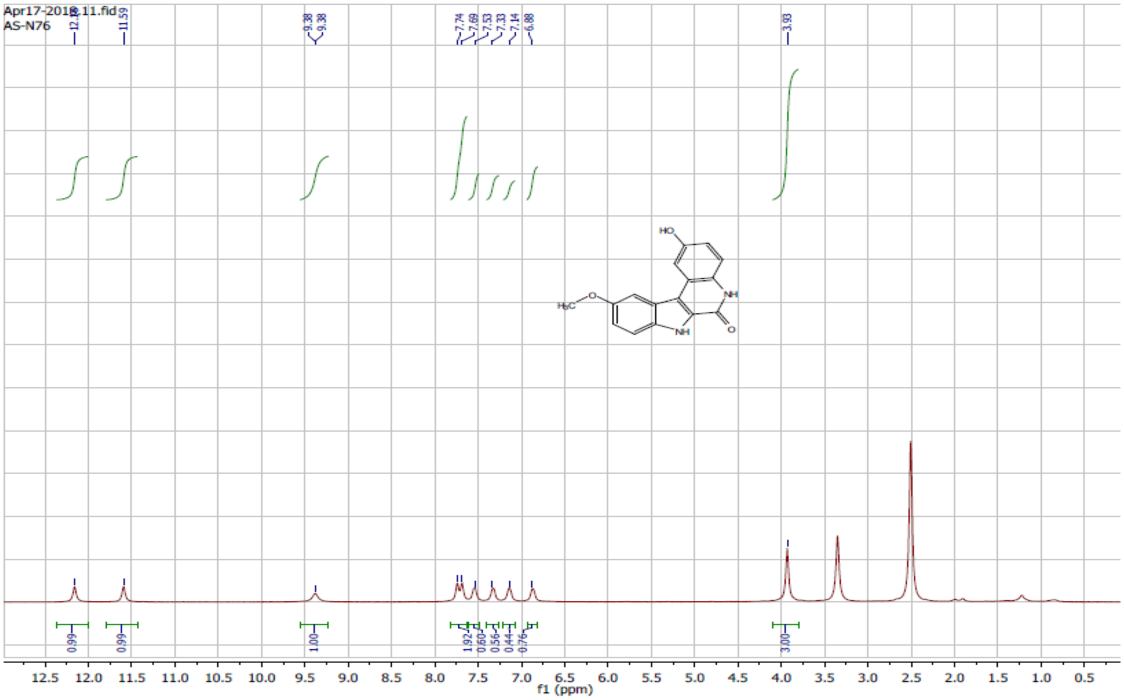

^{13}C NMR OF COMPOUND 55

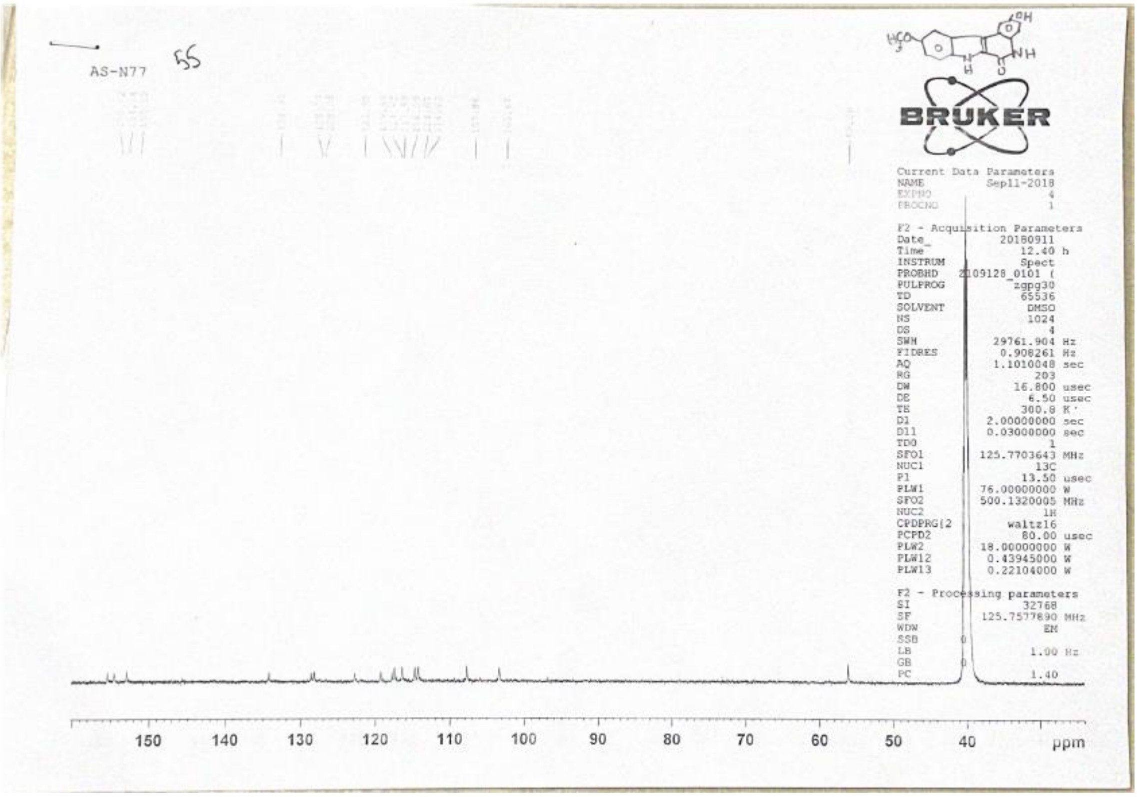

^{1}H NMR OF COMPOUND 56

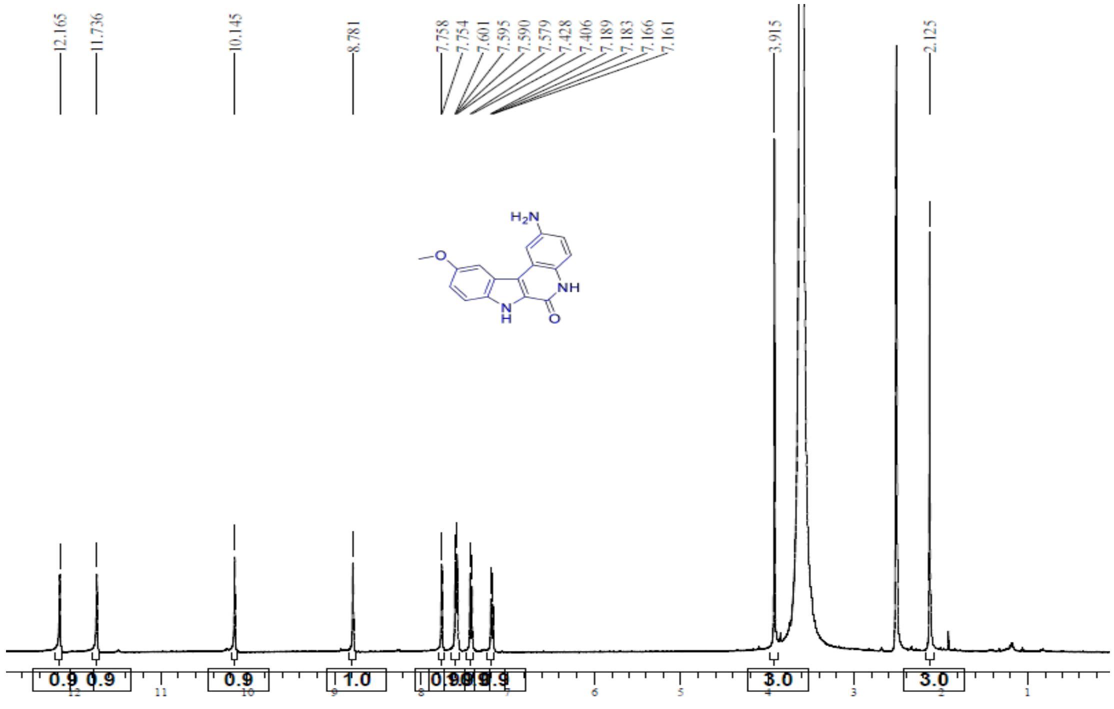

^{13}C NMR OF COMPOUND 56

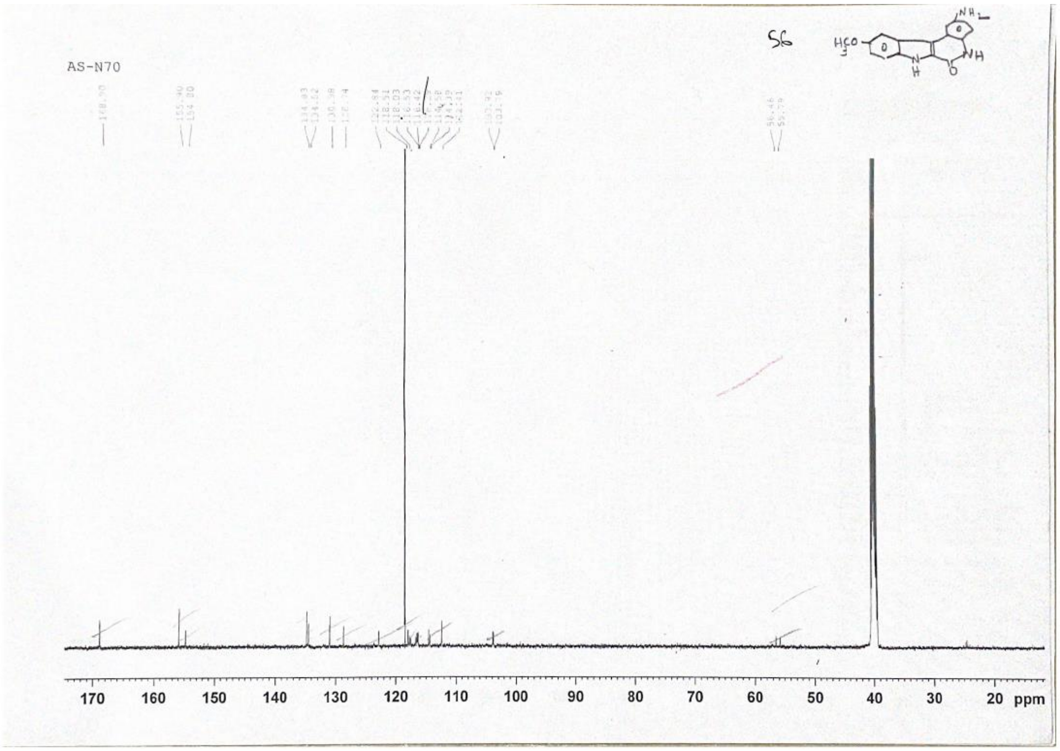

^{1}H NMR OF COMPOUND 57

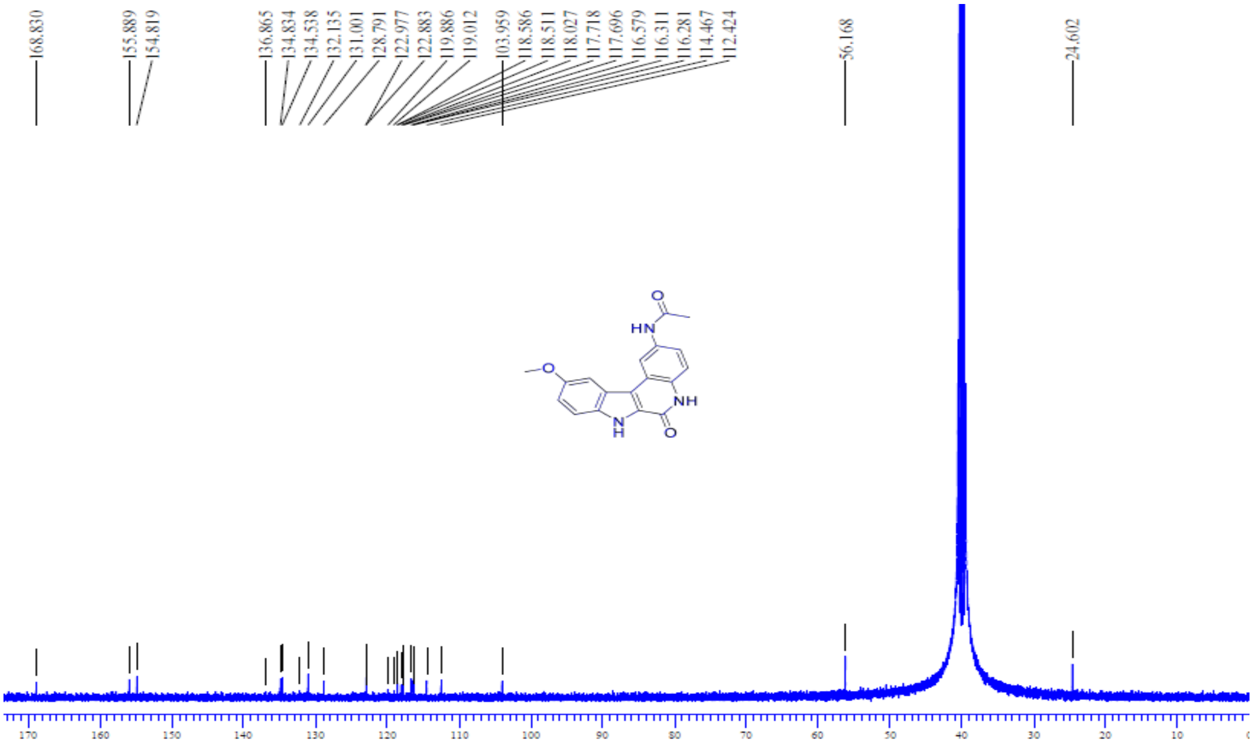

^{13}C NMR OF COMPOUND 57

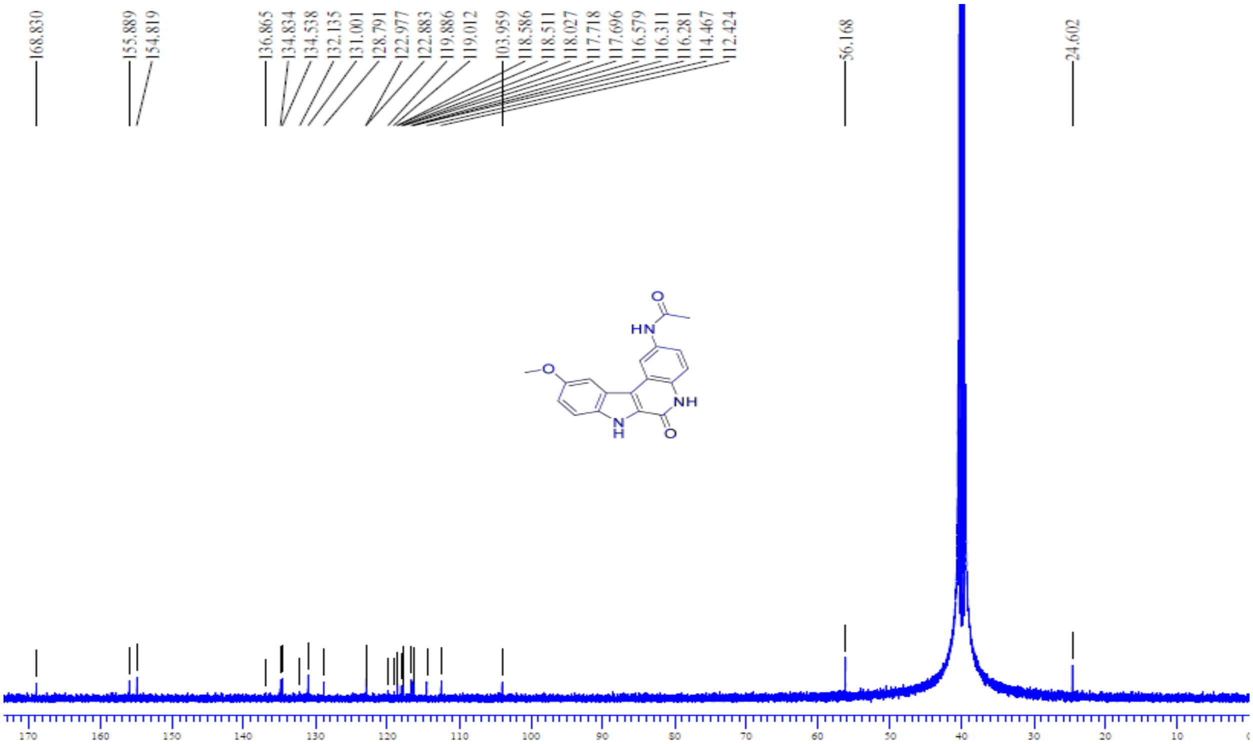

^{1}H NMR OF COMPOUND 58

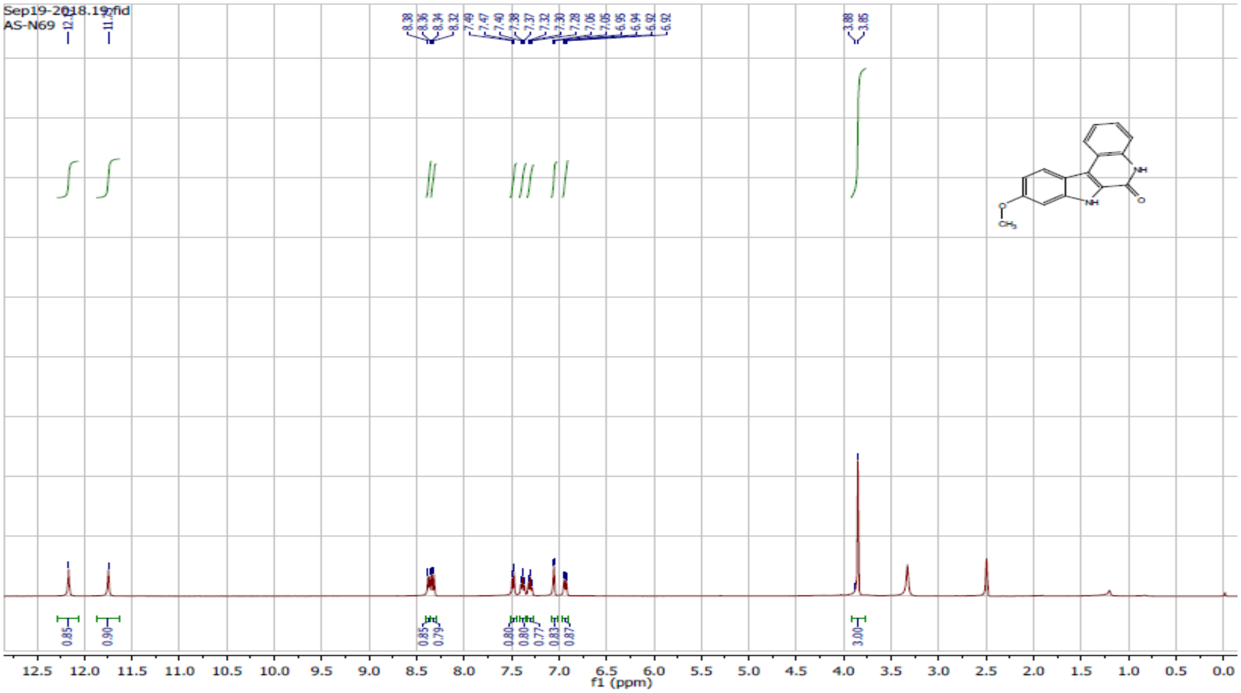

^{13}C NMR OF COMPOUND 58

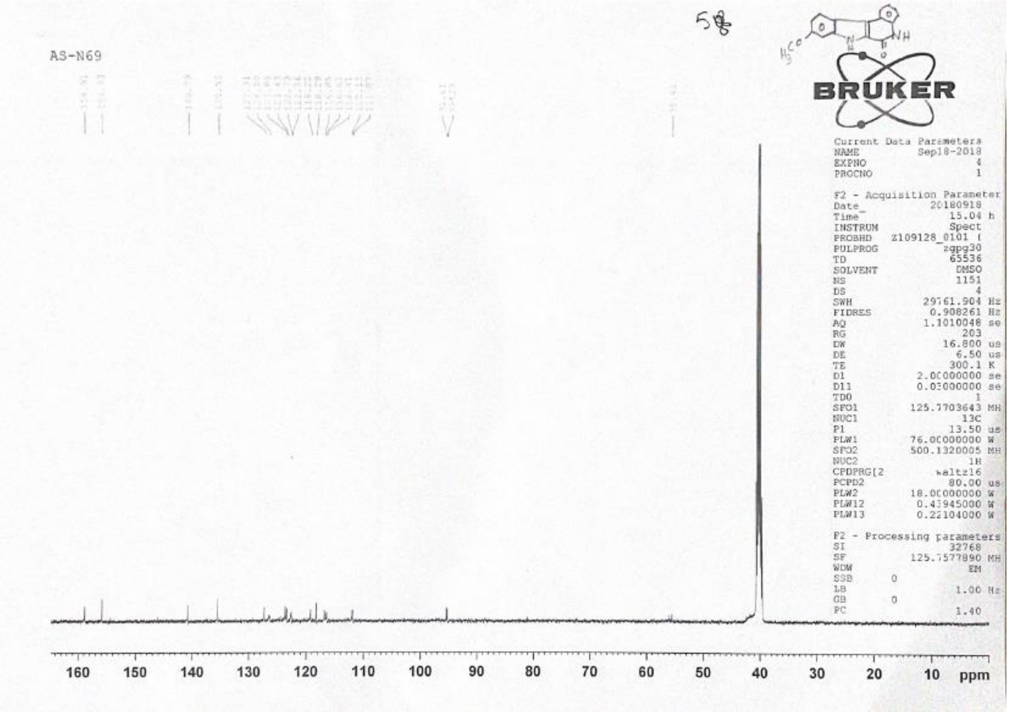

^{1}H NMR OF COMPOUND 59

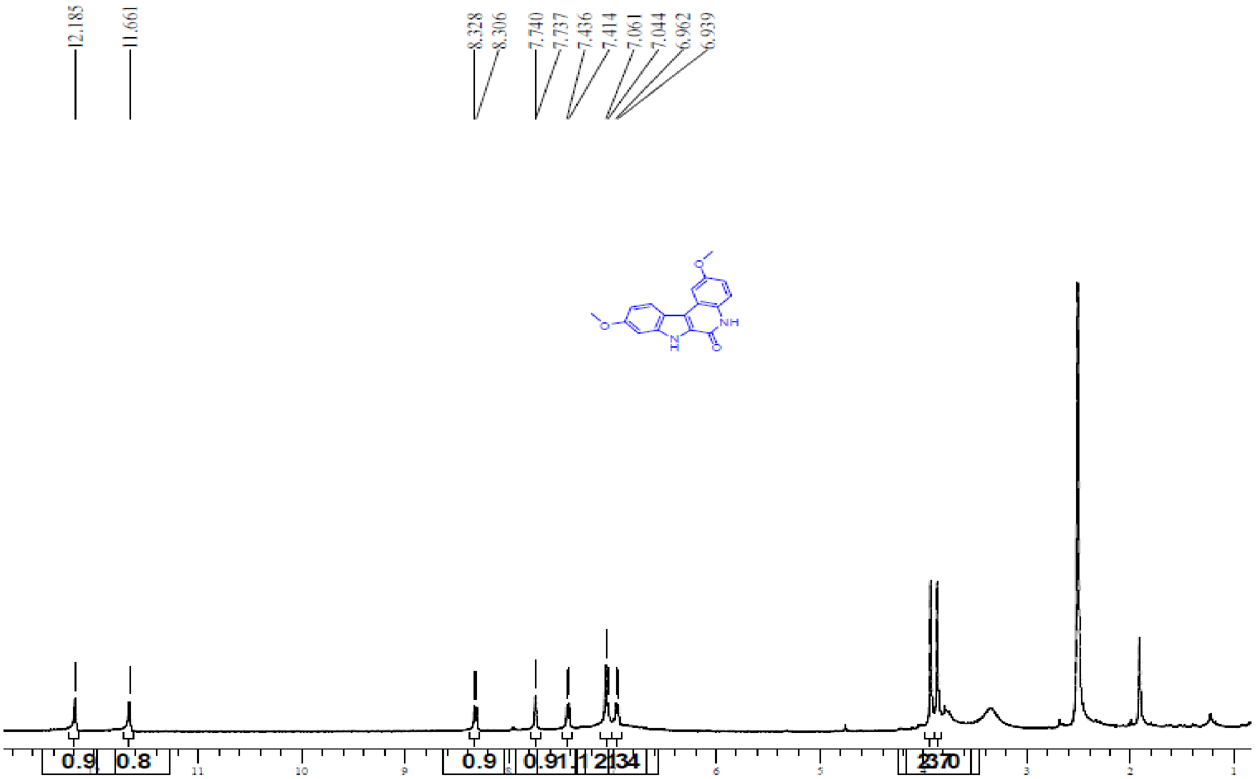

^{13}C NMR OF COMPOUND 59

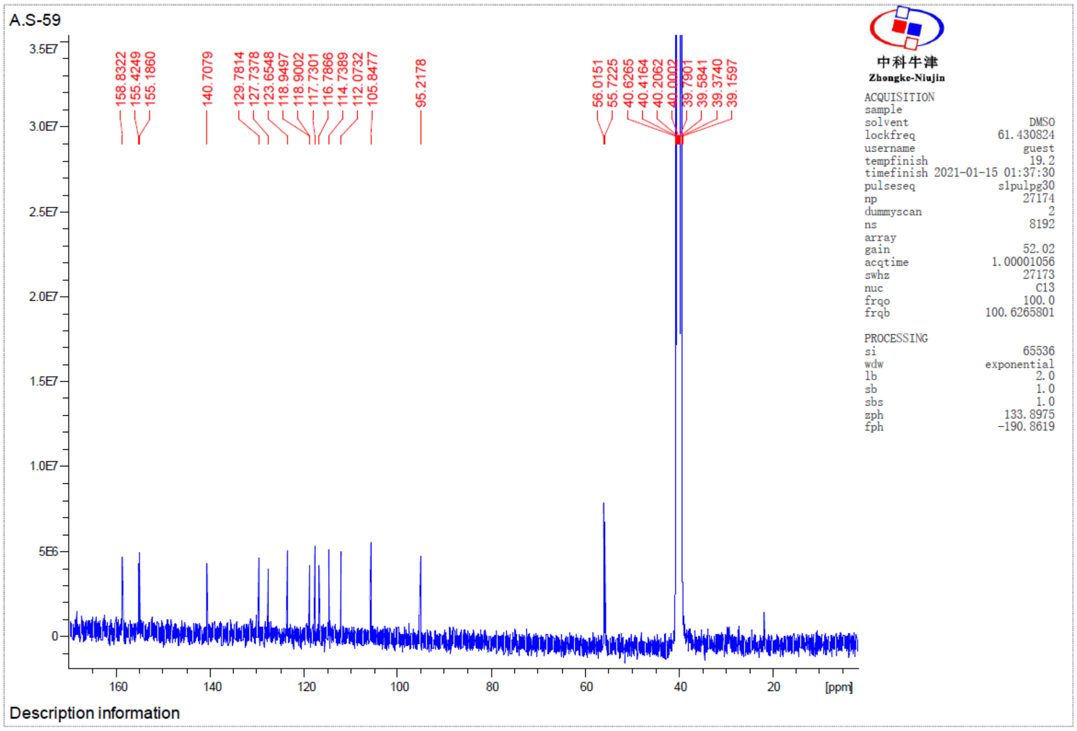

^{1}H NMR OF COMPOUND 60

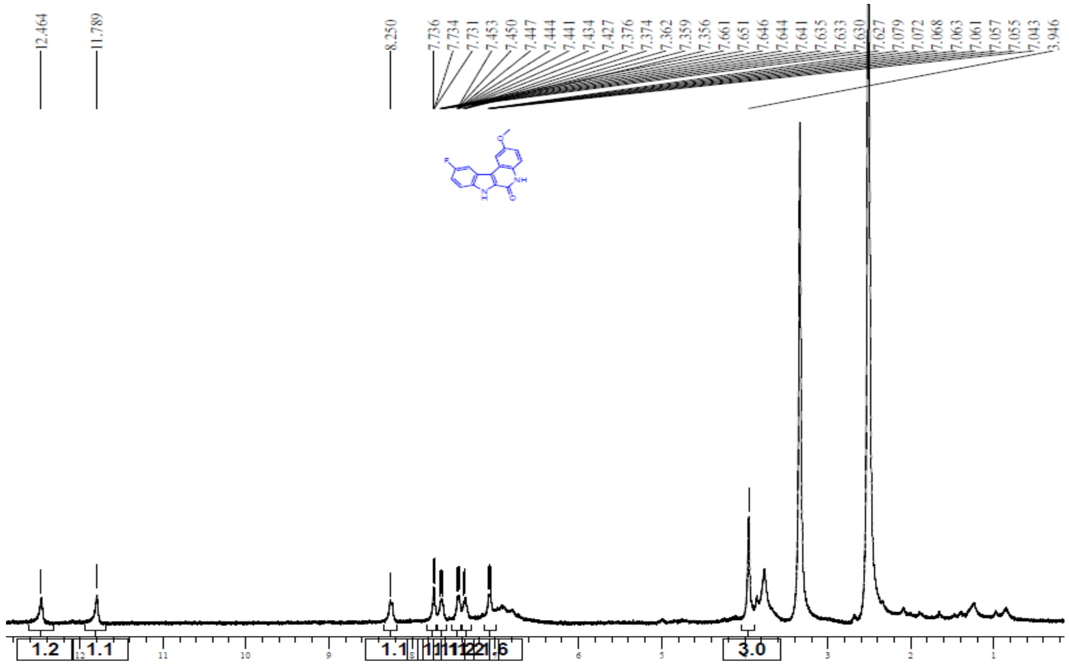

^{13}C NMR OF COMPOUND 60

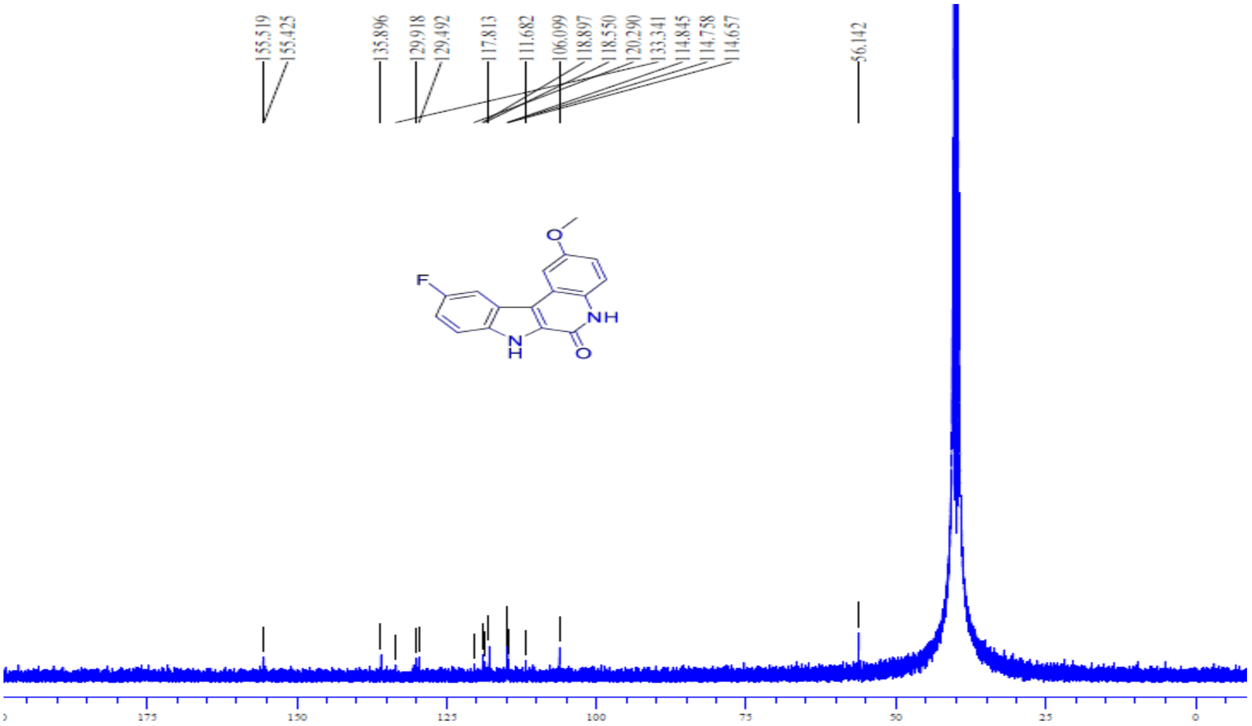

^{1}H NMR OF COMPOUND 61

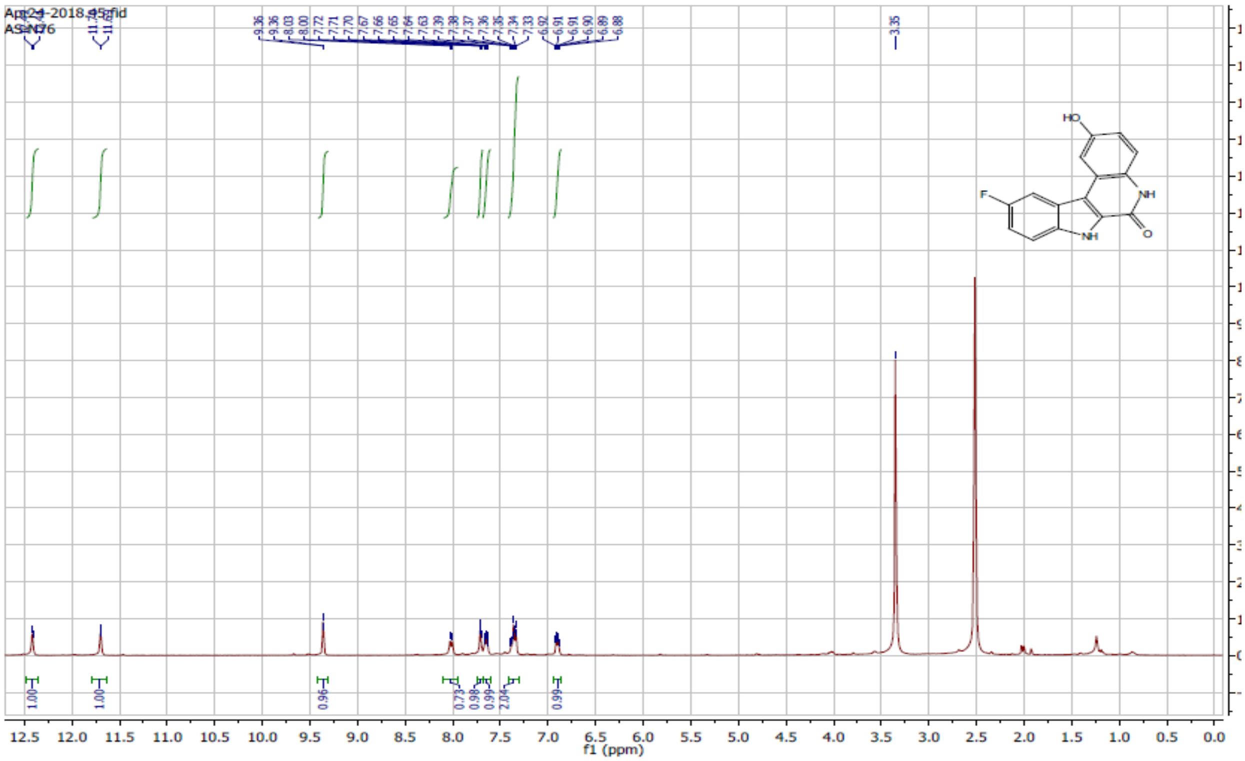

^{13}C NMR OF COMPOUND 61

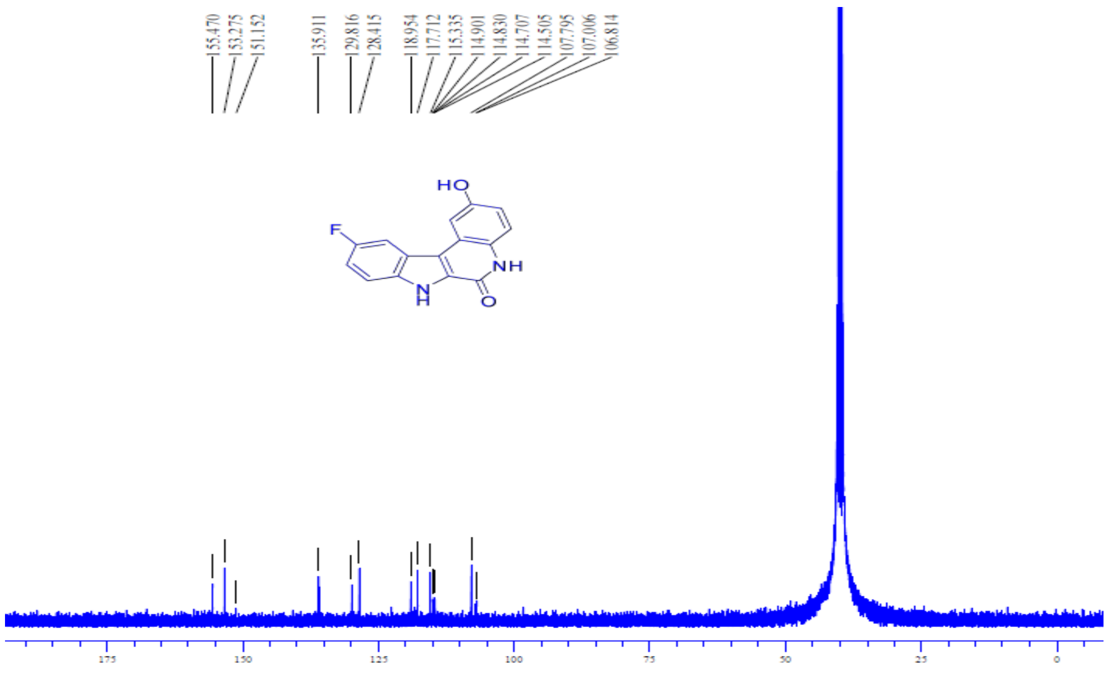

^{1}H NMR OF COMPOUND 62

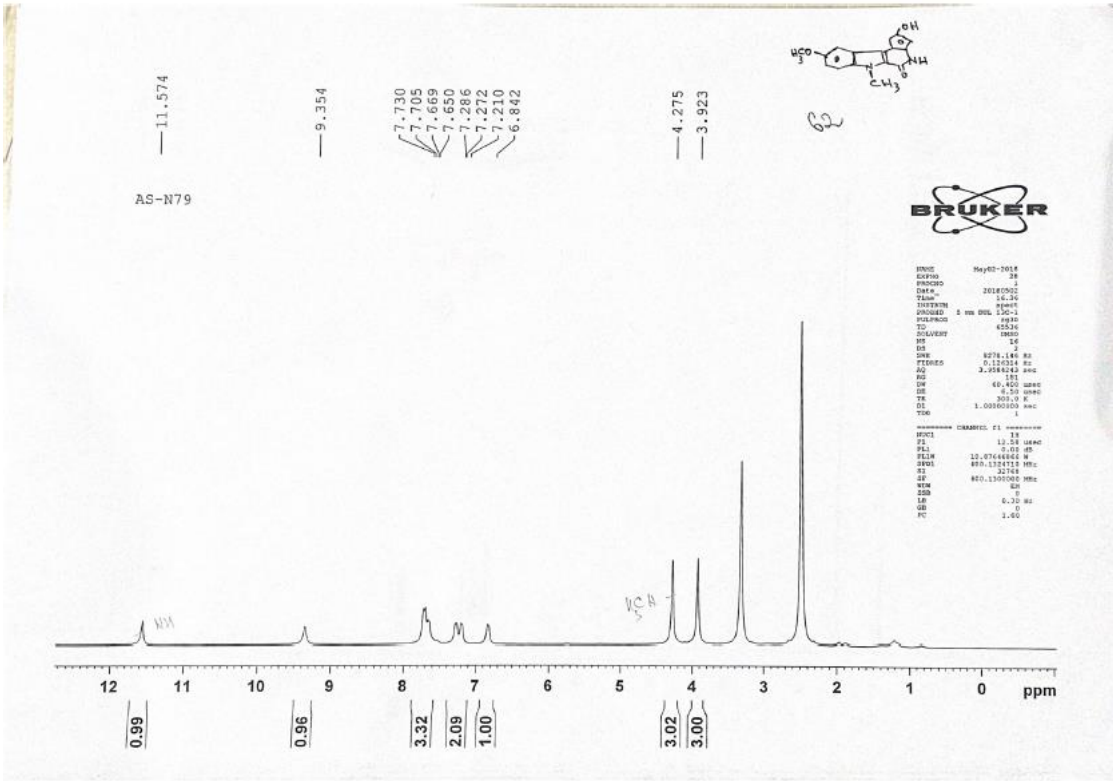

^{13}C NMR OF COMPOUND 62

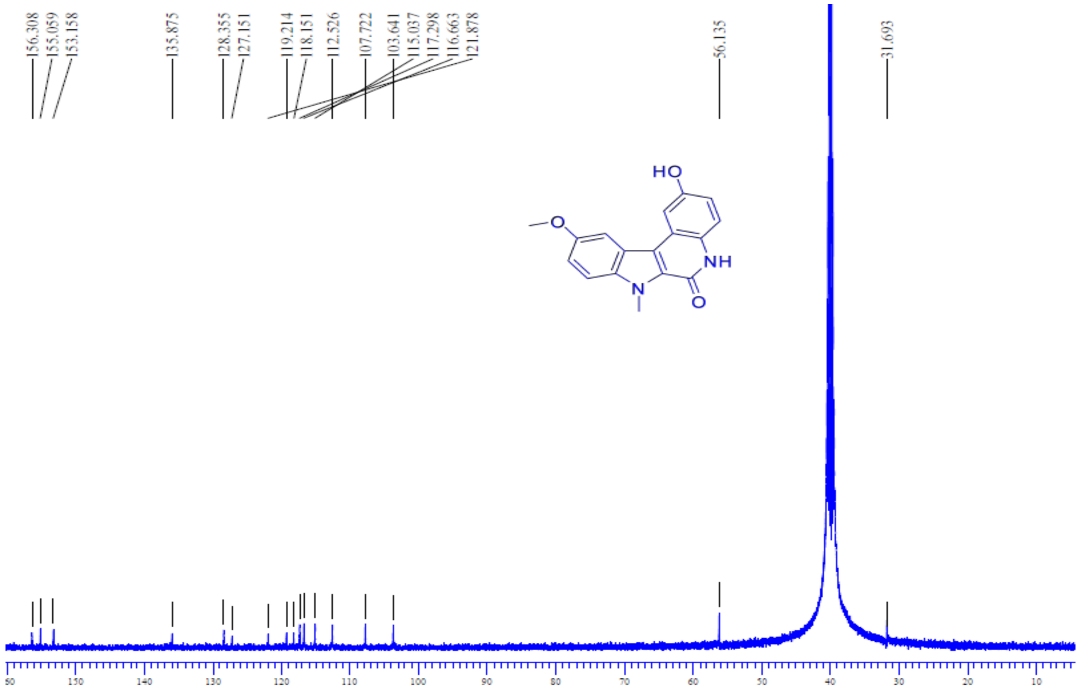

^{1}H NMR OF COMPOUND 63

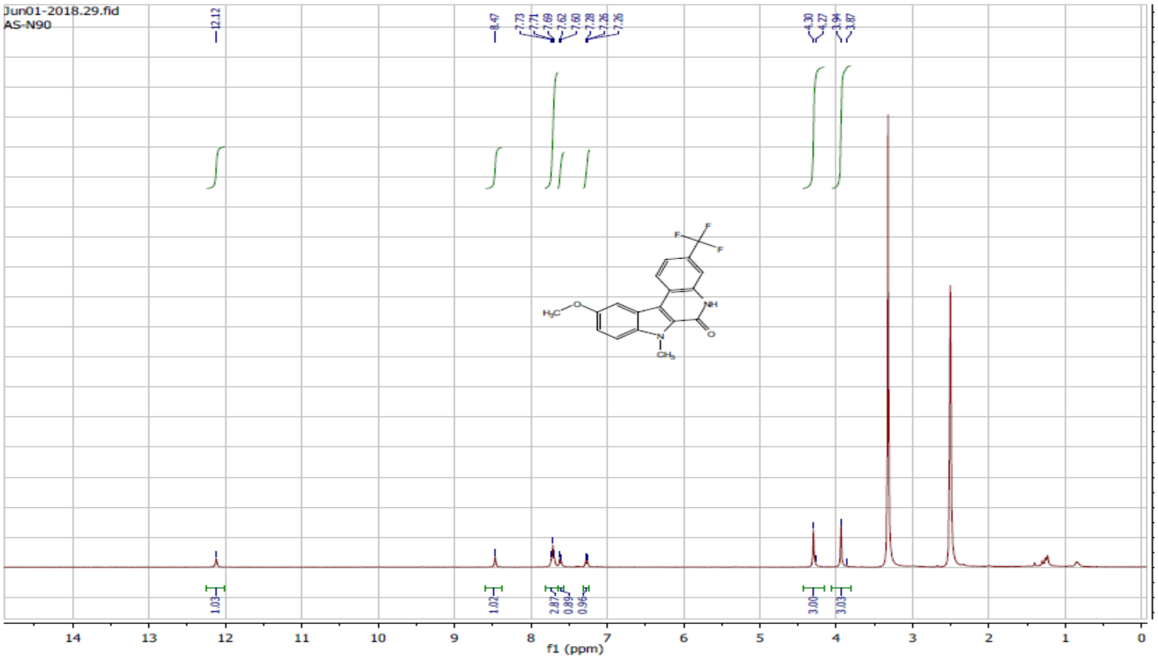

^{13}C NMR OF COMPOUND 63

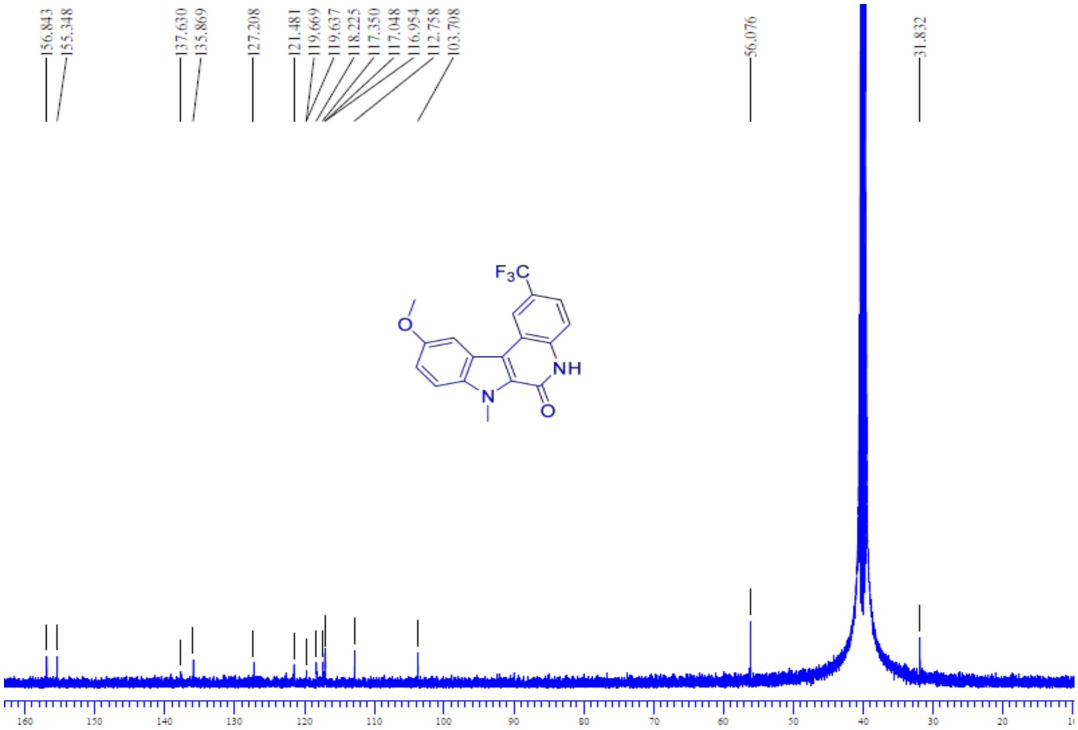

^{1}H NMR OF COMPOUND 64

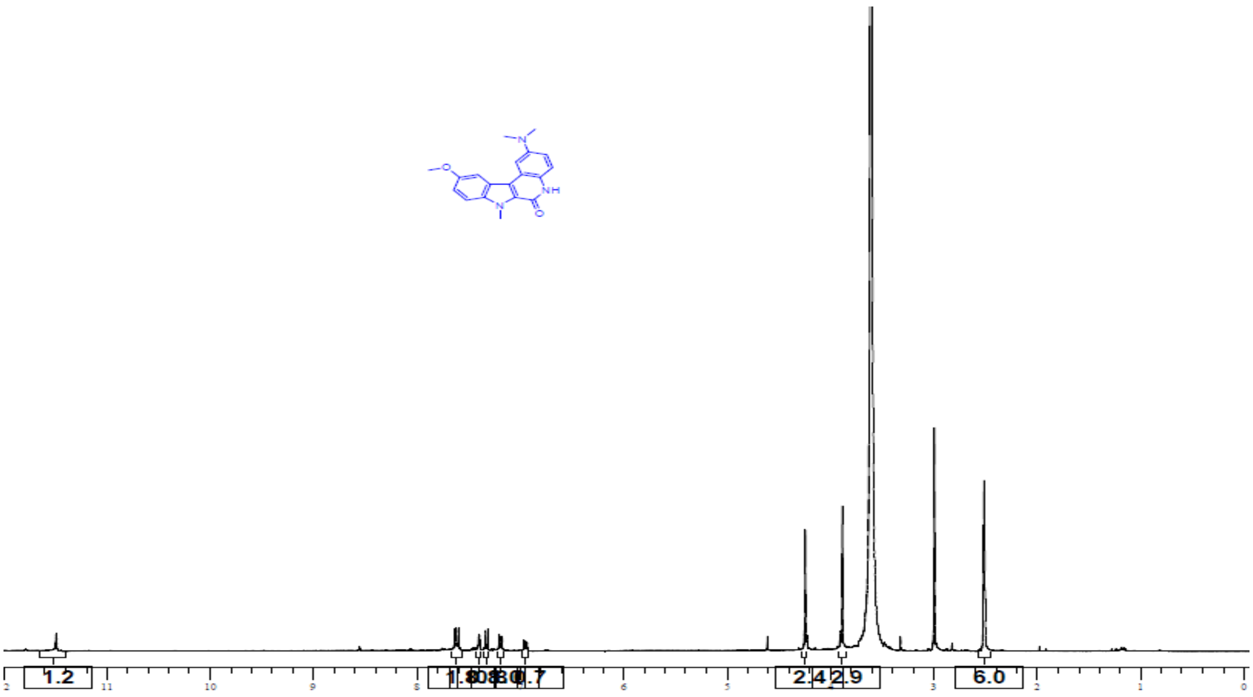

^{13}C NMR OF COMPOUND 64

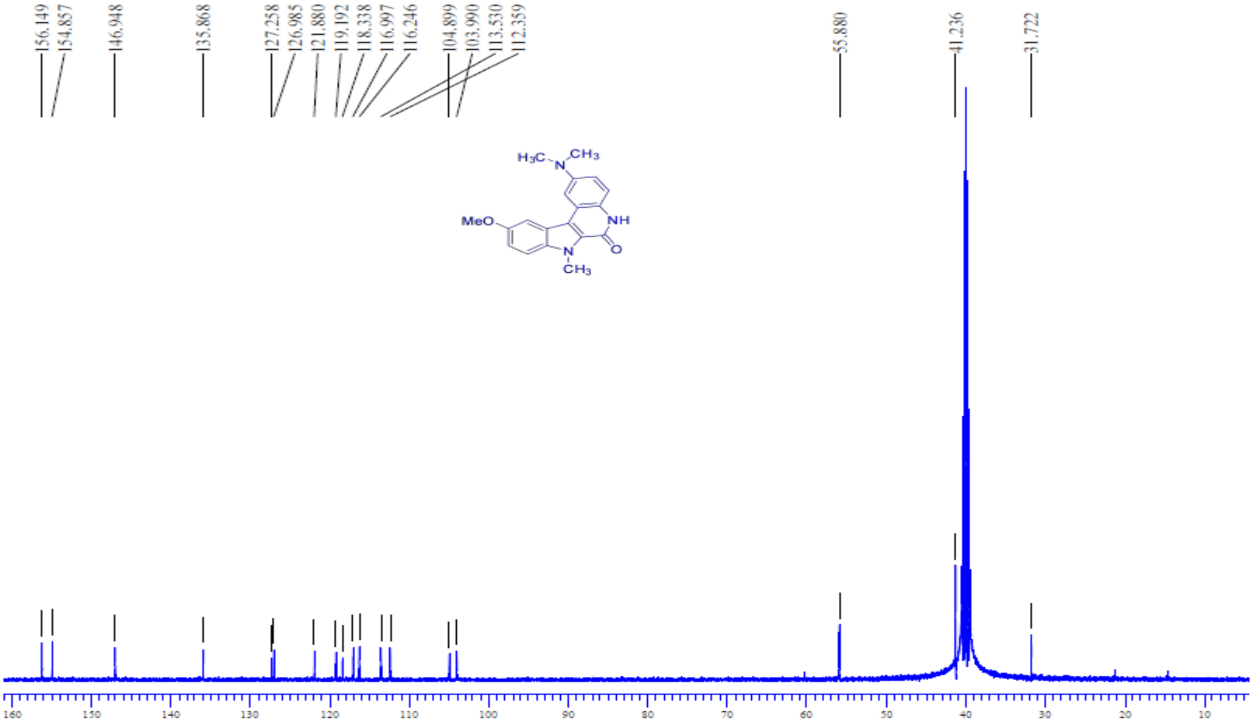

Table S1: Panel of kinases tested.

Compound 55 tested at 1 μM<br><br>*Entry*	Percentage of inhibition (%)	Protein kinase name	Assay Technology	ATP<br><br>μM
1	116	BMPR2	LanthaScreen Binding - Eu-anti-His	n.a.
2	111	MYLK4	LanthaScreen Binding - Eu-anti-GST	n.a.
3	102	TGFBR2	LanthaScreen Binding - Eu-anti-GST	n.a.
4	100	BRAF	ZLYTE	100
5	100	CLK4	LanthaScreen Binding - Eu-anti-His	n.a.
6	100	FLT3 D835Y	ZLYTE	Km
7	100	GSG2 (Haspin)	Adapta	Km
8	100	LRRK2 I2020T	Adapta	Km
9	99	STK3 (MST2)	ZLYTE	Km
10	99	STK4 (MST1)	ZLYTE	Km
11	98	CLK1	ZLYTE	Km
12	98	LRRK2 G2019S FL	Adapta	Km
13	98	MAP2K1 (MEK1)	LanthaScreen Binding - Eu-anti-His	n.a.
14	98	MAP2K1 (MEK1)	ZLYTE	100

15	98	MAP2K1 (MEK1) S218D S222D	LanthaScreen Binding - Eu-anti-His	n.a.
16	98	MAP3K7/MAP3K7IP1 (TAK1-TAB1)	LanthaScreen Binding - Eu-anti-His	n.a.
17	98	RET V804L	ZLYTE	Km
18	97	AAK1	LanthaScreen Binding - Eu-anti-GST	n.a.
19	97	CLK2	ZLYTE	Km
20	97	DYRK2	LanthaScreen Binding - Eu-anti-GST	n.a.
21	97	FLT3 ITD	LanthaScreen Binding - Eu-anti-GST	n.a.
22	97	RET	ZLYTE	Km
23	96	BRAF V599E	ZLYTE	100
24	96	DYRK1A	ZLYTE	Km
25	96	DYRK1B	ZLYTE	Km
26	96	RET S891A	ZLYTE	Km
27	96	RET Y791F	ZLYTE	Km
28	95	LRRK2 FL	Adapta	Km
28	95	LRRK2 G2019S	Adapta	Km
30	95	STK17A (DRAK1)	LanthaScreen Binding - Eu-anti-GST	n.a.
31	94	MAP2K2 (MEK2)	LanthaScreen Binding - Eu-anti-His	n.a.
32	94	RAF1 (cRAF) Y340D Y341D	ZLYTE	100

33	94	RET M918T	LanthaScreen Binding - Eu-anti-GST	n.a.
34	93	FLT3	ZLYTE	Km
35	93	LRRK2 R1441C	Adapta	Km
36	93	NTRK3 (TRKC)	ZLYTE	Km
37	93	RPS6KA6 (RSK4)	ZLYTE	Km
38	93	STK17B (DRAK2)	LanthaScreen Binding - Eu-anti-GST	Km
39	91	IRAK1	Adapta	Km
40	91	MAP3K8 (COT)	ZLYTE	100
41	91	RET G691S	LanthaScreen Binding - Eu-anti-GST	n.a.
42	90	ANKK1	LanthaScreen Binding - Eu-anti-GST	n.a.
43	90	DAPK2	LanthaScreen Binding - Eu-anti-His	n.a.
44	90	GAK	LanthaScreen Binding - Eu-anti-GST	n.a.
45	90	LRRK2 -	Adapta	Km
46	90	MAP4K5 (KHS1)	ZLYTE	Km
47	89	RPS6KA1 (RSK1)	ZLYTE	Km
48	88	RET V804M	LanthaScreen Binding - Eu-anti-GST	n.a.
49	88	RPS6KA2 (RSK3)	ZLYTE	Km
50	88	TLK2	LanthaScreen Binding - Eu-anti-GST	n.a.

51	87	MAP2K2 (MEK2)	ZLYTE	100
52	86	AMPK (A1/B2/G1)	LanthaScreen Binding - Eu-anti-His	n.a.
53	86	BLK	Zlyte	Km
54	86	DYRK3	Zlyte	Km
55	86	RET V804E	ZLYTE	Km
56	85	RET A883F	ZLYTE	Km
57	83	CDC7/DBF4	LanthaScreen Binding - Eu-anti-GST	n.a.
58	83	RPS6KA3 (RSK2)	ZLYTE	Km
59	82	AMPK (A1/B1/G3)	LanthaScreen Binding - Eu-anti-His	n.a.
60	82	GSK3A (GSK3 alpha)	ZLYTE	Km
61	82	LATS2	LanthaScreen Binding - Eu-anti-GST	n.a.
62	82	SYK	ZLYTE	Km
63	81	AMPK (A1/B1/G2)	LanthaScreen Binding - Eu-anti-His	n.a.
64	81	CSNK1D (CK1 delta)	ZLYTE	Km
65	81	FGR - ZLYTE	ZLYTE	Km
66	81	GSK3B (GSK3 beta)	ZLYTE	Km
67	81	MAP4K1 (HPK1)	LanthaScreen Binding - Eu-anti-GST	n.a.
68	81	MAP4K4 (HGK) -	ZLYTE	Km

69	80	AMPK (A2/B2/G2)	LanthaScreen Binding - Eu-anti-His	n.a.
70	80	KIT D816H	LanthaScreen Binding - Eu-anti-GST	n.a.
71	80	MAP2K5 (MEK5)	LanthaScreen Binding - Eu-anti-GST	n.a.
72	80	NTRK2 (TRKB)	ZLYTE	Km
73	79	JAK3	ZLYTE	Km
74	79	MAP3K14 (NIK)	LanthaScreen Binding - Eu-anti-GST	n.a.
75	79	PDGFRA V561D	ZLYTE	Km
76	77	ABL1 Q252H	LanthaScreen Binding - Eu-anti-GST	n.a.
77	77	IRAK3	LanthaScreen Binding - Eu-anti-GST	n.a.
78	77	KIT D816V	LanthaScreen Binding - Eu-anti-GST	n.a.
79	76	KIT A829P	LanthaScreen Binding - Eu-anti-GST	n.a.
80	74	EIF2AK2 (PKR)	LanthaScreen Binding - Eu-anti-GST	n.a.
81	74	GRK7	ZLYTE	Km
82	74	HIPK2	ZLYTE	Km
83	74	PLK2	ZLYTE	Km
84	73	CSNK1E (CK1 epsilon)	ZLYTE	Km
85	73	DDR2 T654M	LanthaScreen Binding - Eu-anti-GST	n.a.
86	72	FLT4 (VEGFR3)	ZLYTE	Km

87	71	MAP3K3 (MEKK3)	LanthaScreen Binding - Eu-anti-GST	n.a.
88	71	PIM1	ZLYTE	Km
89	70	AMPK (A2/B2/G1)	LanthaScreen Binding - Eu-anti-His	n.a.
90	70	CDK9/cyclin T1	Adapta	Km
91	70	CSNK1E (CK1 epsilon) R178C	ZLYTE	Km
92	70	PIK3CA E542K/PIK3R1 (p110 alpha E542K/p85 alpha	Adapta	10
93	69	NTRK1 (TRKA)	ZLYTE	Km
94	68	TLK1	LanthaScreen Binding - Eu-anti-GST	n.a.
95	67	AMPK (A1/B2/G2)	ZLYTE	Km
96	67	AMPK (A1/B2/G3)	ZLYTE	Km
97	67	FGFR3 K650M	LanthaScreen Binding - Eu-anti-GST	n.a.
98	67	KDR (VEGFR2)	ZLYTE	Km
99	67	LCK	ZLYTE	Km
100	67	LYN B	ZLYTE	Km
101	67	PIK3C2B	Adapta	10
102	66	MINK1	ZLYTE	Km
103	65	NUAK1 (ARK5)	Adapta	Km

104	64	CDK2/cyclin E1	LanthaScreen Binding - Eu-anti-GST	n.a.
105	63	AMPK (A2/B1/G2)	ZLYTE	Km
106	63	CSF1R (FMS)	ZLYTE	Km
107	63	DMPK	LanthaScreen Binding - Eu-anti-GST	n.a.
108	63	KIT T670E	LanthaScreen Binding - Eu-anti-GST	n.a.
109	63	MAP3K9 (MLK1)	ZLYTE	Km
110	63	STK24 (MST3)	ZLYTE	Km
111	62	CSNK1A1L	ZLYTE	Km
112	62	MLCK (MLCK2)	LanthaScreen Binding - Eu-anti-GST	
113	62	PIK3CA/PIK3R1 (p110 alpha/p85 alpha)	Adapta	Km
114	61	ABL1 H396P	LanthaScreen Binding - Eu-anti-GST	n.a.
115	61	CDK2/cyclin O	LanthaScreen Binding - Eu-anti-GST	n.a.
116	61	IRAK4	ZLYTE	Km
117	61	MST4	ZLYTE	Km
118	61	MYLK2 (skMLCK)	ZLYTE	Km
119	61	TNIK	LanthaScreen Binding - Eu-anti-GST	n.a.
120	61	YES1	ZLYTE	Km
121	60	ABL1 M351T	LanthaScreen Binding - Eu-anti-GST	n.a.

122	60	ADCK3	LanthaScreen Binding - Eu-anti-GST	n.a.
123	60	FGFR1 V561M	LanthaScreen Binding - Eu-anti-GST	n.a.
124	60	MYLK (MLCK)	LanthaScreen Binding - Eu-anti-GST	n.a.
125	60	TYRO3 (RSE)	ZLYTE	Km
126	59	CDK9 (Inactive)	LanthaScreen Binding - Eu-anti-GST	n.a.
127	59	CDK9/cyclin K	LanthaScreen Binding - Eu-anti-His	n.a.
128	59	MAP4K3 (GLK)	LanthaScreen Binding - Eu-anti-GST	n.a.
129	59	SRC N1	ZLYTE	Km
130	58	AMPK A2/B1/G1	ZLYTE	Km
131	58	AURKA (Aurora A)	ZLYTE	Km
132	58	HIPK1 (Myak)	ZLYTE	Km
133	58	MAPK15 (ERK7)	LanthaScreen Binding - Eu-anti-His	n.a.
134	57	AMPK (A2/B2/G3)	ZLYTE	Km
135	57	CLK3	ZLYTE	Km
136	57	DAPK1	Adapta	Km
137	57	ICK	LanthaScreen Binding - Eu-anti-GST	n.a.
138	57	MELK	ZLYTE	Km
139	56	FER	ZLYTE	Km

140	55	ABL1 Y253F	ZLYTE	Km
141	55	IKBKB (IKK beta)	ZLYTE	Km
142	54	DNA-PK	ZLYTE	Km
143	54	JAK2	ZLYTE	Km
144	54	VRK2	LanthaScreen Binding - Eu-anti-GST	n.a.
145	53	MYO3B (MYO3 beta)	LanthaScreen Binding - Eu-anti-GST	n.a.
146	53	NUAK2	LanthaScreen Binding - Eu-anti-GST	n.a.
147	53	PIK3CA/PIK3R3 (p110 alpha/p55 gamma)	Adapta	Km
148	52	INSRR (IRR)	ZLYTE	Km
149	51	CSNK1A1 (CK1 alpha 1)	ZLYTE	Km
150	51	PIK3CA E545K/PIK3R1 (p110 alpha E545K/p85 alpha)	Adapta	Km
151	51	STK16 (PKL12)	LanthaScreen Binding - Eu-anti-His	n.a.
152	51	TYK2	ZLYTE	Km
153	50	CAMK2D (CaMKII delta)	ZLYTE	Km
154	50	CDK3/cyclin E1	LanthaScreen Binding - Eu-anti-GST	n.a.
155	50	GRK1	LanthaScreen Binding - Eu-anti-GST	n.a.
156	50	MASTL	LanthaScreen Binding - Eu-anti-GST	n.a.

157	50	PEAK1	ZLYTE	Km
158	50	PLK4	LanthaScreen Binding - Eu-anti-GST	n.a.
159	49	EPHB1	ZLYTE	Km
160	49	FYN	ZLYTE	Km
161	49	ITK	ZLYTE	Km
162	49	LYN A	ZLYTE	Km
163	49	MAP3K19 (YSK4)	ZLYTE	Km
164	49	PASK	ZLYTE	Km
165	49	PDGFRA D842V	ZLYTE	Km
167	49	PIP5K1C	Adapta	10
168	49	TBK1	ZLYTE	Km
169	48	ABL1 G250E	ZLYTE	Km
170	47	AMPK A1/B1/G1	ZLYTE	Km
171	47	MAP4K2 (GCK)	ZLYTE	Km
172	47	PIK3C2G (PI3K-C2 gamma)	Adapta	Km
173	46	FGFR3 V555M	ZLYTE	Km
174	46	PTK2B (FAK2)	ZLYTE	Km
175	46	STK33	LanthaScreen Binding - Eu-anti-His	n.a.

176	45	BRSK1 (SAD1)	ZLYTE	Km
177	45	FYN A	LanthaScreen Binding - Eu-anti-GST	n.a.
178	45	KIT N822K	LanthaScreen Binding - Eu-anti-GST	n.a.
179	45	PRKD1 (PKC mu)	ZLYTE	Km
180	45	ULK3	LanthaScreen Binding - Eu-anti-His	n.a.
181	44	DAPK3 (ZIPK)	ZLYTE	Km
182	44	MERTK	ZLYTE	Km
183	44	PIK3CD/PIK3R1 (p110 delta/p85 alpha)	Adapta	Km
184	43	ABL1	ZLYTE	Km
185	43	CDK2/cyclin A1	LanthaScreen Binding - Eu-anti-GST	n.a.
186	43	FGFR3 K650E	ZLYTE	Km
187	43	MAPK8 (JNK1)	LanthaScreen Binding - Eu-anti-His	n.a.
188	43	PDGFRA (PDGFR alpha)	ZLYTE	Km
189	42	AMPK (A2/B1/G3)	ZLYTE	Km
190	42	DYRK4	ZLYTE	Km
191	42	FGFR2	ZLYTE	Km
192	42	GRK4	ZLYTE	Km
193	42	MAP3K2 (MEKK2)	LanthaScreen Binding - Eu-anti-GST	n.a.

194	42	TTK	LanthaScreen Binding - Eu-anti-GST	n.a.
195	41	AURKB (Aurora B)	ZLYTE	Km
196	41	CAMK4 (CaMKIV)	ZLYTE	Km
197	41	DDR2 N456S	LanthaScreen Binding - Eu-anti-GST	n.a.
198	41	PI4KB (PI4K beta)	Adapta	Km
199	40	CDK8/cyclin C	LanthaScreen Binding - Eu-anti-His	n.a.
200	40	EPHA2	ZLYTE	Km
201	40	PRKCN (PKD3)	ZLYTE	Km
202	40	SLK	LanthaScreen Binding - Eu-anti-GST	n.a.
203	39	ABL1 E255K	ZLYTE	Km
204	39	PRKD2 (PKD2)	ZLYTE	Km
205	39	RPS6KA4 (MSK2)	ZLYTE	Km
206	38	CDK16 (PCTK1)/cyclin Y	LanthaScreen Binding - Eu-anti-GST	n.a.
207	38	CHUK (IKK alpha)	Adapta	Km
208	38	FGFR1	ZLYTE	Km
209	38	HCK	ZLYTE	Km
210	37	AXL	ZLYTE	Km
211	37	CDK11/cyclin C	LanthaScreen Binding - Eu-anti-GST	n.a.

212	37	KIT V559D	ZLYTE	Km
213	37	PRKG2 (PKG2)	ZLYTE	Km
214	37	SRC	ZLYTE	Km
215	36	CDK18/cyclin Y -	ZLYTE	Km
216	36	JAK2 JH1 JH2 V617F	ZLYTE	Km
217	36	MAP3K11 (MLK3)	LanthaScreen Binding - Eu-anti-GST	n.a.
218	35	AXL R499C - LanthaScreen Binding	LanthaScreen Binding - Eu-anti-GST	n.a.
219	35	CDK1/cyclin B	ZLYTE	Km
220	35	CDK14 (PFTK1)/cyclin Y	LanthaScreen Binding - Eu-anti-GST	n.a.
221	35	FGFR3 G697C	LanthaScreen Binding - Eu-anti-GST	n.a.
222	35	HIPK3 (YAK1)	ZLYTE	Km
223	35	PRKACB (PRKAC beta)	LanthaScreen Binding - Eu-anti-GST	n.a.
224	35	TAOK1	LanthaScreen Binding - Eu-anti-GST	n.a.
225	34	ALK C1156Y	LanthaScreen Binding - Eu-anti-GST	n.a.
226	34	CDK2/cyclin A	ZLYTE	Km
227	34	EGFR (ErbB1) d746-750	LanthaScreen Binding - Eu-anti-GST	n.a.
228	34	EGFR (ErbB1) d747-749 A750P	LanthaScreen Binding - Eu-anti-GST	n.a.

229	33	ALK F1174L	ALanthaScreen Binding - Eu-anti-GST	n.a.
230	33	CDK5/p25	ZLYTE	Km
231	33	CHEK2 (CHK2)	ZLYTE	Km
232	33	CSNK1G2 (CK1 gamma 2)	ZLYTE	Km
233	33	EPHA1	ZLYTE	Km
234	33	FRK (PTK5)	ZLYTE	Km
235	33	JAK2 JH1 JH2	ZLYTE	Km
236	33	MYO3A (MYO3 alpha)	LanthaScreen Binding - Eu-anti-GST	n.a.
237	33	PIK3CB/PIK3R2 (p110 beta/p85 beta)	Adapta	Km
238	33	PIM3	ZLYTE	Km
239	33	PIP5K1B	Adapta	10
240	32	ALK T1151_L1152insT	LanthaScreen Binding - Eu-anti-GST	n.a.
241	32	CDK5/p35	ZLYTE	Km
242	32	GRK6	ZLYTE	Km
243	32	KIT V559D T670I	LanthaScreen Binding - Eu-anti-GST	n.a.
244	32	PHKG1	ZLYTE	Km
245	32	PKN2 (PRK2)	LanthaScreen Binding - Eu-anti-GST	n.a.
246	31	MARK3	ZLYTE	Km

247	31	MARK4	ZLYTE	Km
248	31	MERTK (cMER) A708S	LanthaScreen Binding - Eu-anti-GST	n.a.
249	31	ULK1	LanthaScreen Binding - Eu-anti-GST	n.a.
250	30	CAMK2G (CaMKII gamma)	LanthaScreen Binding - Eu-anti-GST	n.a.
251	30	EGFR (ErbB1) T790M	ZLYTE	Km
252	30	KIT Y823D	LanthaScreen Binding - Eu-anti-GST	n.a.
253	30	TNK1	ZLYTE	Km
254	29	MAPK10 (JNK3)	LanthaScreen Binding - Eu-anti-GST	
255	29	ROS1	ZLYTE	Km
256	29	SGK (SGK1)	ZLYTE	Km
257	28	CSNK1G3 (CK1 gamma 3)	ZLYTE	Km
258	28	MAP3K5 (ASK1)	LanthaScreen Binding - Eu-anti-GST	n.a.
259	28	NEK4	ZLYTE	Km
260	28	RPS6KA5 (MSK1)	ZLYTE	Km
261	27	CDK7/cyclin H/MNAT1	Adapta	Km
262	27	KIT V560G	ZLYTE	Km
263	27	MAPK3 (ERK1)	ZLYTE	Km
264	26	MAP2K4 (MEK4)	LanthaScreen Binding - Eu-anti-GST	n.a.

265	25	ACVRL1 (ALK1)	LanthaScreen Binding - Eu-anti-GST	n.a.
266	25	FES (FPS)	ZLYTE	Km
267	25	KIT V654A	LanthaScreen Binding - Eu-anti-His	n.a.
268	25	MAPK9 (JNK2)	LanthaScreen Binding - Eu-anti-His	n.a.
269	25	PLK3	ZLYTE	Km
270	25	PTK2 (FAK)	ZLYTE	Km
271	25	ULK2	LanthaScreen Binding - Eu-anti-GST	n.a.
272	24	ABL1 T315I	ZLYTE	Km
273	24	ABL2 (Arg)	ZLYTE	Km
274	24	ALK	ZLYTE	Km
275	24	BTK	ZLYTE	Km
276	24	FGFR2 N549H	ZLYTE	Km
277	24	HIPK4	ZLYTE	Km
278	24	KIT D820E	LanthaScreen Binding - Eu-anti-GST	n.a.
279	24	SNF1LK2	ZLYTE	Km
280	24	STK39 (STLK3)	LanthaScreen Binding - Eu-anti-His	n.a.
281	24	TAOK3 (JIK)	LanthaScreen Binding - Eu-anti-GST	n.a.
282	23	ALK R1275Q	LanthaScreen Binding - Eu-anti-GST	n.a.

283	23	EPHA7	LanthaScreen Binding - Eu-anti-GST	n.a.
284	23	JAK1	ZLYTE	Km
285	23	LIMK1	LanthaScreen Binding - Eu-anti-His	n.a.
286	23	PDGFRB (PDGFR beta)	ZLYTE	Km
287	23	PIP5K1A	Adapta	10
288	22	FGFR4	ZLYTE	Km
289	22	MAPKAPK5 (PRAK)	ZLYTE	Km
290	22	TXK	ZLYTE	Km
291	21	ACVR2B	LanthaScreen Binding - Eu-anti-GST	n.a.
292	21	CDK5 (Inactive)	LanthaScreen Binding - Eu-anti-GST	n.a.
293	21	EPHA6	LanthaScreen Binding - Eu-anti-GST	n.a.
294	21	EPHB2	ZLYTE	Km
295	21	EPHB4	ZLYTE	Km
296	21	INSR	ZLYTE	Km
297	20	CAMK2A (CaMKII alpha)	ZLYTE	Km
298	20	CDK17/cyclin Y	ZLYTE	Km
299	20	CSNK1G1 (CK1 gamma 1)	ZLYTE	Km
300	20	EGFR (ErbB1) T790M L858R	ZLYTE	Km

301	20	ERBB4 (HER4)	ZLYTE	Km
302	20	HUNK	LanthaScreen Binding - Eu-anti-GST	n.a.
303	20	MAP3K10 (MLK2)	LanthaScreen Binding - Eu-anti-GST	n.a.
304	20	MAPK13 (p38 delta)	ZLYTE	Km
305	19	CHEK1 (CHK1)	ZLYTE	Km
306	19	KIT V559D V654A	ZLYTE	Km
307	19	MAPK1 (ERK2)	ZLYTE	Km
308	19	MAPK12 (p38 gamma)	ZLYTE	Km
309	19	MKNK2 (MNK2)	LanthaScreen Binding - Eu-anti-GST	n.a.
310	19	PIK3CG (p110 gamma)	Adapta	Km
311	19	PTK6 (Brk)	ZLYTE	Km
312	19	SGK2	ZLYTE	Km
313	19	SIK1	LanthaScreen Binding - Eu-anti-GST	n.a.
314	19	STK22D (TSSK1)	ZLYTE	Km
315	19	STK25 (YSK1)	ZLYTE	Km
316	18	EPHA5	ZLYTE	Km
317	18	GRK5	ZLYTE	Km
318	18	MAP2K6 (MKK6) S207E T211E	LanthaScreen Binding - Eu-anti-His	n.a.

319	18	MLK4	LanthaScreen Binding - Eu-anti-GST	n.a.
320	18	MUSK	ZLYTE	Km
321	18	PIM2	ZLYTE	Km
322	18	WEE1	LanthaScreen Binding - Eu-anti-GST	n.a.
323	17	AURKC (Aurora C)	ZLYTE	Km
324	17	CDK13/cyclin K	LanthaScreen Binding - Eu-anti-GST	n.a.
325	17	CSNK2A2 (CK2 alpha 2)	ZLYTE	Km
326	17	IKBKE (IKK epsilon)	ZLYTE	Km
327	17	NEK1	ZLYTE	Km
328	17	PDK1 Direct	ZLYTE	Km
329	17	PRKACG (PRKAC gamma)	LanthaScreen Binding - Eu-anti-GST	n.a.
330	16	ERN1	LanthaScreen Binding - Eu-anti-GST	n.a.
331	16	LTK (TYK1)	ZLYTE	Km
332	16	NEK8	LanthaScreen Binding - Eu-anti-GST	n.a.
333	16	SRMS (Srm)	ZLYTE	Km
334	15	ACVR1 (ALK2)	LanthaScreen Binding - Eu-anti-GST	n.a.
335	15	ALK L1196M	LanthaScreen Binding - Eu-anti-GST	n.a.
336	15	BRSK2	LanthaScreen Binding - Eu-anti-GST	n.a.

337	15	MAPK10 (JNK3)	ZLYTE	100
338	15	MAPK8 (JNK1)	ZLYTE	10
339	15	NLK	LanthaScreen Binding - Eu-anti-GST	n.a.
340	15	PAK4	ZLYTE	Km
341	15	PDK1	ZLYTE	100
342	15	PRKCH (PKC eta)	ZLYTE	Km
343	15	TESK1	LanthaScreen Binding - Eu-anti-GST	n.a.
344	15	TNK2 (ACK)	LanthaScreen Binding - Eu-anti-GST	n.a.
345	14	ABL1 F317I	ZLYTE	Km
346	14	ACVR1 (ALK2) R206H	LanthaScreen Binding - Eu-anti-GST	n.a.
347	14	BMX	ZLYTE	Km
348	14	CAMK1G (CAMKI gamma)	ZLYTE	Km
349	14	CDK6/cyclin D1	Adapta	10
350	14	CSNK2A1 (CK2 alpha 1)	ZLYTE	Km
351	14	MET D1228H	LanthaScreen Binding - Eu-anti-GST	n.a.
352	14	PIK3C2A (PI3K-C2 alpha)	Adapta	Km
353	14	PKMYT1	LanthaScreen Binding - Eu-anti-GST	n.a.
354	14	PRKCA (PKC alpha)	ZLYTE	Km

355	14	PRKCG (PKC gamma)	ZLYTE	Km
356	14	WNK2	LanthaScreen Binding - Eu-anti-GST	n.a.
357	13	CDC42 BPA (MRCKA)	ZLYTE	Km
358	13	CDK11 (Inactive)	LanthaScreen Binding - Eu-anti-GST	n.a.
359	13	EGFR (ErbB1) T790M C797S L858R	ZLYTE	Km
360	13	EPHA3	LanthaScreen Binding - Eu-anti-His	n.a.
361	13	IGF1R	ZLYTE	Km
362	13	PIP4K2A	Adapta	10
363	13	PRKCB2 (PKC beta II)	ZLYTE	Km
364	13	RIPK2	LanthaScreen Binding - Eu-anti-His	n.a.
365	13	TEK (Tie2)	ZLYTE	Km
366	12	CAMK2B (CaMKII beta)	ZLYTE	Km
367	12	CDC42 BPG (MRCKG)	ZLYTE	Km
368	12	CDK4/cyclin D1	Adapta	10
369	12	MAPK14 (p38 alpha)	ZLYTE	100
370	12	MAPK9 (JNK2)	ZLYTE	100
371	12	MARK2	ZLYTE	Km
372	12	MET M1250T	ZLYTE	Km

373	12	RPS6KB1 (p70S6K)	ZLYTE	Km
374	12	STK38 (NDR)	LanthaScreen Binding - Eu-anti-GST	n.a.
375	11	EGFR (ErbB1) G719S	ZLYTE	Km
376	11	LIMK2	LanthaScreen Binding - Eu-anti-GST	n.a.
377	11	MAP2K6 (MKK6)	ZLYTE	100
378	11	MATK (HYL)	ZLYTE	Km
379	11	PRKG1	ZLYTE	Km
380	11	ROCK1	ZLYTE	Km
381	11	TEK (TIE2) R849W	LanthaScreen Binding - Eu-anti-GST	n.a.
382	10	CAMK1 (CaMK1)	Adapta	10
383	10	CAMKK2 (CaMKK beta)	LanthaScreen Binding - Eu-anti-GST	n.a.
384	10	CDC42 BPB (MRCKB)	ZLYTE	Km
385	10	EPHA8	ZLYTE	Km
386	10	FLT1 (VEGFR1)	ZLYTE	Km
387	10	PLK1	ZLYTE	Km
388	10	PRKCE (PKC epsilon)	ZLYTE	Km
389	10	TGFBR1 (ALK5)	LanthaScreen Binding - Eu-anti-GST	n.a.
390	9	ABL1 F317L	ZLYTE	Km

391	9	CSK	ZLYTE	Km
392	9	EGFR (ErbB1) L861Q	ZLYTE	Km
393	9	MARK1 (MARK)	ZLYTE	Km
394	9	NIM1K	ZLYTE	Km
395	9	PIK3C3 (hVPS34)	Adapta	Km
396	9	PIK3CB/PIK3R1 (p110 beta/p85 alpha)	Adapta	Km
397	9	SGKL (SGK3)	ZLYTE	Km
398	8	ACVR2A	LanthaScreen Binding - Eu-anti-GST	n.a.
399	8	BMPR1B (ALK6)	LanthaScreen Binding - Eu-anti-GST	n.a.
400	8	CAMK1D (CaMKI delta)	ZLYTE	Km
401	8	CAMKK1 (CAMKKA)	LanthaScreen Binding - Eu-anti-His	n.a.
402	8	EGFR (ErbB1) G719C	ZLYTE	Km
403	8	ERN2	LanthaScreen Binding - Eu-anti-GST	n.a.
404	8	MAPK11 (p38 beta)	ZLYTE	Km
405	8	MAPK7 (ERK5)	ZLYTE	Km
406	8	NEK6	ZLYTE	Km
407	8	PAK7 (KIAA1264)	ZLYTE	Km

408	8	PHKG2 - ZLYTE	ZLYTE	Km
409	8	PRKACA (PKA)	ZLYTE	Km
410	8	PRKCB1 (PKC beta I)	ZLYTE	Km
411	8	RPS6KB2 (p70S6Kb)	ZLYTE	Km
412	8	STK38L (NDR2)	LanthaScreen Binding - Eu-anti-GST	n.a.
413	7	EEF2K	ZLYTE	Km
414	7	EPHA4	ZLYTE	Km
415	7	KSR2	ZLYTE	Km
416	7	MET (cMet)	ZLYTE	Km
417	7	MKNK1 (MNK1)	ZLYTE	Km
418	7	PRKCD (PKC delta)	ZLYTE	Km
419	7	PRKCQ (PKC theta)	ZLYTE	Km
420	7	PRKCZ (PKC zeta)	ZLYTE	Km
421	7	PRKX	ZLYTE	Km
422	7	SIK3	LanthaScreen Binding - Eu-anti-GST	n.a.
423	7	SRPK1	ZLYTE	Km
424	7	STK32B (YANK2)	LanthaScreen Binding - Eu-anti-GST	n.a.
425	7	WNK1	LanthaScreen Binding - Eu-anti-His	n.a.

426	6	AKT1 (PKB alpha)	ZLYTE	Km
427	6	AKT2 (PKB beta)	ZLYTE	Km
428	6	BMPR1A (ALK3)	LanthaScreen Binding - Eu-anti-GST	n.a.
429	6	BRAF	LanthaScreen Binding - Eu-anti-GST	n.a.
430	6	BRAF V599E	LanthaScreen Binding - Eu-anti-GST	n.a.
431	6	DCAMKL2 (DCK2)	ZLYTE	Km
432	6	FRAP1 (mTOR)	ZLYTE	Km
433	6	KIT	ZLYTE	Km
434	6	MAPK14 (p38 alpha) Direct	ZLYTE	Km
435	6	MST1R (RON)	ZLYTE	Km
436	6	PAK1	ZLYTE	Km
437	6	PAK2 (PAK65)	ZLYTE	Km
438	6	PDGFRA T674I	ZLYTE	Km
439	6	PRKCI (PKC iota)	ZLYTE	Km
440	6	SRPK2 - ZLYTE	ZLYTE	Km
441	6	TEK (TIE2) Y1108F	LanthaScreen Binding - Eu-anti-GST	n.a.
442	5	CDK4/cyclin D3	Adapta	10
443	5	CDKL5	ZLYTE	Km

444	5	DDR1	LanthaScreen Binding - Eu-anti-GST	n.a.
445	5	PAK3	ZLYTE	Km
446	5	STK22B (TSSK2)	ZLYTE	Km
447	4	EGFR (ErbB1)	ZLYTE	Km
448	4	EGFR (ErbB1) C797S	ZLYTE	Km
449	4	KIT T670I	ZLYTE	Km
450	4	NEK9	ZLYTE	Km
451	4	PAK6	ZLYTE	Km
452	4	PKN1 (PRK1)	ZLYTE	Km
453	4	RAF1 (cRAF) Y340D Y341D	LanthaScreen Binding - Eu-anti-GST	n.a.
454	4	ROCK2	ZLYTE	Km
455	4	STK23 (MSSK1)	ZLYTE	Km
456	4	TEC	LanthaScreen Binding - Eu-anti-His	n.a.
457	4	ZAK	LanthaScreen Binding - Eu-anti-GST	n.a.
458	3	AKT3 (PKB gamma)	ZLYTE	Km
459	3	DDR2	LanthaScreen Binding - Eu-anti-GST	n.a.
460	3	FGFR3	ZLYTE	Km
461	3	MAPKAPK3	ZLYTE	Km

462	3	TAOK2 (TAO1)	ZLYTE	Km
463	3	TESK2	LanthaScreen Binding - Eu-anti-GST	n.a.
464	2	DCAMKL1 (DCLK1)	ZLYTE	Km
465	2	ERBB2 (HER2)	ZLYTE	Km
466	2	MAP2K6 (MKK6)	LanthaScreen Binding - Eu-anti-His	n.a.
467	2	MAPKAPK2	ZLYTE	Km
468	2	MET (cMet) Y1235D	ZLYTE	Km
469	2	WNK3	LanthaScreen Binding - Eu-anti-His	n.a.
470	1	PI4K2A (PI4K2 alpha)	Adapta	Km
471	1	PI4K2B (PI4K2 beta)	Adapta	Km
472	1	SBK1	ZLYTE	Km
473	1	STK32C (YANK3)	LanthaScreen Binding - Eu-anti-GST	n.a.
474	1	TEK (TIE2) Y897S	ZLYTE	Km
475	0	ADRBK2 (GRK3)	ZLYTE	Km
476	0	PI4KA (PI4K alpha)	Adapta	10
477	0	ZAP70 - ZLYTE	ZLYTE	Km
478	-1	EGFR (ErbB1) L858R	ZLYTE	Km
479	-1	SPHK2	Adapta	10

480	-3	ADRBK1 (GRK2)	ZLYTE	Km
481	-3	CASK	LanthaScreen Binding - Eu-anti-GST	n.a.
482	-3	SPHK1	Adapta	Km
483	-6	RIPK3	LanthaScreen Binding - Eu-anti-GST	n.a.
484	-9	EPHB3	ZLYTE	Km
485	-11	NEK2	NEK2 - ZLYTE	Km
486	-13	ACVR1B (ALK4)	ACVR1B (ALK4) - ZLYTE	Km
